# Prolyl 4‐hydroxylase subunit alpha 1 (P4HA1) is a biomarker of poor prognosis in primary melanomas, and its depletion inhibits melanoma cell invasion and disrupts tumor blood vessel walls

**DOI:** 10.1002/1878-0261.12649

**Published:** 2020-02-28

**Authors:** Johanna Eriksson, Vadim Le Joncour, Tiina Jahkola, Susanna Juteau, Pirjo Laakkonen, Olli Saksela, Erkki Hölttä

**Affiliations:** ^1^ Department of Pathology University of Helsinki Finland; ^2^ Faculty of Medicine Translational Cancer Medicine Research Program University of Helsinki Finland; ^3^ Department of Plastic Surgery Helsinki University Hospital Finland; ^4^ Department of Pathology University of Helsinki and Helsinki University Hospital Finland; ^5^ Laboratory Animal Center HiLIFE – Helsinki Institute of Life Science University of Helsinki Finland; ^6^ Department of Dermatology Helsinki University Hospital Finland

**Keywords:** CTHRC1, invasion, melanoma, P4HA1, prognosis

## Abstract

Melanoma is an unpredictable, highly metastatic malignancy, and treatment of advanced melanoma remains challenging. Novel molecular markers based on the alterations in gene expression and the molecular pathways activated or deactivated during melanoma progression are needed for predicting the course of the disease already in primary tumors and for providing new targets for therapy. Here, we sought to identify genes whose expression in primary melanomas correlate with patient disease‐specific survival using global gene expression profiling. Many of the identified potential markers of poor prognosis were associated with the epithelial–mesenchymal transition, extracellular matrix formation, and angiogenesis. We studied further the significance of one of the genes, prolyl 4‐hydroxylase subunit alpha 1 (P4HA1), in melanoma progression. P4HA1 depletion in melanoma cells reduced cell adhesion, invasion, and viability *in vitro*. In melanoma xenograft assays, we found that P4HA1 knockdown reduced melanoma tumor invasion as well as the deposition of collagens, particularly type IV collagen, in the interstitial extracellular matrix and in the basement membranes of tumor blood vessels, leading to vessel wall rupture and hemorrhages. Further, P4HA1 knockdown reduced the secretion of collagen triple helix repeat containing 1 (CTHRC1), an important mediator of melanoma cell migration and invasion, *in vitro* and its deposition around tumor blood vessels *in vivo*. Taken together, P4HA1 is an interesting potential prognostic marker and therapeutic target in primary melanomas, influencing many aspects of melanoma tumor progression.

## Graphical abstract

We have identified several prognostic markers and potential therapeutic targets in primary melanomas. One of them, prolyl 4‐hydroxylase subunit alpha 1 (P4HA1), proved critical for the invasion and survival of melanoma cells and the deposition of collagens (stained in blue) and CTHRC1 in the extracellular matrix and tumor blood vessel walls, maintaining their integrity.

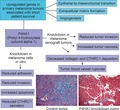

**Abbreviations**
COL‐I: type I collagen
COL‐IV: type IV collagen
DHB: 3,4‐dihydroxybenzoic acid
GSEA: gene set enrichment analysis
SAM: significance analysis of microarrays

## 1 Introduction

Cutaneous melanoma is one of the most aggressive malignancies, and its incidence is still rising all over the world. Breslow’s tumor thickness, mitotic rate, and ulceration are considered to be the best prognostic factors predicting survival for patients diagnosed with localized disease (Balch *et al.*, 2009). However, over 7% of patients with thin melanomas (Breslow’s thickness 0.51–1.0 mm) and a mitotic rate < 1.00 mitoses·mm^−2^ die within 10 years of diagnosis (Thompson *et al.*, 2011). These cases show that the survival outcome cannot be predicted on histopathological features alone. Further, the standard staging method for regional metastatic melanoma, the lymphatic mapping with invasive sentinel lymph node biopsy (and in case of a positive sentinel lymph node biopsy, completion lymphadenectomy), is associated with substantial morbidity. Additionally, about 10% of sentinel lymph node‐negative patients with intermediate thickness (1.2–3.5 mm) primary melanomas and about 30% of sentinel lymph node‐negative patients with thick (> 3.5 mm) primary melanomas die of their disease within 5 years of diagnosis (Morton *et al.*, 2014), suggesting the presence of false‐negative results or spreading of the disease through other routes, such as the blood circulation. Also, despite the recent advances in targeted and immunotherapies, the treatment of advanced melanoma remains challenging. Therapies targeting the mutated BRAF are associated with high response rates, but only a subset of patients show prolonged disease control. Newer immunotherapies, in turn, induce more durable disease control in majority of the responders but have lower response rates (Silva and Long, 2017). The clinical and biological heterogeneity of melanomas calls for novel, better molecular markers based on the molecular pathways activated or deactivated in melanoma progression for predicting the course of the disease and providing new targets for therapy.

Here, we sought to identify new molecules associated with melanoma progression and aggressiveness that could predict patient survival already in primary melanomas. We found that many of the identified potential prognostic marker genes were associated with the epithelial–mesenchymal transition, extracellular matrix organization, collagen formation, and angiogenesis. Among others, high mRNA expression of the hypoxia‐regulated P4HA1 gene, encoding the catalytic subunit of prolyl 4‐hydroxylase, correlated highly significantly with shorter patient survival in an independent primary melanoma dataset. We further studied the function of P4HA1 in melanoma by knocking it down and analyzing the effect on melanoma cell adhesion, invasion, and viability *in vitro* and tumorigenesis *in vivo*. We also show that prolyl 4‐hydroxylase function is essential for the secretion of collagen triple helix repeat containing 1 (CTHRC1), an important mediator of melanoma cell migration and invasion.

## 2 Materials and methods

### 2.1 Patient samples

Primary cutaneous melanomas (*n* = 44) and benign nevi (*n* = 19) from healthy volunteers were obtained by surgical excision at Helsinki University Central Hospital using protocols approved by the Ethics Committee of Helsinki University Central Hospital. The study conformed to the standards set by the Declaration of Helsinki, and all patients provided informed consent. Half of each tissue specimen was fixed in formalin for histopathological and immunohistochemical analyses, and the other half was immediately frozen in liquid nitrogen or immersed in RNAlater RNA stabilization solution (Invitrogen/Thermo Fisher Scientific, Waltham, MA, USA) for gene expression analyses using DNA microarrays and RT–PCR.

### 2.2 RNA isolation and purification

RNA was extracted from cells and tissues using the RNeasy kit (> 200 nt RNA, Qiagen, Hilden, Germany). Frozen tissue specimens were ground in liquid nitrogen and homogenized in lysis buffer with a 21‐gauge needle. Tissues immersed in RNAlater were homogenized in lysis buffer with Lysing matrix D and the FastPrep FP120 Cell Disrupter (Qbiogene/MP Biomedicals, Santa Ana, CA, USA) according to the manufacturer’s instructions. Pigment removal was performed by adsorption to Bio‐Gel P‐60 as described elsewhere (Soikkeli *et al.*, 2007). The quality of the purified RNA was assessed by agarose gel electrophoresis or by Bioanalyzer 2100 (Agilent Technologies, Santa Clara, CA, USA).

### 2.3 Microarray analysis and data analysis

Primary melanomas (*n* = 19) and benign nevi (*n* = 11) were analyzed using the Human Genome U133 Plus 2.0 array and normal melanocytes and melanoma cell lines (WM793 and WM239) using the Human Genome U133 set arrays (Affymetrix/Thermo Fisher Scientific), as previously described (Soikkeli *et al.*, 2007).

The microarray probe signals were preprocessed using the RMA algorithm (Bolstad *et al.*, 2003) in the Chipster v3.11.6 software (http://chipster.csc.fi/). Gene probe sets with a mean difference < 100 and a fold change < 1.5 between the melanoma sample groups (alive or died of melanoma) were filtered off before searching for genes correlating with patient survival using Significance Analysis of Microarrays (SAM) 5.0 package (Tusher *et al.*, 2001) (http://www-stat.stanford.edu/~tibs/SAM/). SAM survival analysis was performed in R (version 3.4.0) (http://www.r-project.org/) using 4000 random permutations. Delta was chosen so that the false discovery rate was < 1%.

Gene lists were subjected to Gene Set Enrichment Analysis (GSEA) using the Molecular Signature Database (MSigDB, v6.0, Broad Institute, MA, USA, http://www.broadinstitute.org/gsea/msigdb/index.jsp) Hallmark gene sets. False discovery rate *q*‐values < 0.05 were considered significant. Correlating gene expression was analyzed with Pearson correlation using 0.35 as a cutoff value.

### 2.4 RT–PCR analyses

One microgram of RNA was reverse‐transcribed into cDNA and used for semiquantitative PCR analysis as previously described (Soikkeli *et al.*, 2007). Primers and PCR variables are listed in Table S1.

### 2.5 Histology and immunohistochemistry

Formalin‐fixed paraffin‐embedded sections (5 µm) were deparaffinized, rehydrated in a graded ethanol series, and subjected to routine hematoxylin and eosin (H&E) staining and Masson’s trichrome staining to discriminate collagen fibers from muscular tissues. For P4HA1 staining, sections were subjected to heat‐induced epitope retrieval (HIER) in citrate buffer (0.01 m, pH 6.0). HIER in Tris/EDTA buffer, pH 9.0, and trypsin treatment were also tested as the antigen retrieval methods in the evaluation phase of P4HA1 staining, the former giving equivalent results with the citrate buffer. The endogenous peroxidase activity was blocked with peroxidase‐blocking solution (Dako/Agilent Technologies). A panel of P4HA1 antibodies (12658‐1‐AP, 1 : 100, and 66101‐1‐Ig, 1 : 500 from Proteintech Group, Rosemont, IL, USA; NBP1‐84398, 1 : 1000 from Novus Biologicals, Littleton, CO, USA; HPA007599, 1 : 200 from Atlas Antibodies, Bromma, Sweden) were tested for their performance in immunohistochemistry using primary melanoma samples showing high P4HA1 mRNA expression (Fig. S1), and the mouse monoclonal antibody 66101‐1‐Ig was selected for the study. Sections were incubated with the primary antibody (1 : 500) in antibody diluent (Immunologic, Duiven, The Netherlands) for 1 h and detected using BrightVision Poly‐HRP kit (Immunologic) and 3‐amino‐9‐ethylcarbazole (AEC) as the chromogen. For cleaved caspase 3 staining, sections were subjected to HIER in citrate buffer (0.01 m, pH 6.0), blocked with CAS‐block containing 1.5% goat serum for 1 h, incubated with the rabbit monoclonal antibody (#9664, Cell Signaling Technology, Leiden, The Netherlands; 1 : 200) in PBS containing 0.1% goat serum at +4 °C overnight, and detected with the Vectastain ABC kit (Vector Laboratories, Burlingame, CA, USA) according to the manufacturer’s protocol using AEC as the chromogen. For CD31 staining, sections were subjected to HIER in Tris/EDTA buffer (pH 9.0) and incubated in a Tris buffer (pH 7.5) containing 0.05% Triton X‐100 for 10 min. Endogenous peroxidase activity was blocked with 0.3% H_2_O_2_ in PBS for 10 min. Sections were blocked in 2.5% goat serum in PBS for 1 h, incubated with a rabbit polyclonal CD31 antibody (ab124432, Abcam, 1 : 500) in PBS containing 0.1% goat serum at +4 °C overnight, and detected with the Vectastain ABC kit using AEC. For type IV collagen (COL‐IV) staining, sections were subjected to HIER in citrate buffer (CC2, Ventana/Roche, Basel, Switzerland) and protease treatment with protease 1 (Ventana/Roche). Sections were incubated with a mouse monoclonal COL‐IV antibody (clone CIV22, ready‐to‐use, Ventana/Roche) and detected using the ultraView Universal DAB Detection kit (Ventana/Roche). For Ki‐67 staining, sections were subjected to HIER in Tris‐based buffer (CC1, Ventana/Roche), incubated with a mouse monoclonal Ki‐67 antibody (MIB‐1, Dako/Agilent Technologies) and Amplification kit reagents (Ventana/Roche), and detected with the ultraView Universal DAB Detection kit. Ki‐67/MIB‐1 labeling index was assessed visually/microscopically by counting the percentage of positively stained nuclei in five randomly selected high‐power magnification (400×) fields.

Frozen sections (5 µm) for CTHRC1 detection were fixed with 4% paraformaldehyde for 25 min, permeabilized in 0.1% Triton X‐100 in PBS for 10 min, and incubated in peroxidase‐blocking solution (Dako/Agilent Technologies) for 10 min. Sections were blocked in 5% BSA and 10% normal goat serum in PBS for 1 h and incubated with the rabbit polyclonal antibody against CTHRC1 (11647‐RP02, Sino Biological Inc., Beijing, China, 1 : 780) in PBS containing 5% normal goat serum at 4 °C overnight. In type I collagen (COL‐I) staining, frozen sections were incubated in 0.3% H_2_O_2_ in methanol for 30 min and blocked in 5% normal goat serum. Sections were then incubated with the mouse monoclonal antibody to COL‐I (ab6308, Abcam, 1 : 2500) in PBS containing 5% normal goat serum at 4 °C overnight. Immunodetection was performed using the Vectastain ABC kit (Vector Laboratories) according to the manufacturer’s protocol using AEC as the chromogen.

As negative controls, the primary antibodies were replaced by normal (isotype control) mouse or rabbit IgG in all stainings. All sections were counterstained with Mayer’s hematoxylin and mounted with Faramount (Dako/Agilent Technologies). Images were taken using a Nikon Eclipse 80i microscope, a Digital Sight DS‐5M camera, and the nis‐elements f 2.20 software (Nikon, Tokyo, Japan). Tissue sections stained for cleaved caspase 3 were scanned using the 3DHISTECH Pannoramic 250 FLASH II digital slide scanner (3DHISTECH Ltd., Budapest, Hungary) at the Genome Biology Unit (University of Helsinki and Biocenter Finland). Quantification of the cleaved caspase 3‐positive cells in the whole tumor sections was performed using the HistoQuant module of the caseviewer (3DHISTECH) and fiji imagej 1.51.

### 2.6 TUNEL assay

Apoptotic and necrotic cells with DNA strand breaks were analyzed with TUNEL (terminal deoxynucleotidyl transferase (TdT) dUTP Nick‐End Labeling) staining using the In Situ Cell Death Detection Kit, TMR red (Roche) according to the manufacturer’s recommendations. Briefly, formalin‐fixed paraffin‐embedded sections were first deparaffinized and rehydrated in graded alcohol series. Sections were then treated with proteinase K (15 µg·mL^−1^ in 10 mm Tris/HCl, pH 7.5) at +37 °C for 25 min and incubated with the TUNEL reaction mixture at +37 °C for 1 h. Finally, sections were stained with DAPI and mounted with Mowiol (Sigma‐Aldrich). Images were generated using 3DHISTECH Pannoramic 250 FLASH II digital slide scanner at the Genome Biology Unit (University of Helsinki and Biocenter Finland). Quantification of the TUNEL‐ and DAPI‐positive cells in the whole tumor sections was performed using fiji imagej 1.51. TUNEL‐positive cells were quantified as the percentage of the total number of cells (nuclei stained by DAPI) per tumor sections in all xenograft tumors.

### 2.7 Western blotting

We performed the analysis of proteins from whole‐cell extracts (Kielosto *et al.*, 2004) and conditioned media (Nummela *et al.*, 2012; Ravanko *et al.*, 2004) as previously described. Briefly, proteins in 1× Laemmli sample buffer were resolved in 10% polyacrylamide gels and transferred on to nitrocellulose (Bio‐Rad, Hercules, CA, USA) or PVDF membranes (Immobilon‐FL, Merck, Darmstadt, Germany). After blocking with 2% BSA, membranes were incubated with the primary antibodies overnight at +4 °C. A panel of P4HA1 antibodies (NB100‐57852 and NBP1‐84398 from Novus Biologicals; 12658‐1‐AP and 66101‐1‐Ig from Proteintech Group) were tested for their performance in western blotting, and the goat pAb NB100‐57852 was selected for the study. The specificity of the antibodies was validated by analyzing the P4HA1 protein in control shRNA and P4HA1‐knockdown (P4HA1‐KD) cells. In addition, rabbit pAb ab85739 (Abcam, Cambridge, UK) was used to detect CTHRC1 protein. Mouse mAbs to alpha‐tubulin (DM1A, Abcam) and alpha‐actin (JLA20, Merck) were used as loading controls. Immunodetection was performed with chemiluminescence using horseradish peroxidase‐conjugated secondary antibodies (Dako/Agilent Technologies) and the Clarity Western ECL Substrate (Bio‐Rad) or with the Odyssey imaging system using IRDye secondary antibodies (LI‐COR Biotechnology, Lincoln, NE, USA). The densities of the protein bands were quantified using ImageJ (1.47v; NIH, Bethesda, MD, USA) or Image Studio Lite (LI‐COR Biotechnology, Lincoln, NE, USA).

### 2.8 Cell culture and reagents

We isolated and cultured primary human melanocytes as described elsewhere (Alanko *et al.*, 1999). Melanoma cell lines as well as the primary human embryonic fibroblasts and microvascular endothelial cells were obtained and cultured as previously described (Eriksson *et al.*, 2016).

The prolyl 4‐hydroxylase inhibitor 3,4‐dihydroxybenzoic acid (DHB) was purchased from Abcam (ab142937).

### 2.9 Short hairpin RNA lentiviral particle transduction

WM239 and SKMEL‐28 melanoma cells were transduced with Mission lentiviral transduction particles (SHCLNV) targeting P4HA1 (TRCN 0000303933) (P4HA1 shRNA cell pools 1 and 2) or with Mission TRC2 pLK0.5‐puro nontarget shRNA control particles (SHC216V) from Sigma‐Aldrich/Merck, or with shRNA lentiviral particles targeting P4HA1 (sc‐90782‐V) (P4HA1 shRNA cell pools 3 and 4) or with control shRNA particles (sc‐108080) from Santa Cruz Biotechnology (Dallas, TX, USA) according to the manufacturer’s instructions. Pools of puromycin‐resistant cells were used in the assays.

### 2.10 Transfection of CRISPR double‐nickase plasmids

WM239 and SKMEL‐28 cells were transfected with P4HA1 double‐nickase plasmids (sc‐407173‐NIC, Santa Cruz Biotechnology) or with control double‐nickase plasmids (sc‐437281) using Lipofectamine 3000 (Thermo Fisher Scientific) according to manufacturer’s instructions. After puromycin selection, GFP‐positive cell colonies were cylinder‐cloned and assayed for P4HA1 downregulation by western blot.

### 2.11 RNA sequencing and data analysis

RNA sequencing library preparation, sequencing, and data analysis were performed at the Biomedicum Functional Genomics Unit (FuGU, University of Helsinki, Finland). Shortly, RNA sequencing libraries were prepared from two WM239 parental cell pools, control shRNA pools 2 and 3, and P4HA1 shRNA pools 1‐4 using the NEBNext Ultra II Directional RNA Library Prep‐kit (New England Biolabs, Ipswich, MA, USA) according to the manufacturer’s instructions after purification of mRNA with polyA‐binding beads. Sequencing was performed with Illumina NextSeq sequencer (Illumina, San Diego, CA, USA) using a 75‐bp pair‐end Mid Output sequencing kit. RNA sequencing data quality was assessed with FastQC, and the data were quality‐trimmed with Trimmomatic. Reads were aligned to the genome (hg38, GRCh38.p12) with STAR software, and gene counts were quantified with featureCounts (using gencode release 28 annotations). Differentially expressed genes (Benjamini–Hochberg‐adjusted *P*‐value < 0.05) were identified using the deseq2 software package (http://bioconductor.org/) in r.

### 2.12 Immunofluorescence staining

Cells were grown on glass coverslips in normal growth medium for 4 days and incubated in serum‐free medium for 24 h, after which the cells were fixed with 4% paraformaldehyde for 30 min. After permeabilization with 0.1% Triton X‐100 in PBS for 10 min and blocking with 1% BSA (Invitrogen/Thermo Fisher Scientific) and 10% normal goat serum in PBS for 1 h, the cells were incubated with 1.5 µg·mL^−1^ of rabbit pAb to P4HA1 (12658‐1‐AP, Proteintech Group) diluted in PBS containing 5% normal goat serum for 2 h. Then, cells were washed three times with PBS and incubated with Alexa Fluor 488‐conjugated goat anti‐rabbit secondary antibodies (Invitrogen/Thermo Fisher Scientific) in PBS containing 1% normal goat serum. F‐actin was detected with the Alexa Fluor 594‐conjugated phalloidin (Molecular Probes/Thermo Fisher Scientific). Finally, the cells were mounted with the Vectashield H‐1200 mounting media containing DAPI (Vector Laboratories), and images were obtained using a Zeiss Axiophot2 epifluorescence microscope (Carl Zeiss, Oberkochen, Germany), QImaging Retiga 4000R digital camera, and QCapture Pro 6‐software (QImaging, Surrey, Canada).

### 2.13 Cell adhesion assay

Flat‐bottomed 96‐well plates were coated with 100 μL of 10 μg·mL^−1^ COL‐I (BD Biosciences, Franklin Lakes, NJ, USA) or cellular fibronectin (USBiologicals, Salem, MA, USA) for 2 h at 37 °C, and washed three times with PBS. Cells (2.5 × 10^4^) were added to uncoated or COL‐I‐ or fibronectin‐coated wells in serum‐free RPMI 1640 medium (Sigma‐Aldrich/Merck). Cell attachment and spreading were recorded by photography, and the cells with different morphologies were counted after 2‐ to 72‐h incubation at 37 °C. Attached, spread cells were defined as cells with flattened or elongated morphology, and round cells were considered unattached.

### 2.14 Cell apoptosis and viability assay

Cells used in the cell adhesion assay were stained with the CellEvent Caspase‐3/7 Green Ready Probes Reagent to visualize apoptotic cells and with the cell membrane‐impermeable propidium iodide from the Ready Probes Cell Viability Imaging Kit, blue/red (both from Thermo Fisher Scientific) to visualize dead cells with porous membrane according to the manufacturer’s instructions. Images of the cells were obtained using a Olympus IX71 microscope and DP70 camera (Olympus, Center Valley, Philadelphia, PA, USA).

### 2.15 Three‐dimensional Matrigel invasion assays

Melanoma cell invasion was assayed in a 3D growth factor‐reduced Matrigel (BD Biosciences) as previously described (Kääriäinen *et al.*, 2006; Ravanko *et al.*, 2004). Briefly, cells (3 × 10^4^) were cast between two Matrigel (3.3 mg·mL^−1^) layers, and the serum‐free melanocyte growth medium M2 supplemented with the growth factor Supplement Mix (PromoCell, Heidelberg, Germany) was added on top of the gel (replenished every third day). The invasion patterns of the cells were analyzed daily using microscopy and photography.

### 2.16 Xenograft tumor models

Animal studies were carried out according to the Animal Experiment Board in Finland (ELLA) for the care and use of animals under the licenses ESAVI‐6285‐04.10.07‐20151. WM239 cells [6 × 10^6^ cells in 100 µL of Opti‐MEM I (Gibco/Thermo Fisher Scientific)] expressing P4HA1 shRNA (pool 2; Sigma‐Aldrich/Merck shRNA) or control shRNA (pool 1) were injected subcutaneously into the lower flank of 5‐week‐old female NMRI‐Foxn1 nu/nu mice (*n* = 8–9/group, one inoculation per animal). Tumor growth was followed two to three times a week using a caliper (measurements in three dimensions), and the mice were euthanized at day 36 when the largest tumors reached the ethical limits. The tumors were removed, cut in half, and one half was fixed in formalin and embedded in paraffin, and the other half was frozen in isopentane. For histological and immunohistochemical analyses, the tumors were cut into 5‐µm sections and stained with H&E and Masson’s trichrome and antibodies against P4HA1, COL‐IV, COL‐I, CTHRC1, CD31, cleaved caspase 3, and Ki‐67 as described in Histology and Immunohistochemistry Section 2.5. In addition, the tumor sections were analyzed for apoptotic cell death and necrosis with the TUNEL assay (Section 2.6).

### 2.17 Statistical analysis

Statistical analyses were performed using a two‐tailed Welch’s *t*‐test, where we considered *P* < 0.05 significant. Survival curves were plotted according to the Kaplan–Meier method and compared using the log‐rank test in the spss 25.0.0.1 software program (SPSS, Chicago, IL, USA). Median mRNA expression value of each gene was used as a cutoff value to classify samples into groups with low and high gene expression. Univariable and bivariable Cox regression analyses were performed using the spss program.

## 3 Results

### 3.1 Microarray analysis of primary melanomas

We have previously performed a preliminary search for potential markers of poor prognosis in primary melanomas (Eriksson *et al.*, 2016). Here, we wanted to determine the gene expression profiles of aggressive primary melanomas and melanomas that showed a better outcome in a longer follow‐up study. The samples were obtained from patients who had died of melanoma in < 2 years after diagnosis (*n* = 7; median survival time 15 months, range 2–19 months; Breslow's thickness: average = 8.3 mm, median = 7.0 mm) or were alive with at least 2 years of follow‐up (*n* = 12; median follow‐up time = 90 months, range = 28–145 months; Breslow's thickness: average = 4.7 mm, median = 1.4 mm). We performed Significance Analysis of Microarrays (SAM) to find genes correlating with patient disease‐specific survival. SAM survival analysis identified 563 genes whose expression correlated with short patient survival time (Table S2) and 974 genes whose expression correlated with long survival time (Table S3). To determine whether these genes are specifically associated with certain processes and pathways, we performed a Gene Set Enrichment Analysis (GSEA) using the Hallmark gene set collection and found that the genes associated with short survival were especially over‐represented in the epithelial–mesenchymal transition (EMT)‐gene set (Table S4). In other gene set collections, our short survival genes were enriched among others in focal adhesion and extracellular matrix (ECM)–receptor interaction (KEGG pathways), ECM organization, and collagen formation (Reactome pathways), as well as angiogenesis (Gene Ontology Biological process) (Table S4).

To further clarify whether the above potential prognostic marker genes might be upregulated specifically in the malignant melanoma cells, we compared the expression levels of the marker genes in primary melanomas and normal nevi as well as in melanoma cell lines (WM793 and WM239) and normal 42V melanocytes (Tables S2 and S3). The most interesting, potentially malignancy‐associated genes associated with short survival were prolyl 4‐hydroxylase subunit alpha 1 (P4HA1), Ras homolog gene family, member C (RHOC), serpin peptidase inhibitor, clade A, member 3 (SERPINA3), and fibronectin (FN1). We then also analyzed the potential of our top survival marker genes as predictors of melanoma patients’ overall survival in an independent, publicly available RNA sequencing dataset of primary melanomas (GEO accession: http://www.ncbi.nlm.nih.gov/geo/query/acc.cgi?acc=GSE98394: *n* = 44). In this dataset, the median follow‐up time of the patients was 93 months, and the follow‐up time for long‐term survivors was at least 6 years. Median Breslow’s thickness of the primary melanomas was 2.6 (range 0.24–34) mm. We classified the patients into two groups according to the mRNA expression levels of each of the potential marker genes (the median expression values of all primary melanoma samples were used as cutoff values) and found that the Kaplan–Meier 10‐year overall survival curves differed highly significantly (log‐rank test, *P* < 0.001) between the groups when the expression levels of our short survival markers P4HA1, RHOC, SORT1, CD63, or CDK2AP1 (Fig. S2; Table S5, showing genes with *P* < 0.05) or long survival markers FOXN3, EGFR, DSC3, CLDN1, PTBP3, or CXADR (Fig. S3; Table S6; showing genes with *P* < 0.05) were used as classifiers. Expression values for SERPINA3 and SNHG5 were not reported in this data set. In univariable Cox regression analyses, the expression of FOXN3, EGFR, DSC3, CLDN1, PTBP3, or CXADR was significantly associated with longer overall survival (*P* < 0.01), and the expression of P4HA1, RHOC, SORT1, CD63, or CDK2AP1 was significantly associated with shorter overall survival (*P* < 0.01), as was Breslow’s thickness (*P* = 0.014), which was dichotomized using 2 mm as a cut‐point value (≤ 2 mm and > 2 mm). The expression of P4HA1, RHOC, SORT1, CD63, and CDK2AP1 remained significantly associated (*P* < 0.05) with shorter survival and EGFR, PTBP3, and CXADR with longer survival after adjusting for Breslow’s thickness in bivariable Cox regression models, suggesting that these genes are independent candidate markers of melanoma patient survival. We chose to study further P4HA1 in melanoma progression to assess its potential as a prognostic marker and therapeutic target.

### 3.2 P4HA1 is highly expressed in melanoma cells and fibroblasts

**Figure 1:**
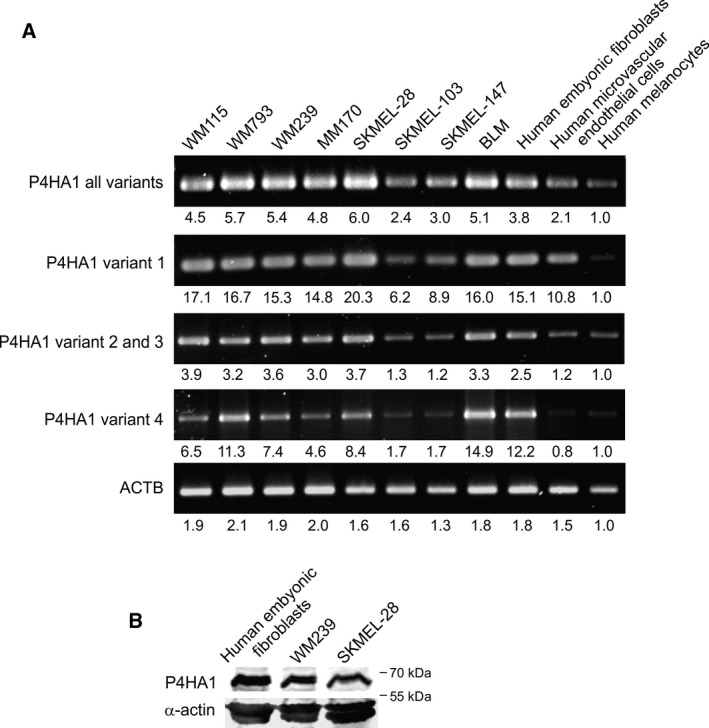
Expression of P4HA1 in melanoma cell lines and various primary cells. (A) Expression of different P4HA1 transcript variants in a panel of melanoma cell lines and different human primary cells analyzed by semiquantitative PCR. Beta‐actin (ACTB) was used as a reference. Relative expression levels of each gene or variant compared to normal melanocytes are shown below. (B) Western blot analysis of P4HA1 protein expression in cell extracts of human embryonic fibroblasts and WM239 and SKMEL‐28 melanoma cell lines. Alpha‐actin was used as a loading control.

P4HA1 encodes one of the alpha subunits of prolyl 4‐hydroxylase, a key enzyme in the synthesis of collagens, composed of two identical alpha subunits and two beta subunits. A dimer of P4HA1 forms the catalytically active site of prolyl 4‐hydroxylase I enzyme, which is the most common form in many cell types and tissues (reviewed in Myllyharju, 2003). As fibroblasts have been reported to be important producers of P4HA1, we wanted to confirm that P4HA1 is also expressed by melanoma cells. There are four P4HA1 transcript variants encoding three different P4HA1 protein isoforms, although it is not known if these isoforms have different functions. We first analyzed the mRNA expression levels of all P4HA1 transcript variants in melanoma cell lines and in primary cultures of human embryonic fibroblasts, microvascular endothelial cells, and normal melanocytes. P4HA1 variant 1 (encoding isoform 1) as well as variants 2 and 3 (encoding isoform 2) were expressed at a similar or higher level in most melanoma cell lines compared to fibroblasts (Fig. 1A). Fibroblasts showed a little higher expression of the shorter transcript variant 4 (encoding isoform 3) than most melanoma cell lines. Normal melanocytes and microvascular endothelial cells, in turn, showed lower expression of all the variants than most melanoma cell lines. We then analyzed the protein expression levels of human embryonic fibroblasts and two melanoma cell lines WM239 and SKMEL‐28, which expressed P4HA1 mRNA at high level. The antibody we used in western blot analyses recognizes the P4HA1 protein isoforms 1 and 2. At the protein level, embryonic fibroblasts showed higher expression of P4HA1 compared to the melanoma cell lines (Fig. 1B).

**Figure 2:**
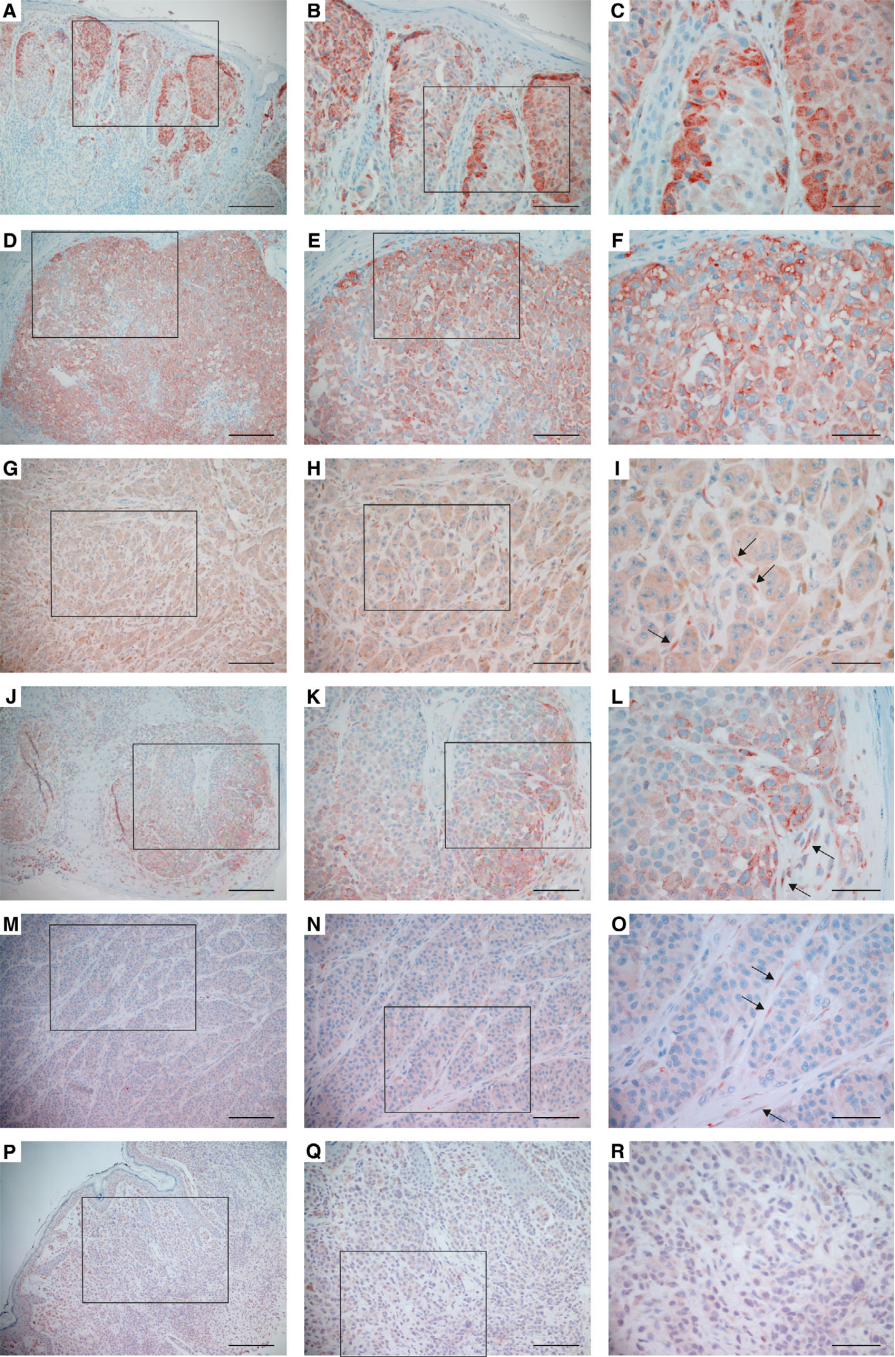
Expression of P4HA1 protein in primary melanomas and nevi. (A–O) Examples of primary melanomas that show different levels of P4HA1 expression in melanoma cells. Staining was also detected in the fibroblasts surrounding the melanoma cells in some samples (I, L, O, marked with arrows). (P–R) P4HA1 expression in a benign nevus. Positive immunostaining is shown in red. (B, E, H, K, N, Q) show a higher magnification of the boxed area in (A, D, G, J, M, P, respectively) and (C, F, I, L, O, R) in (B, E, H, K, N, Q, respectively). Scale bars = 200 µm (A, D, G, J, M, P), 100 µm (B, E, H, K, N, Q), 50 µm (C, F, I, L, O, R).

We also analyzed the expression and localization of P4HA1 in primary melanoma tissues (*n* = 27) by immunohistochemical staining. P4HA1 was mainly expressed by melanoma cells (Fig. 2A–O) but fairly often also by fibroblasts surrounding the melanoma cell nests (Fig. 2G–O), and by fibroblasts in the upper dermis (Fig. S1J,K). The intensity of the staining varied from negative to strong positive even in individual melanoma samples. Staining in normal nevi (*n* = 8) varied from negative to moderate (Fig. 2P–R).

### 3.3 Knockdown of P4HA1 reduces cell adhesion, invasion, and survival of melanoma cells

**Figure 3:**
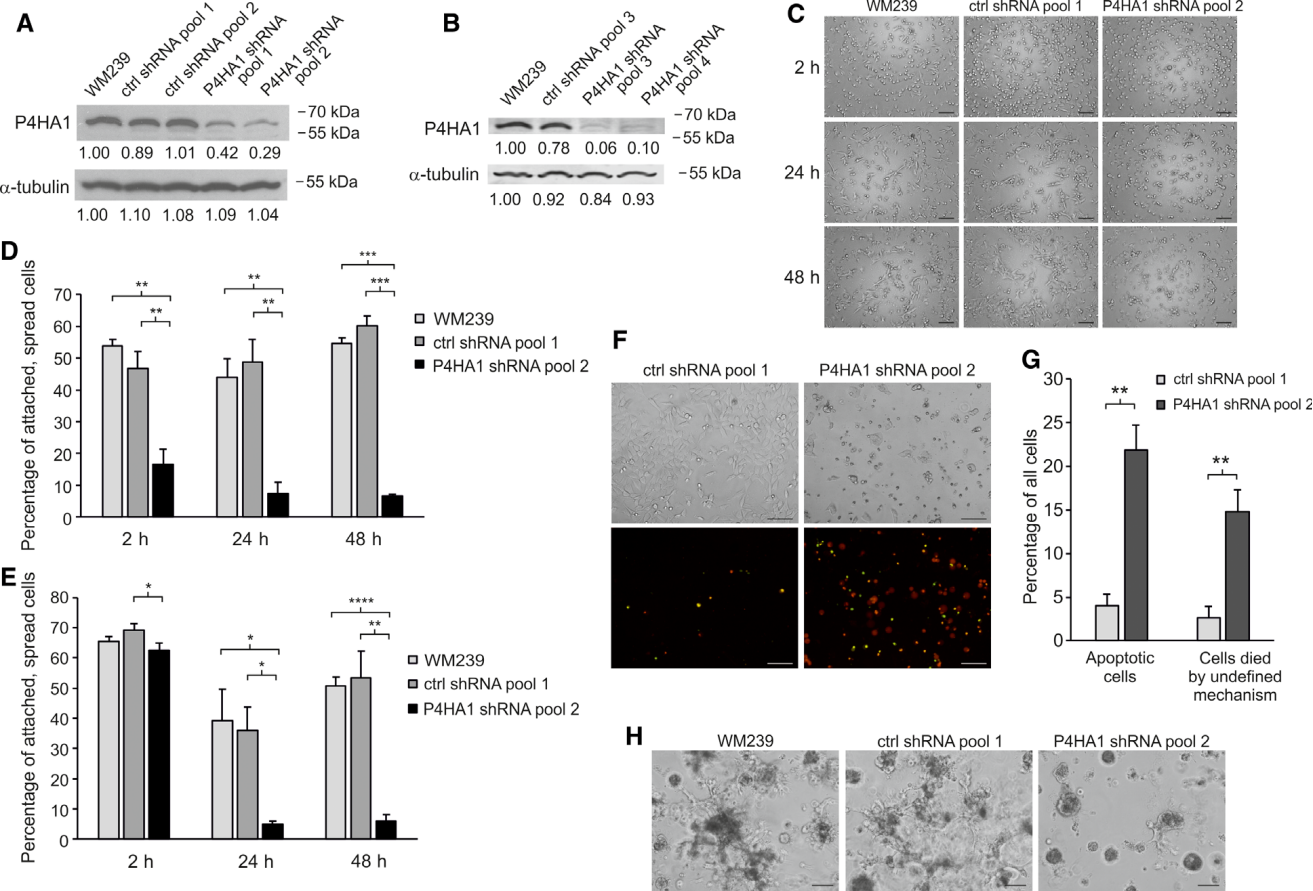
Effect of P4HA1 silencing on cell adhesion, invasion, and apoptosis/viability of WM239 cells in serum‐free media. (A, B) Western blot analysis of P4HA1 protein expression in cell extracts of parental WM239, control (ctrl shRNA), and P4HA1‐KD (P4HA1 shRNA) cells transduced with lentiviral shRNAs from Sigma‐Aldrich/Merck (A) and from Santa Cruz Biotechnology (B). (C) Adhesion of parental WM239, control, and P4HA1‐KD cells plated on uncoated wells in serum‐free media and photographed after 2‐, 24‐, and 48‐h incubation. (D, E) The percentage of attached and spread cells plated on uncoated (D) and collagen‐I‐coated (E) surfaces. (F, G) Effect of P4HA1 silencing on apoptosis and cell death of WM239 cells in serum‐free media. (F) WM239 control and P4HA1‐KD cells were plated in serum‐free media and photographed with phase‐contrast and fluorescence microscopy after 48‐h incubation. Apoptotic cells (stained for activated caspase‐3/7) are seen in green and dead cells (stained with propidium iodide) in red. Apoptotic, dead cells are seen in yellow. (G) The percentage of apoptotic cells (green and yellow) and cells died by undefined mechanism (red) incubated in serum‐free medium for 48 h. Data are expressed as means ± SD of three replicates. *P < 0.05, **P < 0.01, ***P < 0.001, ****P < 0.0001. (H) Effect of P4HA1 silencing on the invasive growth of WM239 cells in Matrigel. Parental WM239, control, and P4HA1‐KD cells were embedded between two layers of Matrigel for 7 days prior to the analysis. Scale bars = 100 µm.

To explore the function of P4HA1 in melanoma, we silenced the expression of P4HA1 in WM239 melanoma cells. In the P4HA1 shRNA cell pools, the P4HA1 protein downregulation varied from 52% to 93% compared to the parental and control shRNA cells as measured by western blot analyses (Fig. 3A,B). P4HA1 protein downregulation was also confirmed by immunofluorescence staining (Fig. S4).

As P4HA1 is needed for collagen secretion and deposition in the ECM, we first studied the effect of P4HA1 downregulation on melanoma cell adhesion in uncoated wells in serum‐free medium. We found that cell adhesion and spreading were significantly reduced in the P4HA1‐knockdown (P4HA1‐KD) cells compared to the parental and control shRNA cells after 2, 24, and 48 h (Fig. 3C,D). Notably, when plated on type I collagen (COL‐I)‐coated wells, both the control and P4HA1‐KD cells initially attached and spread well, but the attachment and spreading were reduced at the later 24‐ and 48‐h time points (Fig. 3E). This suggested that P4HA1 may also be needed for cell survival. Indeed, apoptosis and cell death by undefined mechanisms were found to be significantly increased in the P4HA1‐KD cells compared to the control shRNA cells at the later time points (Fig. 3F,G). We then studied the effect of P4HA1 on melanoma cell invasion in 3D Matrigel and found that the knockdown of P4HA1 markedly reduced the invasive growth of WM239 cells (Fig. 3H).

We have also tried to knock out P4HA1 in WM239 cells using a CRISPR‐Cas9 (D10A mutated double‐nickase)‐mediated system but have not yet obtained complete P4HA1 knockouts. However, we have obtained clones (by cylinder cloning) with 84% to 90% reduction in P4HA1 protein levels (Fig. S5A). It remains to be seen by single cell cloning analyses whether it is a question of heterozygous or mixture of clones, and whether it will even be possible to get a complete knockout. Nevertheless, already the partial knockout clones showed reduced cell adhesion ability compared to the control cells (Fig. S5B–E).

**Figure 4:**
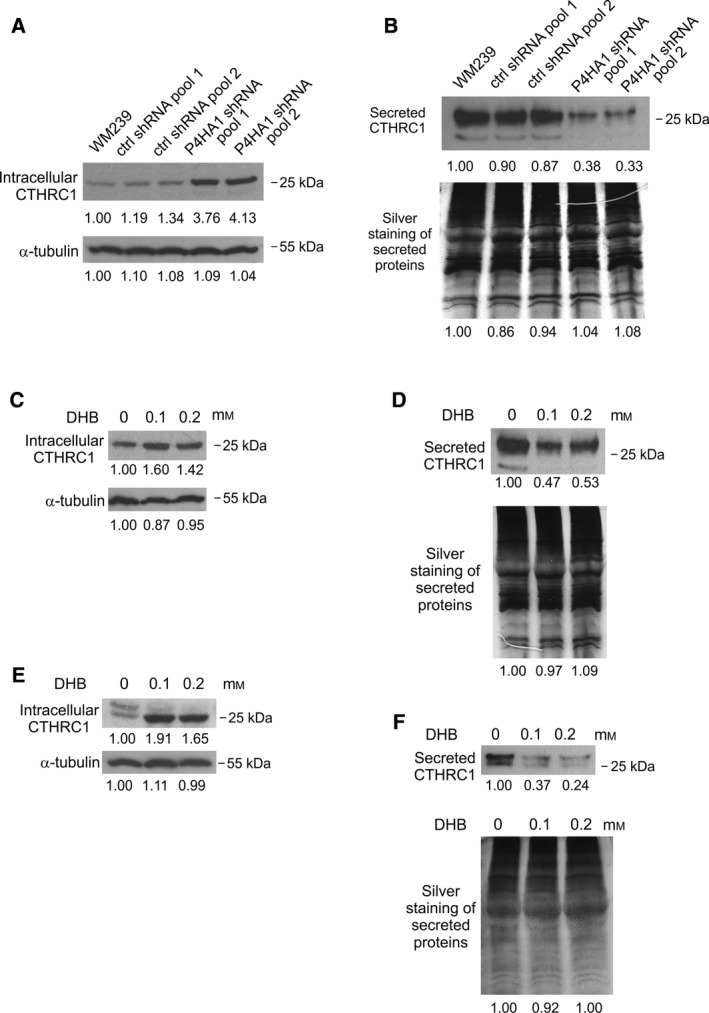
Effect of P4HA1 protein downregulation or treatment with a prolyl 4‐hydroxylase inhibitor on CTHRC1 secretion in WM239 and SKMEL‐28 cells. (A, B) Western blot analyses of CTHRC1 protein expression in cell extracts (A) and in the conditioned medium (B) of parental WM239, control (ctrl shRNA), and P4HA1‐KD (P4HA1 shRNA) cells transduced with lentiviral shRNAs from Sigma‐Aldrich/Merck. (C–F) Western blot analyses of CTHRC1 protein expression in cell extracts of WM239 (C) and SKMEL‐28 (E) cells and in the conditioned medium of WM239 (D) and SKMEL‐28 (F) cells incubated without or with a prolyl 4‐hydroxylase inhibitor 3,4‐dihydroxybenzoic acid (DHB) for 18 h. Alpha‐tubulin was used as a loading control for cell extracts (A, C, E) and silver staining to analyze the total secreted proteins in the conditioned medium (B, D, F). The lower‐molecular‐weight bands of secreted CTHRC1 in the high‐expressing control cells may represent some degradation products of CTHRC1.

Prolyl 4‐hydroxylases catalyze the hydroxylation of proline into 4‐hydroxyproline in the Y position of the GXY triplets (where G is glycine and X is often proline or lysine) within the triple‐helical domains of collagens and other proteins (reviewed in Gorres and Raines, 2010). To find possible targets of P4HA1 in melanoma cell lines, we checked the expression levels of genes encoding collagen domain‐containing proteins in a publicly available microarray data of 62 melanoma cell lines (E‐GEOD‐7127). CTHRC1 showed the highest average expression (30 genes with highest expression are shown in Table S7). CTHRC1 was also highly upregulated in WM239 and WM793 melanoma cell lines compared to normal melanocytes (Table S7). We have previously reported that CTHRC1 is overexpressed in metastatic primary melanomas and plays an important role in melanoma cell migration and invasion (Eriksson *et al.*, 2016). As prolyl 4‐hydroxylation of the collagen triple‐helical domain has been shown to affect the secretion of collagens and complement C1q protein (Muller *et al.*, 1978), we wanted to study whether P4HA1 has an effect on CTHRC1 secretion. Indeed, we found that the knockdown and knockout of P4HA1 in WM239 cells increased (up to 300%) the amount of intracellular CTHRC1 (Fig. 4A and Fig. S6A,C,E) and reduced (up to 85%) the amount of secreted CTHRC1 (Fig. 4B and Fig. S6B,D,F) compared to those of parental and control cells. P4HA1 depletion had no effect on CTHRC1 mRNA levels as quantified by semiquantitative RT–PCR (Fig. S6G) or RNA sequencing. CTHRC1 mRNA expression levels were 4049 ± 1019 in parental and control shRNA WM239 cells (*n* = 4) and 4170 ± 1210 in the P4HA1‐KD cells (*n* = 4).

Next, we inhibited the activity of prolyl 4‐hydroxylases using a competitive inhibitor 3,4‐dihydroxybenzoic acid (DHB). Treatment with DHB increased the amount of intracellular CTHRC1 and reduced CTHRC1 secretion in WM239 (Fig. 4C,D) as well as in SKMEL‐28 melanoma cells (Fig. 4E,F). When analyzed using nonreducing western blotting, it appeared that especially the 25‐kDa monomeric form of CTHRC1 increased in the cell extracts and the high‐molecular‐weight multimers (> 100 kDa) decreased in the secreted proteins of P4HA1‐KD cells and after DHB treatment (Fig. S7).

**Figure 5:**
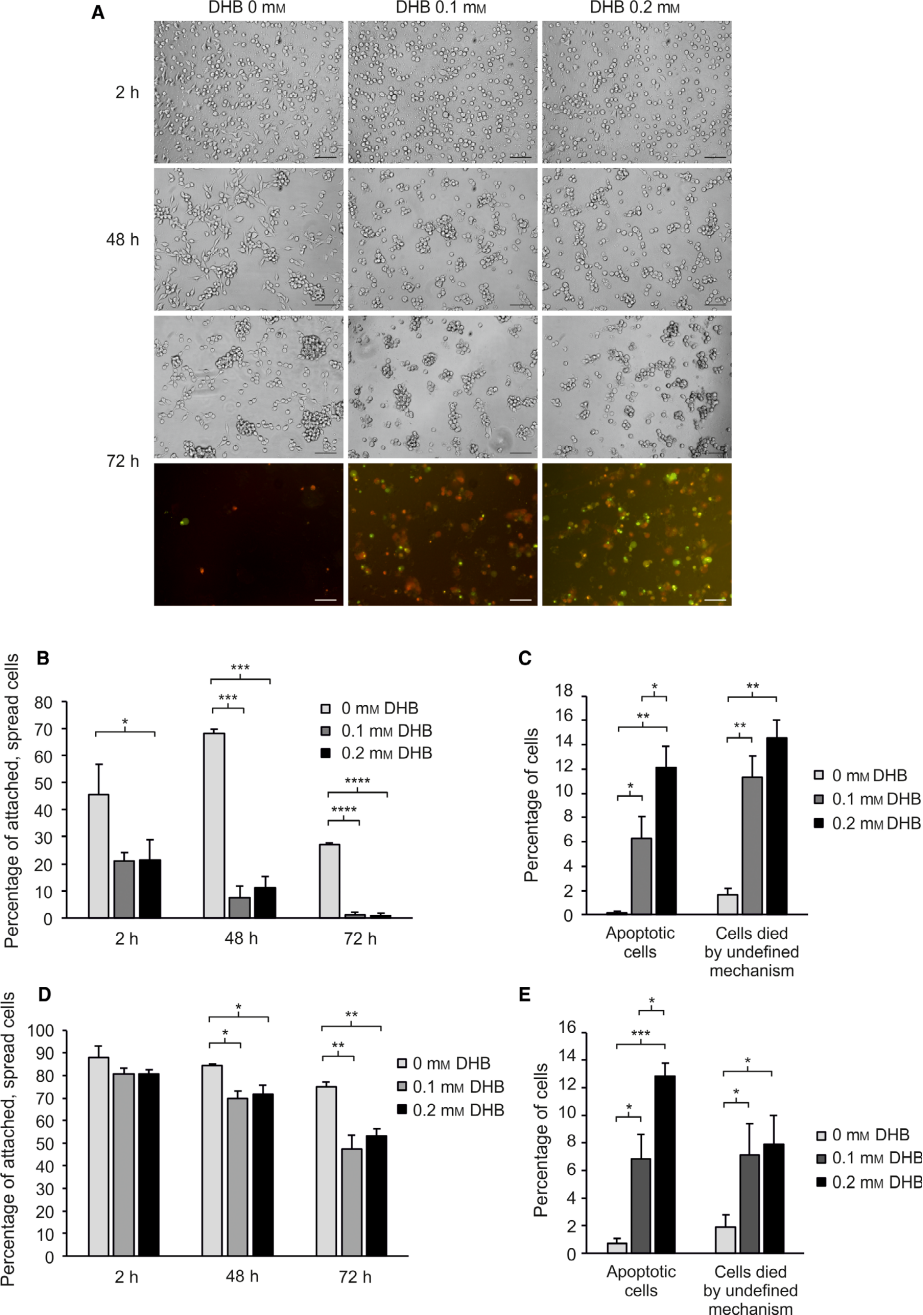
Effect of prolyl 4‐hydroxylase inhibition on cell adhesion and apoptosis/viability of SKMEL‐28 cells plated on uncoated or collagen‐I‐coated surfaces. (A) SKMEL‐28 cells were plated in serum‐free media without or with a prolyl 4‐hydroxylase inhibitor 3,4‐dihydroxybenzoic acid (DHB) and photographed after 2‐, 48‐, and 72‐h incubation. Both phase‐contrast and fluorescence images are shown after 72‐h incubation. Apoptotic cells (with activated caspase‐3/7) are seen in green and dead cells (stained with propidium iodide) in red. Apoptotic, dead cells are seen in yellow. (B–E) The percentage of attached and spread cells (B, D), apoptotic cells (green and yellow) (C, E), and cells died by undefined mechanism (red) (C, E) when incubated in serum‐free medium without or with DHB on uncoated (B, C) or collagen‐I‐coated (D, E) surfaces for 2, 24, and 72 h (B, D), or 72 h (C, E). Data are expressed as means ± SD of three replicates. *P < 0.05, **P < 0.01, ***P < 0.001, ****P < 0.0001. Scale bars = 100 µm.

We further studied the effect of inhibition of prolyl 4‐hydroxylases on SKMEL‐28 cell adhesion and survival in serum‐free medium. DHB significantly reduced cell adhesion (Fig. 5A,B) and increased apoptosis and cell death (by undefined mechanism) on uncoated surfaces (Fig. 5A,C). When plated on COL‐I‐coated wells, no difference in the adhesion between the untreated and DHB‐treated cells could be detected 2 h after plating, and both cells showed increased adhesion compared to the uncoated surfaces (Fig. 5D). However, adhesion of DHB‐treated cells was again reduced after 48 and 72 h compared to the untreated cells, although less markedly than on the uncoated surfaces (Fig. 5B,D). In addition, apoptosis and cell death by undefined mechanism were also significantly increased in the DHB‐treated compared to the untreated cells (Fig. 5E). Similar results were obtained on fibronectin‐coated surfaces (Fig. S8).

When we silenced or knocked out P4HA1 in SKMEL‐28 cells, a 56% to 84% reduction in the P4HA1 protein levels was detected (Fig. S9A,C), but the effect on cell adhesion was only minor. Downregulation of P4HA1 increased the amount of intracellular CTHRC1 (Fig. S9A,C) but unexpectedly did not significantly reduce the amount of secreted CTHRC1 (Fig. S9B,D). As the prolyl 4‐hydroxylase inhibitor, however, showed similar effects (an increase in the intracellular content of CTHRC1 and a clear decrease in secreted CTHRC1) in both SKMEL‐28 and WM239 cell lines (see Fig. 4C–F), we analyzed the expression level of the other common prolyl 4‐hydroxylase α subunit, P4HA2. SKMEL‐28 cells showed markedly higher P4HA2 mRNA expression compared to WM239 cells (Fig. S9E), suggesting that P4HA2 may compensate for the depletion of P4HA1 in P4HA1‐KD and P4HA1‐knockout SKMEL‐28 cells.

### 3.4 P4HA1 expression is associated with hypoxia, glycolysis, and MTORC1 signaling

To reveal which processes P4HA1 may be associated with in melanoma, we studied which genes show correlated mRNA expression to that of P4HA1 in a panel of 62 melanoma cell lines (E‐GEOD‐7127). Many hypoxia‐regulated genes, including MIR210HG, P4HA2, BNIP3, ANGPTL4, VEGFA, PLOD1, and HIF1A, showed positive correlation (genes with highest correlation are shown in Table S8). We subjected the gene list to GSEA to find if these genes may be associated with other pathways and found that in addition to hypoxia these genes were over‐represented in EMT, glycolysis, MTORC1 signaling, and inflammatory response of HALLMARK gene sets (Table S9). We also found that genes that correlated with P4HA1 mRNA expression in primary melanoma tissues (*n* = 103, the Cancer Genome Atlas, TCGA) were over‐represented in hypoxia, glycolysis, and MTORC1 signaling (Table S9).

### 3.5 P4HA1 knockdown affects a small set of invasion‐associated genes

To further elucidate the function of P4HA1 in melanoma, we performed genomewide gene expression analysis of the WM239 P4HA1‐KD cells. RNA sequencing revealed that the knockdown of P4HA1 (downregulated over fivefold compared to the parental and control shRNA cells) resulted in only moderate changes in expression levels of few genes (Table S10). The genes downregulated in P4HA1‐KD cells included, among others, an ECM and basement membrane constituent, laminin α5 (LAMA5), and spire type actin nucleation factor 1 (SPIRE1). The most significantly upregulated genes in P4HA1‐KD cells were, in turn, potassium voltage‐gated channel subfamily E regulatory subunit 4 (KCNE4) and Dickkopf WNT signaling pathway inhibitor 1 (DKK1) (Table S10).

### 3.6 P4HA1 knockdown affects the architecture of WM239 melanoma tumors

**Figure 6:**
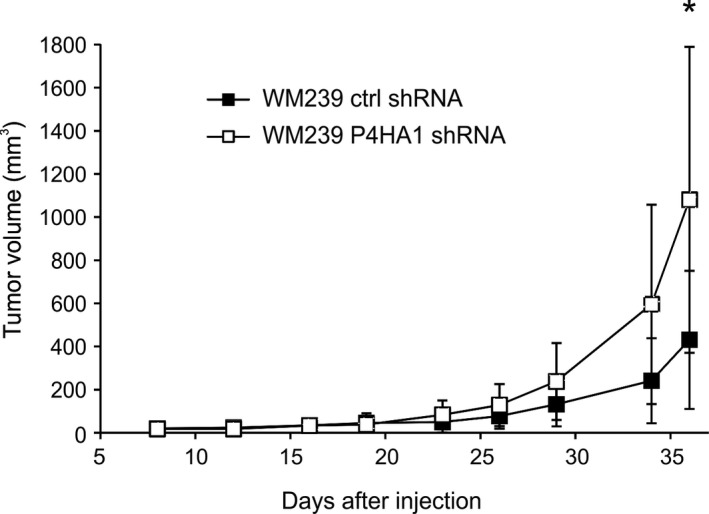
Tumor growth of WM239 control and P4HA1‐knockdown cells in nude mice. WM239 control (ctrl shRNA) and P4HA1‐KD (P4HA1 shRNA) cells (6 × 10^6^) were injected subcutaneously into the lower flank of the mice (nine mice in the control group and eight mice in the P4HA1‐KD group). Tumor volume was measured using a caliper two to three times a week. Bars represent standard deviations. *P < 0.05.

**Figure 7:**
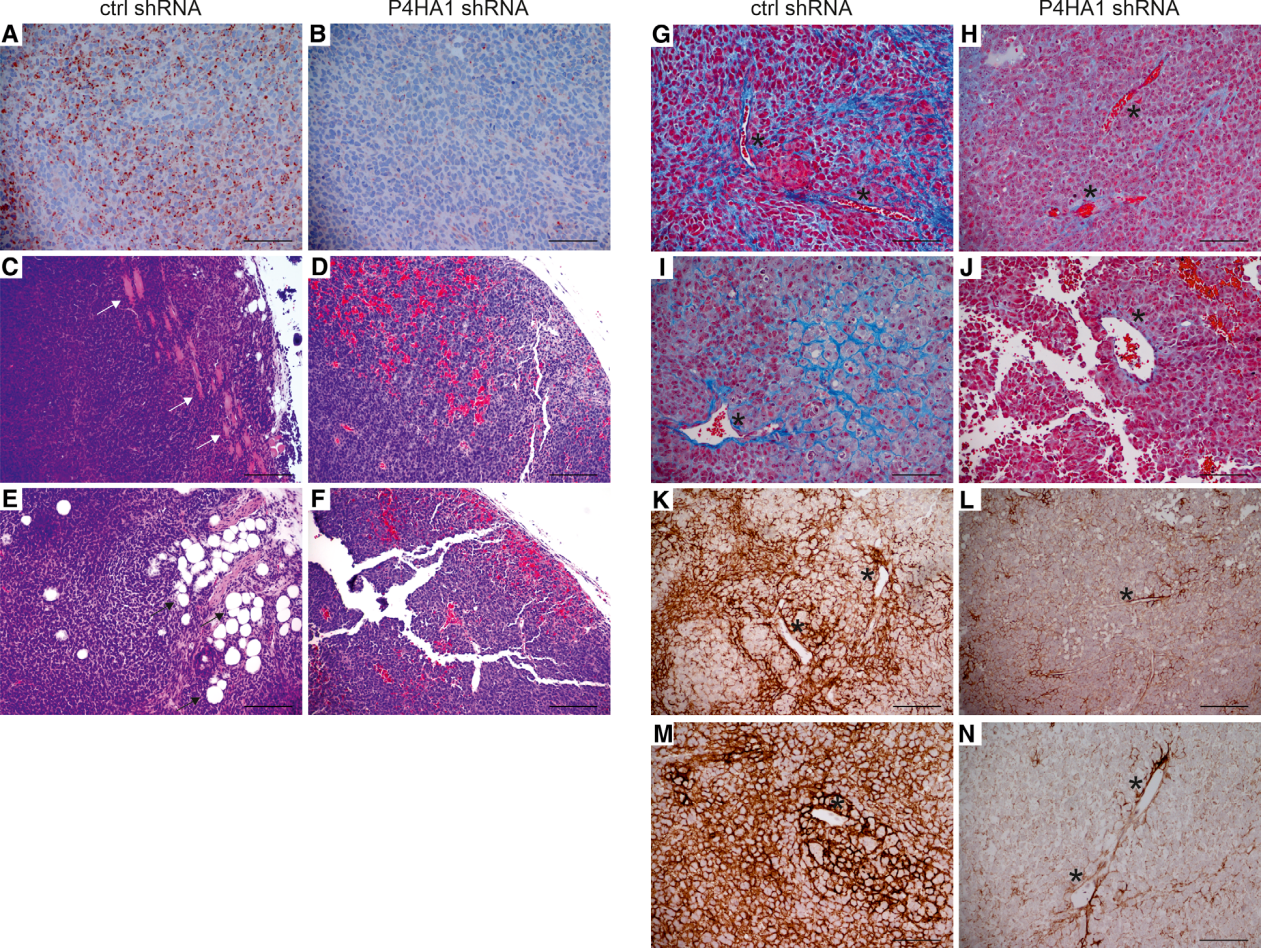
Histochemical analysis of xenograft tumors derived from WM239 control and P4HA1‐knockdown cells. (A, B) Representative images of P4HA1 immunostaining in WM239 control (ctrl shRNA) (A) and P4HA1‐KD (P4HA1 shRNA) (B) tumors. Positive immunostaining is shown in red. (C–F) H&E staining in control (C, E) and P4HA1‐KD (D, F) tumors. Muscle is stained light pink and red blood cells red. Invasion to muscle is marked with white arrows and to fat tissue with black arrows. (G–J) Masson’s trichrome staining in control (G, I) and P4HA1‐KD (H, J) tumors. Collagen fibers are stained blue and red blood cells red. (K–N) Immunostaining of COL‐IV in control (K, M) and P4HA1‐KD (L, N) tumors. Positive immunostaining is shown in brown. (G–N) Examples of blood vessels are marked with asterisks. Scale bars = 100 µm (A, B, G–J, M, N), 200 µm (C–F, K, L). Note that the P4HA1‐KD tumor sections are more prone to tearing due to the loosened tissue structure.

To study the effect of P4HA1 on *in vivo* tumorigenesis, we injected the WM239 P4HA1‐KD and control shRNA cells (6 × 10^6^) subcutaneously into the lower flanks of nude mice. As P4HA1 expression correlated with poor patient survival in human primary melanoma samples, we unexpectedly observed that P4HA1 knockdown increased tumor size in nude mice (Fig. 6). When the tumors were excised at day 36, the P4HA1‐KD tumors were, however, found to be markedly less compact than the control tumors. We first confirmed the downregulation of P4HA1 in the knockdown tumors by immunohistochemical staining (Fig. 7A,B). In the H&E staining, the P4HA1‐KD tumors showed increased hemorrhage, large necrotic areas, and altogether a more loosened tissue architecture than the control tumors (Fig. 7C–F and Fig. S10A–D). In addition, although the control tumors were smaller, we found extensive invasion through the cutaneous striated muscle layer, panniculus carnosus (see also Nummela *et al.*, 2012), toward the epidermis and/or fat tissue invasion (Fig. 7C,E and Fig. S10E–I) in six of the nine control tumors and only in two of the eight P4HA1‐KD tumors. As the prolyl 4‐hydroxylase is a key enzyme in collagen synthesis and deposition, we stained the tumors with Masson’s trichrome to see whether P4HA1 knockdown affects the fibrillar collagen content of the tumors. In the control tumors, we found abundant collagen fibers surrounding tumor cells/cell nests and intratumoral blood vessels (Fig. 7G,I). The P4HA1‐KD tumors showed markedly weaker collagen staining, indicating reduced collagen deposition, especially around blood vessels (Fig. 7H,J and Fig. S10J,K). The vessel walls were also frequently ruptured in the P4HA1‐KD tumors, showing leakage of red blood cells (Fig. 7J and Fig. S10J,K). As the P4HA1‐KD tumors appeared to be highly hemorrhagic, we further studied if there are changes in the amount of type IV collagen (COL‐IV) in the basal lamina of the tumor blood vessels. Many blood vessels in the P4HA1‐KD tumors showed reduced and fragmentary COL‐IV deposition compared to the control tumors (Fig. 7K–N). The overall assembly of COL‐IV networks around the tumor cells was also severely reduced in P4HA1‐KD tumors, as compared to the control tumors showing strong COL‐IV staining around tumor cells and cell nests (Fig. 7K–N). No difference in the mRNA levels of the major COL‐IV chains was detected between the control and P4HA1‐KD cells (COL4A1 and COL4A2 mRNA expression levels were 11 688 ± 571 and 18 409 ± 942 in parental WM239 and control shRNA cells, respectively, and 10 990 ± 423 and 17 465 ± 96 in P4HA1‐KD cells, respectively). We further specifically studied the major fibrillar collagen COL‐I and found that its deposition was also reduced in the P4HA1‐KD tumors compared to the controls (Fig. S11A–E). The control tumors showed more abundant, although heterogeneous staining of COL‐I around tumor cells (Fig. S11A) and high COL‐I expression around the tumor blood vessels (Fig. S11C). As we found P4HA1 knockdown to reduce the secretion of CTHRC1 in melanoma cells *in vitro*, and our previous results have shown that CTHRC1 is localized in the blood vessels in human melanomas (Eriksson *et al.*, 2016), we further studied the expression of CTHRC1 in the xenograft tumors (Fig. S11F–J). The control tumors showed diffuse CTHRC1 staining in tumor cells and a more prominent staining around tumor blood vessels (Fig. S11F,H), while the P4HA1‐KD tumors showed reduced CTHRC1 staining especially around tumor blood vessels (Fig. S11G,I). The blood vessel density (visualized by staining of the endothelial cell marker CD31) did not appear to markedly differ between the control and P4HA1‐KD tumors (Fig. S12). However, the CD31 staining revealed frequent breakages of the endothelial cell layer in the blood vessels of the P4HA1‐KD tumors (Fig. S12H).

To unravel the reasons for the larger tumor size in P4HA1‐KD tumors, we also stained the tumors for the cell proliferation marker Ki‐67. Both the control and P4HA1‐KD tumors consisted of highly proliferative cells and showed no difference in their Ki‐67 staining patterns (Fig. S13). Consistent with a recent publication (Miller *et al.*, 2018), the Ki‐67 staining of the nuclei was graded rather than binary (see Fig. S13, higher magnifications). The Ki‐67 labeling indices varied from 70% to 90% in different regions of both the control and P4HA1‐KD tumors, apparently depending on the growth rates and cell cycle phases of the heterogeneous tumor cells (Miller *et al.*, 2018). The proliferation rates of the cells did not differ either *in vitro*. As the P4HA1‐KD cells showed increased apoptosis under serum‐free conditions *in vitro*, we further stained the tumors for cleaved caspase 3 (early apoptosis marker) to see whether the silencing of P4HA1 also increases apoptosis *in vivo*. Seven of nine control and two of eight P4HA1‐KD tumors contained only single isolated cells positive for the cleaved caspase 3 (Fig. S14A,B). The remaining two control tumors showed small clusters (Fig. S14C–D) and the six P4HA1‐KD tumors larger clusters of apoptotic cells (Fig. S14E–J). The number of cleaved caspase 3‐positive cells per tumor mm^2^ (calculated from the whole tumor sections) was significantly increased in the P4HA1‐KD tumors (Fig. S14K, fold 2.2, *P* = 0.008). We further analyzed the tumors for late‐stage apoptosis (and necrosis) by TUNEL labeling. The P4HA1‐KD xenografts showed significantly higher number of TUNEL‐positive cells compared to the control tumors (Fig. S15, fold 4.1, *P* = 0.019).

## 4 Discussion

Melanoma, one of the most aggressive cancers, is a highly unpredictable disease and continues to be very difficult to cure. In this study, we aimed to find new prognostic markers and therapeutic targets based on the molecular pathways activated or deactivated in aggressive melanomas. The identified short survival genes were enriched especially in the EMT, ECM organization, collagen formation, and angiogenesis, all linked to the metastatic progression of tumors (Erler and Weaver, 2009). RHOC, one of the potential short survival marker genes, encodes a small GTPase, which regulates the reorganization of the actin cytoskeleton and promotes EMT. RHOC expression has been found to associate with shorter disease‐free and overall survival in melanoma patients, but not as an independent predictor (Boone *et al.*, 2009). RHOC may also serve as a target for the cytotoxic T cells in patients with metastatic cancer (Wenandy *et al.*, 2008), making it an interesting possible target for immunotherapy in melanoma. Also, the downregulation of one of our long survival marker genes, CXADR, encoding a tight junction protein, may enhance tumor progression by promoting EMT. Downregulation of CXADR appears to sensitize breast cancer cells to TGFβ‐induced EMT, and its low expression correlates with poor prognosis in luminal A breast cancers (Nilchian *et al.*, 2019).

Of our topmost novel prognostic marker genes for primary melanomas, P4HA1 seemed the most interesting as its expression was largely tumor cell‐specific and upregulated in melanoma cell lines compared to normal melanocytes. Its mRNA expression is also reported to associate with poor survival in breast cancer (Gilkes *et al.*, 2013b) and squamous cell carcinoma (Kappler *et al.*, 2017; Tawk *et al.*, 2016), and the P4HA1 protein expression correlates with poor prognosis in high‐grade gliomas (Hu *et al.*, 2017). Our preliminary results from immunohistochemical staining of primary melanomas suggest that, in addition to P4HA1 mRNA levels, also high P4HA1 protein expression may be associated with increased metastasis and mortality and may serve as a prognostic factor (data not shown). In our gene set enrichment analysis, we found that P4HA1 is a part of a gene expression signature associated with hypoxia and glycolysis both in melanoma cell lines and in clinical melanoma primary tumors. Hypoxia is observed in most solid tumors and has been associated with poor patient outcome (reviewed in Vaupel and Mayer, 2007). The expression of hypoxia‐induced genes or gene expression signatures (reviewed in Harris *et al.*, 2015) has been studied as surrogate markers for tumor hypoxia. Interestingly, Tawk *et al. *(2016) have tested three previously developed hypoxia gene signatures as prognostic factors in head and neck squamous cell carcinoma and found that the signature genes could be reduced to P4HA1, the only gene common in all signatures. P4HA1 expression is induced in hypoxia by the hypoxia‐inducible factor 1 (HIF‐1) (Gilkes *et al.*, 2013a), which regulates several pathways involved in tumor progression (reviewed in Balamurugan, 2016), such as EMT, angiogenesis, invasion, inflammation, and tumor metabolism, including the glycolytic pathway. P4HA1 expression may also be regulated by hypoxia‐independent factors, including TGFβ and USF1/USF2 transcription factors (Chen *et al.*, 2006). Further, other hypoxia‐independent drivers, such as the oncogenic BRAFV600E mutation, have been found to increase HIF‐1α protein (the oxygen‐regulated subunit of HIF‐1) and the expression of HIF‐1‐regulated genes in melanoma (reviewed in Meierjohann, 2015). Recently, P4HA1 has also been found to stabilize HIF‐1α independently of oxygen levels (Xiong *et al.*, 2018), leading to a positive feedback loop and increased expression of HIF‐1‐induced genes. These results suggest an important role for P4HA1 in HIF‐1 signaling pathway and the potential of P4HA1 as a prognostic marker.

In our analyses of primary melanoma tumors, both the tumor cells and the stromal fibroblasts produced P4HA1, although the tumor cells appeared to be the main producers. Also in other cancers, such as gliomas and prostate cancer, P4HA1 staining has mostly been reported in the tumor cells (Chakravarthi *et al.*, 2014; Zhou *et al.*, 2017). This is interesting, as fibroblasts are considered the main producers of collagens, which are the most important substrates for P4HA1. It seems, that at least in melanoma, the tumor cells may be responsible for the secretion of many collagens into their microenvironment. This was also evident in our xenograft model, where knockdown of P4HA1 in the melanoma cells led to reduced collagen deposition in the tumor tissues. As prolyl 4‐hydroxylase is an intracellular enzyme and it affects the hydroxylation of newly synthesized procollagen chains, the majority of secreted collagen in the xenograft tumors seems to be produced by the melanoma cells and not the host stromal cells. As COL‐IV staining was also reduced in the tumor blood vessels, it appears that melanoma cell‐secreted COL‐IV also contributes to the formation of basal lamina of the tumor blood vessels and thereby to the integrity of the vessels.

Knockdown of P4HA1 has been reported to inhibit cancer cell proliferation *in vitro* and reduce tumor growth in xenograft models of breast and prostate cancer and gliomas (Chakravarthi *et al.*, 2014; Gilkes *et al.*, 2013b; Zhou *et al.*, 2017). In our melanoma model, the silencing of P4HA1 did not appear to interfere with tumor cell proliferation *in vitro* or *in vivo*, as assessed by staining with the cell proliferation marker Ki‐67*.* The P4HA1‐KD tumors were, however, larger than the control tumors, but their tissue structure was very loose, likely due to the markedly reduced COL‐IV network assembly and COL‐I deposition in the ECM. P4HA1 knockdown has been shown to reduce tumor density and stiffness also in breast cancer xenografts (Gilkes *et al.*, 2013b). Furthermore, our P4HA1‐knockdown tumors were highly hemorrhagic, which could lead to the accumulation of fluid, partly explaining the larger volume of the tumors. Hemorrhages appeared to be caused by rupture of tumor blood vessels, weakened by the lack of COL‐IV (normally providing structural support) in the basement membranes and in the surrounding tumor tissue. The knockdown of P4HA1 has also been found to inhibit the synthesis of COL‐IV and disrupt the structures of vascular basement membranes in glioma xenografts (Zhou *et al.*, 2017). Further, knockout of *P4ha1* in mice is embryonic lethal, most likely due to deficient assembly of COL‐IV (Holster *et al.*, 2007). Our results also show that the expression of laminin α5 was downregulated in P4HA1‐knockdown cells, which may further weaken the vascular basement membranes.

We have previously found that CTHRC1 is an important mediator of melanoma cell migration and invasion *in vitro*, and that in clinical melanoma tumor samples, CTHRC1 is expressed in melanoma cells, tumor‐associated fibroblasts, and tumor blood vessels (Eriksson *et al.*, 2016). Here, we show that P4HA1 knockdown reduced CTHRC1 secretion in melanoma cells *in vitro* and CTHRC1 protein deposition around tumor blood vessels *in vivo*. Interestingly, overexpression of CTHRC1 in endothelial cells and addition of the recombinant CTHRC1 have been found to promote endothelial cell migration and tubulogenesis *in vitro* (Fu *et al.*, 2017; Lee *et al.*, 2016). Further, CTHRC1 expression has been reported to correlate with increased blood vessel density in mouse pancreatic tumor xenografts (Lee *et al.*, 2016) and in human gastrointestinal stromal tumors (Fu *et al.*, 2017). Altogether, these data suggest an important role for CTHRC1 in P4HA1‐regulated neovascularization.

We further found that P4HA1 depletion inhibited the invasion of melanoma cells *in vitro* and *in vivo*. This has also been described for breast cancer xenograft tumors, where P4HA1 silencing inhibits tumor invasion and formation of lung and lymph node metastases (Gilkes *et al.*, 2013b). P4HA1 may promote tumor metastasis by enhancing the secretion of collagens, which form together with other extracellular molecules, such as fibronectin and periostin, fibrillar networks that regulate cell motility and invasion (Soikkeli *et al.*, 2010). Also, laminin α5, which was downregulated in the P4HA1‐knockdown cells, strongly promotes melanoma cell migration (Oikawa *et al.*, 2011). P4HA1 may also enhance invasion by regulating the expression of invasion‐associated genes, such as SPIRE1, which was downregulated, and DKK1, which was upregulated in the P4HA1‐knockdown cells. SPIRE1 is an actin nucleator, which is a part of the invadosome and promotes matrix degradation (Lagal *et al.*, 2014). DKK1, in turn, is a secreted inhibitor of Wnt signaling (Fedi *et al.*, 1999), and its overexpression decreases the invasive capability of melanoma cells *in vitro* (Chen *et al.*, 2012) and increases apoptosis in melanoma cells *in vitro* and *in vivo* (Mikheev *et al.*, 2007). How P4HA1 may regulate the expression of these genes remains to be clarified, but one mechanism by which P4HA1 may affect gene expression is through stabilization of HIF‐1α (Xiong *et al.*, 2018).

We showed that both knockdown of P4HA1 expression and inhibition of the prolyl 4‐hydroxylase activity reduced melanoma cell adhesion on uncoated surfaces in serum‐free medium, suggesting that one function of P4HA1 in melanoma cells is to promote collagen production for their own adhesion and spreading. In accordance with this, the cells were able to adhere well to exogenous COL‐I and fibronectin early after seeding. Cell adhesion and viability were, however, reduced after longer periods of incubation, suggesting that inhibition of prolyl 4‐hydroxylase reduces some additional attachment or survival factors, resulting in apoptosis/anoikis. The silencing of P4HA1 also increased apoptosis and cell death *in vivo*, but only in regions where the microenvironmental changes may have caused cellular stress, for example as a result of hemorrhages and hypoxia/anoxia.

Although the prolyl 4‐hydroxylase inhibitor DHB showed a similar effect in both WM239 and SKMEL‐28 cell lines, P4HA1 silencing only resulted in minor changes in the cellular behavior of the SKMEL‐28 cells. This may be due to a compensatory effect of the other common prolyl 4‐hydroxylase alpha subunit, P4HA2, which was expressed at a higher level in SKMEL‐28 cells than in WM239 cells, although previous studies have suggested that P4HA1 may largely compensate for the lack of P4HA2 but not *vice versa* (Aro *et al.*, 2015; Holster *et al.*, 2007). Further, in primary melanoma tumor samples (from http://www.ncbi.nlm.nih.gov/geo/query/acc.cgi?acc=GSE98349 dataset), P4HA2 was expressed at a lower level than P4HA1, and its mRNA expression was not significantly associated with survival (as analyzed by us), in concert with our data. In another study, high P4HA2 mRNA levels were reported to correlate with poor prognosis in a dataset comprised mostly of melanoma metastases (Atkinson *et al.*, 2019). Of note, also high P4HA1 mRNA levels were significantly associated with shorter disease‐specific survival in that dataset using the same cutoff parameters, although that was not reported in the study. In breast cancer, high P4HA1 and P4HA2 mRNA levels in primary tumors associate with poorer overall survival, and knockdown of either P4HA1 or P4HA2 may reduce collagen deposition and inhibit tumor invasion and metastasis *in vivo* (Gilkes *et al.*, 2013b). These data suggest that depending on the cell type and the relative expression levels of P4HA1 and P4HA2, inhibition of both prolyl 4‐hydroxylase alpha subunits may be needed to effectively block the prolyl 4‐hydroxylase function. Systemic treatment with a prolyl 4‐hydroxylase inhibitor, ethyl 3,4‐dihydroxybenzoate (EDHB), has been reported to decrease tumor fibrosis and metastasis in a breast tumor xenograft model (Gilkes *et al.*, 2013b). However, the tumors were derived from P4HA1‐overexpressing and HIF‐1α‐ and HIF‐2α‐silenced MDA‐MB‐231 cells (as EDHB may stabilize HIF‐1 and HIF‐2 levels through inhibition of HIF prolyl 4‐hydroxylases), complicating the interpretations. Many compounds that inhibit collagen prolyl 4‐hydroxylases have been developed but suffer from poor selectivity (they also inhibit HIF prolyl 4‐hydroxylases) or toxic off‐target effects (reviewed in Vasta and Raines, 2018). Altogether, therapeutic targeting of P4HA1 (and P4HA2) is an interesting possibility for the treatment of aggressive melanomas, and other cancers, but more specific inhibitors, are still needed.

## 5 Conclusions

In summary, we identified several novel molecules associated with melanoma progression that could predict patient survival in primary melanomas, independent of the Breslow’s tumor thickness. Many of the identified potential prognostic marker genes were associated with processes linked to the metastatic progression of tumors, including EMT, ECM organization, collagen formation, and angiogenesis. We studied further the significance of one of these genes, P4HA1, in melanoma progression and show that P4HA1 depletion in melanoma cells reduced cell adhesion, invasion, and viability *in vitro*. Our melanoma xenograft model suggested that P4HA1 knockdown also reduces melanoma invasion *in vivo* as well as the deposition of collagens, particularly COL‐IV, in the interstitial ECM and in the basement membranes of tumor blood vessels. This compromises the overall tissue integrity and leads to rupture of blood vessel walls and hemorrhages. Besides providing structural support, collagens also give signaling cues to cells and may regulate cell proliferation, apoptosis, angiogenesis, invasion, and metastasis (Fidler *et al.*, 2018; Nissen *et al.*, 2019; Wu and Ge, 2019). Further, our results show that P4HA1 knockdown reduced the secretion of CTHRC1, an important mediator of melanoma cell migration and invasion, *in vitro* and its deposition around tumor blood vessels *in vivo*, suggesting that this angiogenesis‐promoting molecule may also play an important role in P4HA1‐regulated neovascularization. Taken together, P4HA1 is a highly interesting potential prognostic marker and therapeutic target, influencing many aspects of melanoma tumor progression.

## Conflict of interest

The authors declare no conflict of interest.

## Author contributions

JE and EH conceived and designed the study, interpreted the data, and wrote the manuscript. JE performed most experiments and drafted the manuscript. VLJ and PL designed and carried out the xenograft experiments and edited the manuscript. VLJ performed the TUNEL assay, and VLJ and JE quantified the tumor stainings. TJ, SV, and OS provided/analyzed the clinical melanoma samples.

## Supporting information


**Fig S1.** Comparison of the staining patterns of P4HA1 antibodies in immunohistochemical analyses.

**Fig. S2.** Prognostic value of selected potential marker genes of short survival in primary melanomas.

**Fig. S3.** Prognostic value of selected potential marker genes of long survival in primary melanomas.

**Fig. S4.** Knockdown of P4HA1 protein in WM239 cells.

**Fig. S5.** Effect of P4HA1 depletion on adhesion of WM239 cells in serum‐free media.

**Fig. S6.** Effect of P4HA1 protein downregulation on CTHRC1 secretion in WM239 cells.

**Fig. S7.** Effect of P4HA1 protein downregulation or prolyl 4‐hydroxylase inhibition on CTHRC1 secretion.

**Fig. S8.** Effect of prolyl 4‐hydroxylase inhibition on cell adhesion and apoptosis/viability of SKMEL‐28 cells plated on fibronectin‐coated surfaces.

**Fig. S9.** Effect of P4HA1 protein downregulation on CTHRC1 secretion in SKMEL‐28 cells.

**Fig. S10.** Histochemical analysis of xenograft tumors derived from WM239 control and P4HA1‐knockdown cells.

**Fig. S11.** Immunohistochemical staining of COL‐I and CTHRC1 in frozen sections of xenograft tumors derived from WM239 control and P4HA1‐knockdown cells.

**Fig. S12.** Immunohistochemical staining of the endothelial cell marker CD31 in xenograft tumors derived from WM239 control and P4HA1‐knockdown cells.

**Fig. S13.** Immunohistochemical staining of the cell proliferation marker Ki‐67 in xenograft tumors derived from WM239 control and P4HA1‐knockdown cells.

**Fig. S14.** Immunohistochemical staining of the apoptosis marker cleaved caspase 3 in xenograft tumors derived from WM239 control and P4HA1‐knockdown cells.

**Fig. S15.** Analysis of apoptosis (and necrosis) by TUNEL staining in xenograft tumors derived from WM239 control and P4HA1‐knockdown cells.

**Table S1.** PCR variables.

**Table S2.** Significance Analysis of Microarrays (SAM) results of mRNA expression levels in primary melanomas associated most significantly with patient survival (higher expression in cases with short survival).

**Table S3.** Significance Analysis of Microarrays (SAM) results of mRNA expression levels in primary melanomas associated most significantly with patient survival (lower expression in cases with short survival).

**Table S4.** Gene Set Enrichment Analysis results for genes associated most significantly with patient survival.

**Table S5.** Kaplan‐Meier survival analysis and mean survival times of patients with primary melanomas that show low and high mRNA expression of the top short survival marker genes in an independent RNA sequencing data set (GSE98394).

**Table S6.** Kaplan‐Meier survival analysis and mean survival times of patients with primary melanomas that show low and high mRNA expression of the top long survival marker genes in an independent RNA sequencing data set (GSE98394).

**Table S7.** Expression levels of genes encoding collagen domain‐containing proteins in 62 melanoma cell lines (E‐GEOD‐7127).

**Table S8.** Genes correlating with P4HA1 expression in a panel of 62 melanoma cell lines (E‐GEOD‐7127).

**Table S9.** Gene Set Enrichment Analysis results for genes that correlate with P4HA1 expression in melanoma cell lines and primary melanoma tissues.

**Table S10.** Gene expression changes in WM239 cells after knockdown of P4HA1 expression.
