# Supplementary figures and images for: Prolyl 4‐hydroxylase subunit alpha 1 (P4HA1) is a biomarker of poor prognosis in primary melanomas, and its depletion inhibits melanoma cell invasion and disrupts tumor blood vessel walls
Source: Mol Oncol. 2020 Feb 28;14(4):742–62. doi: 10.1002/1878-0261.12649 (PMC7138405; doi:10.1002/1878-0261.12649)

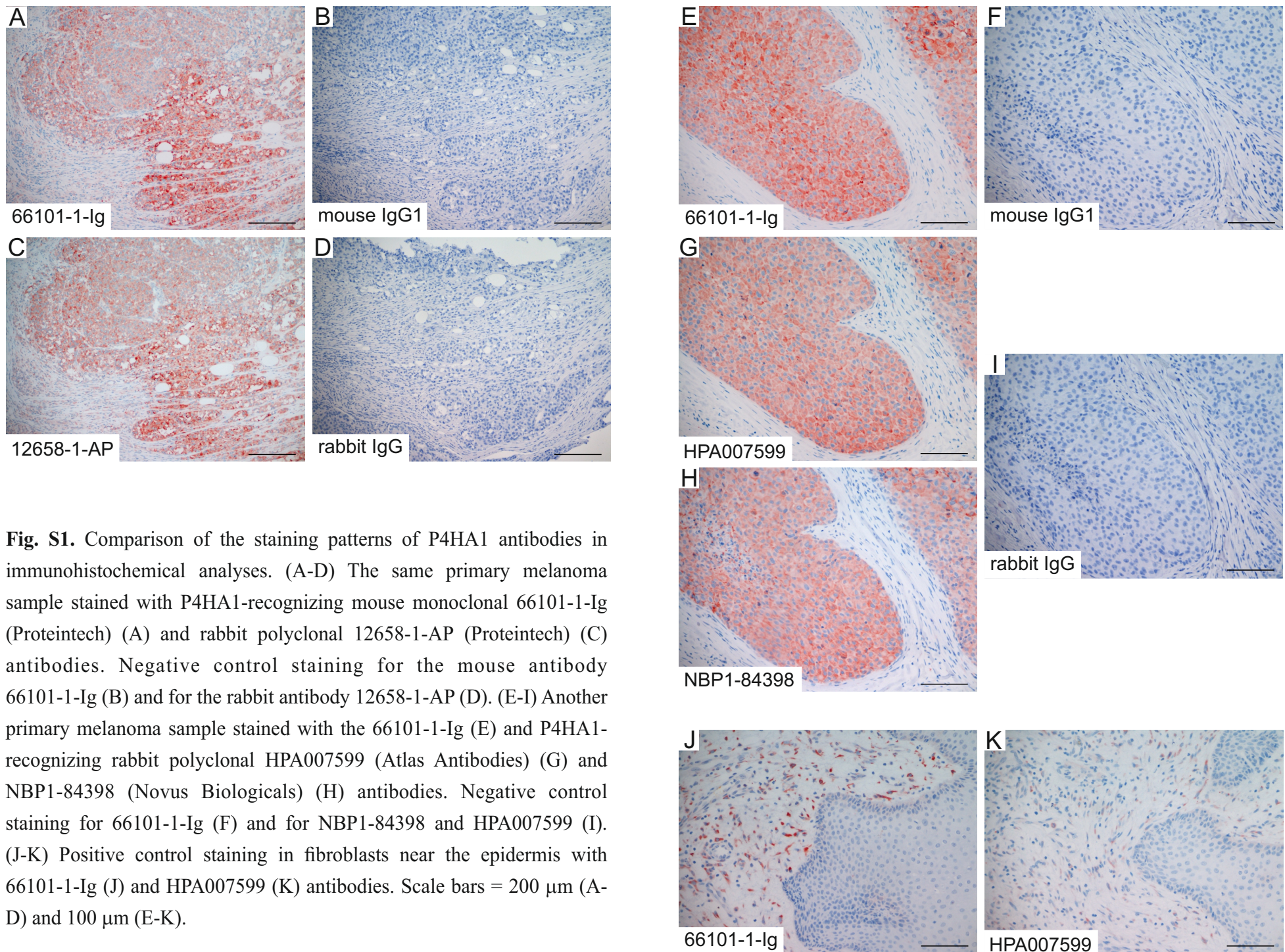

Supplement: Supplementary file 1 — Fig S1. Comparison of the staining patterns of P4HA1 antibodies in immunohistochemical analyses. [file MOL2-14-742-s001.pdf]

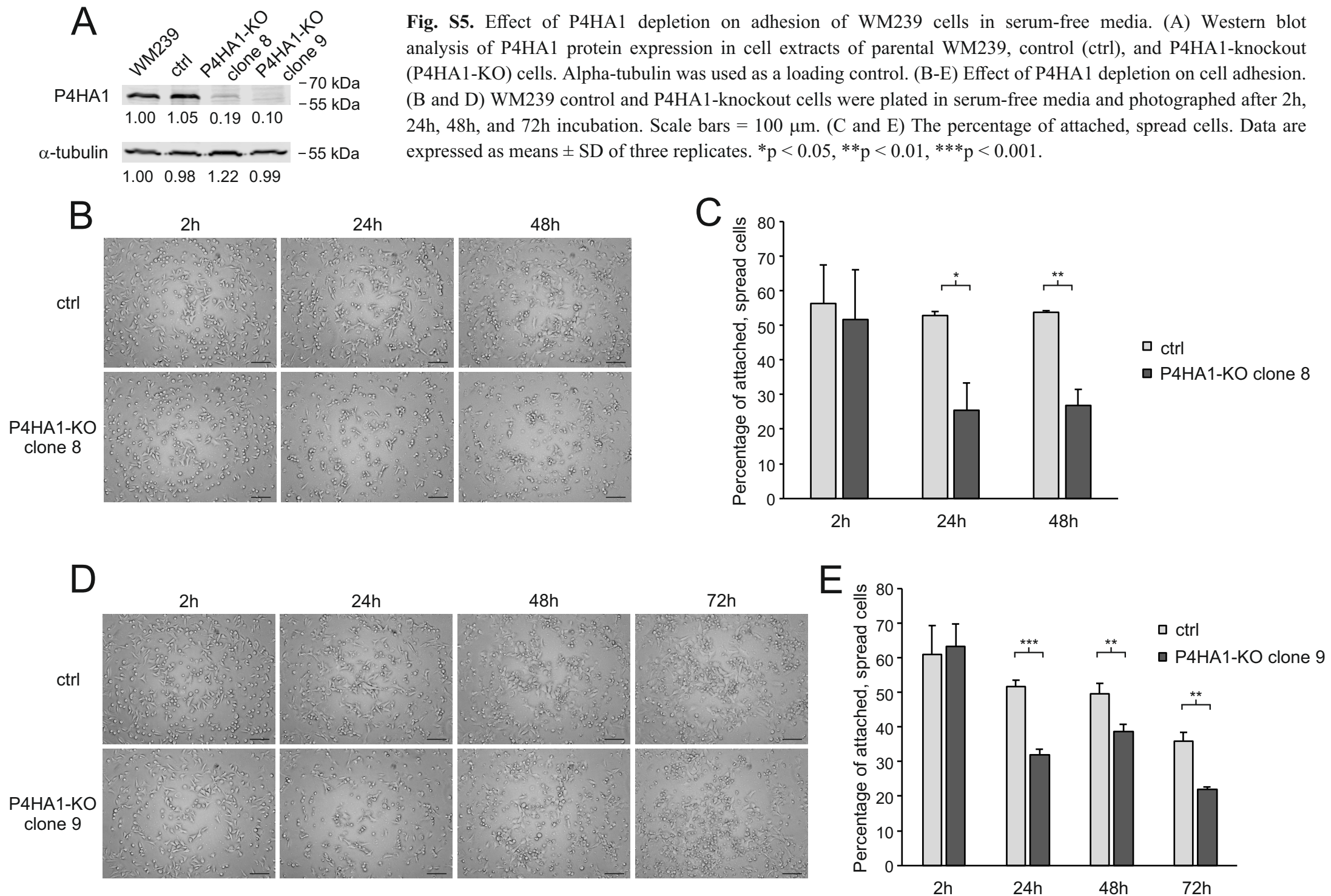

Supplement: Supplementary file 5 — Fig. S5. Effect of P4HA1 depletion on adhesion of WM239 cells in serum‐free media. [file MOL2-14-742-s005.pdf]

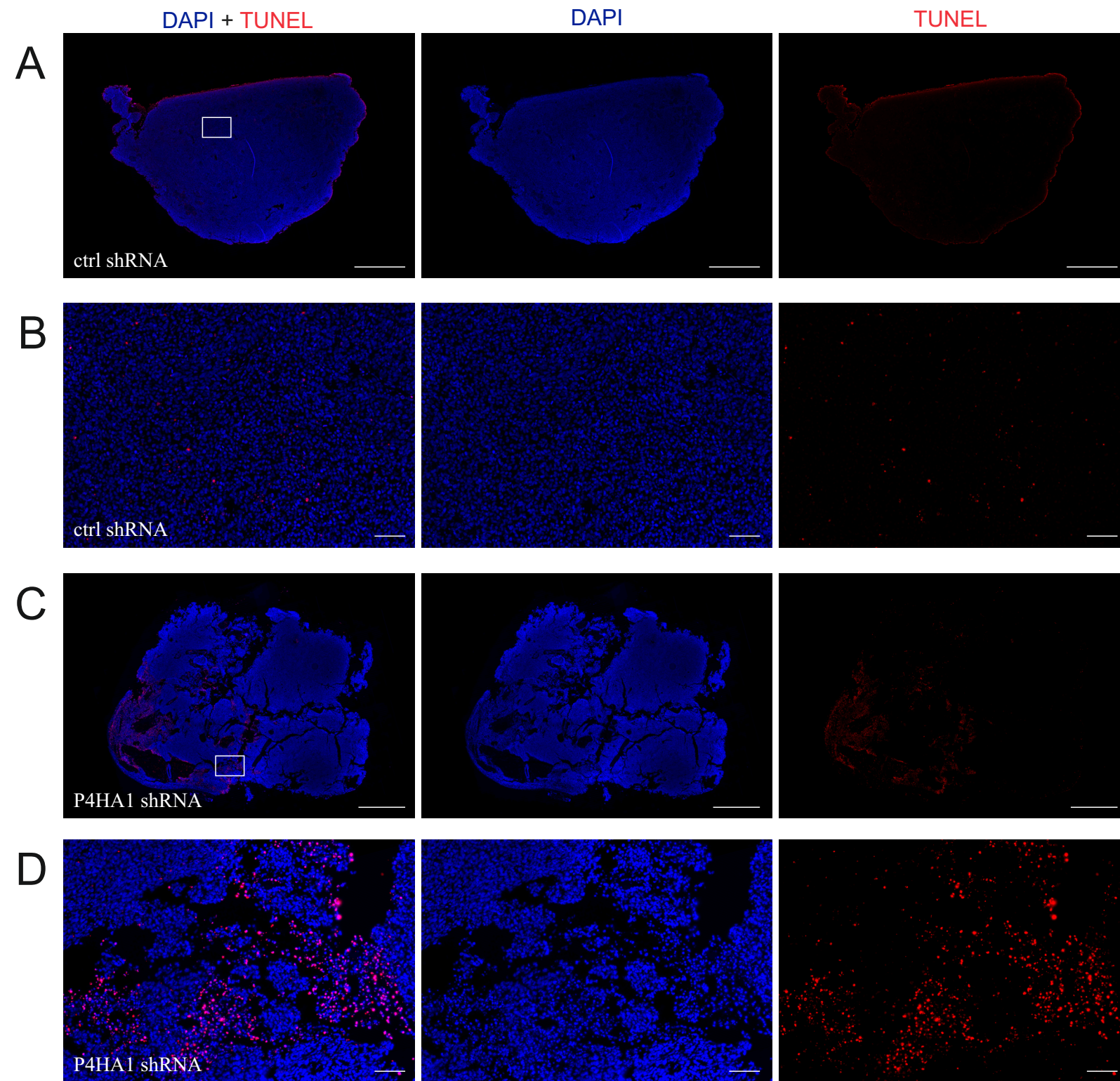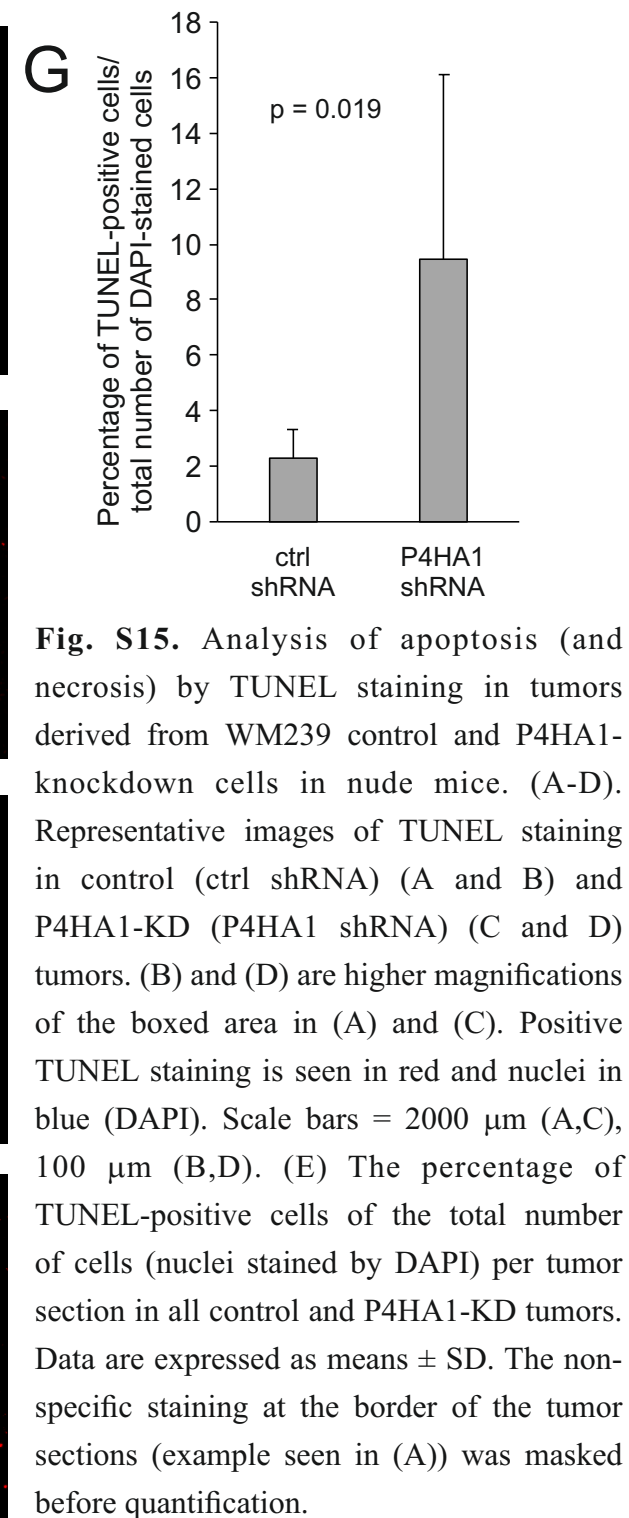

Supplement: Supplementary file 15 — Fig. S15. Analysis of apoptosis (and necrosis) by TUNEL staining in xenograft tumors derived from WM239 control and P4HA1‐knockdown cells. [file MOL2-14-742-s015.pdf]
