# Supplementary material for: Prolyl 4‐hydroxylase subunit alpha 1 (P4HA1) is a biomarker of poor prognosis in primary melanomas, and its depletion inhibits melanoma cell invasion and disrupts tumor blood vessel walls
Source: Mol Oncol. 2020 Feb 28;14(4):742–62. doi: 10.1002/1878-0261.12649 (PMC7138405; doi:10.1002/1878-0261.12649)
Supplement: Supplementary file 2 — Fig. S2. Prognostic value of selected potential marker genes of short survival in primary melanomas. [file MOL2-14-742-s002.pdf]

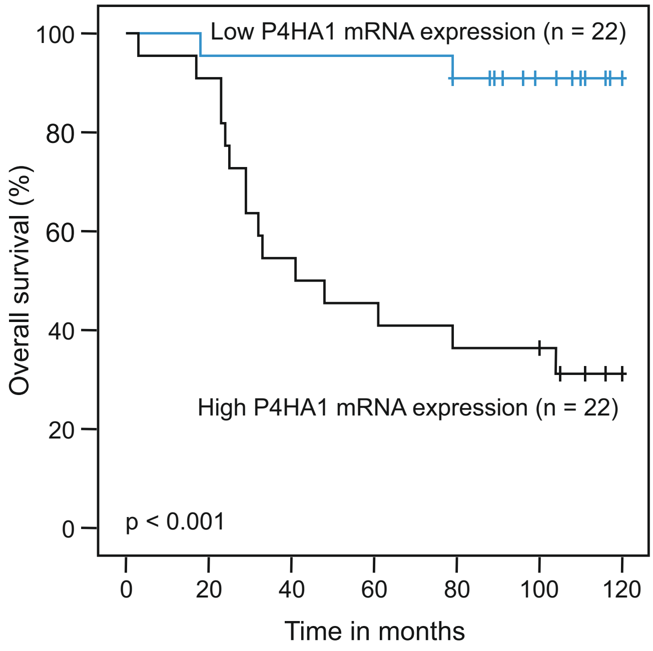

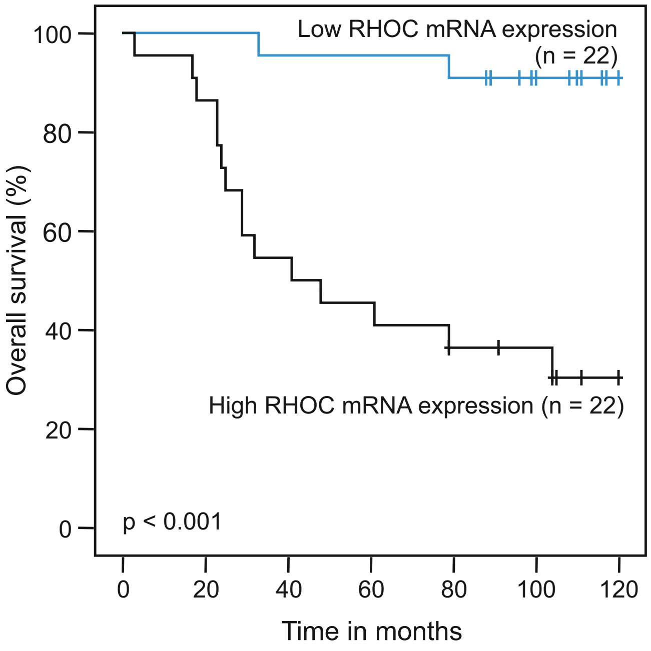

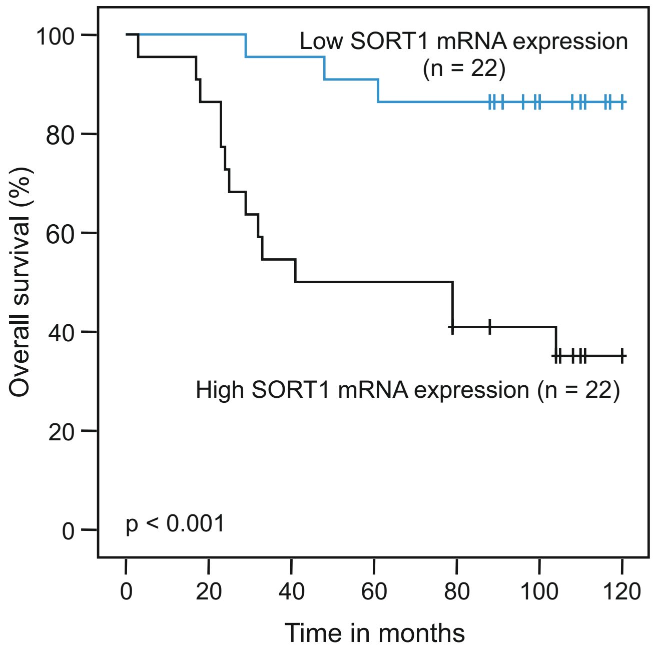

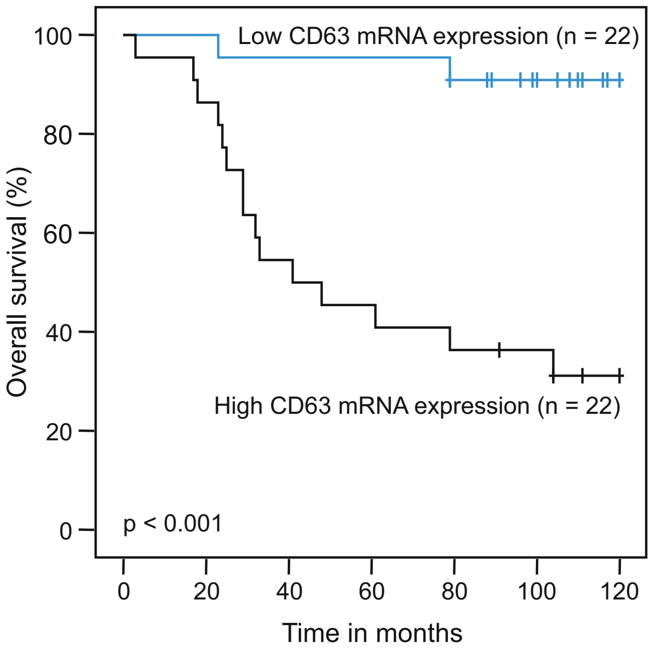

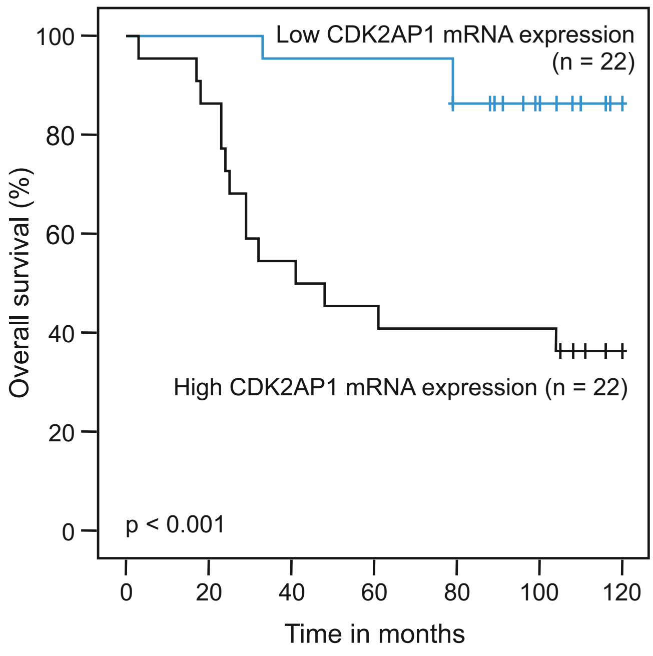

Fig. S2. Prognostic value of selected potential marker genes of short survival in primary melanomas. Kaplan-Meier survival curves for patients with primary melanomas that show low and high mRNA expression of selected genes in an independent RNA sequencing data set (GSE98394).
