# Supplementary material for: Prolyl 4‐hydroxylase subunit alpha 1 (P4HA1) is a biomarker of poor prognosis in primary melanomas, and its depletion inhibits melanoma cell invasion and disrupts tumor blood vessel walls
Source: Mol Oncol. 2020 Feb 28;14(4):742–62. doi: 10.1002/1878-0261.12649 (PMC7138405; doi:10.1002/1878-0261.12649)
Supplement: Supplementary file 4 — Fig. S4. Knockdown of P4HA1 protein in WM239 cells. [file MOL2-14-742-s004.pdf]

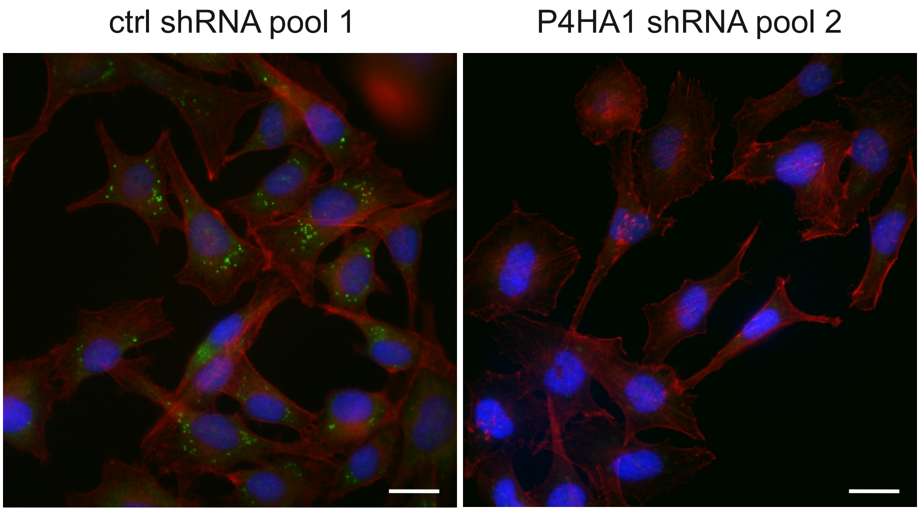

Fig. S4. Knockdown of P4HA1 protein in WM239 cells. Immunofluorescence image shows P4HA1 expression in WM239 control (ctrl shRNA) and P4HA1-KD (P4HA1 shRNA) cells. P4HA1 is seen in green, F-actin in red, and nuclei (DAPI) in blue. Scale bars = 25 μm.
