# Supplementary material for: Prolyl 4‐hydroxylase subunit alpha 1 (P4HA1) is a biomarker of poor prognosis in primary melanomas, and its depletion inhibits melanoma cell invasion and disrupts tumor blood vessel walls
Source: Mol Oncol. 2020 Feb 28;14(4):742–62. doi: 10.1002/1878-0261.12649 (PMC7138405; doi:10.1002/1878-0261.12649)
Supplement: Supplementary file 6 — Fig. S6. Effect of P4HA1 protein downregulation on CTHRC1 secretion in WM239 cells. [file MOL2-14-742-s006.pdf]

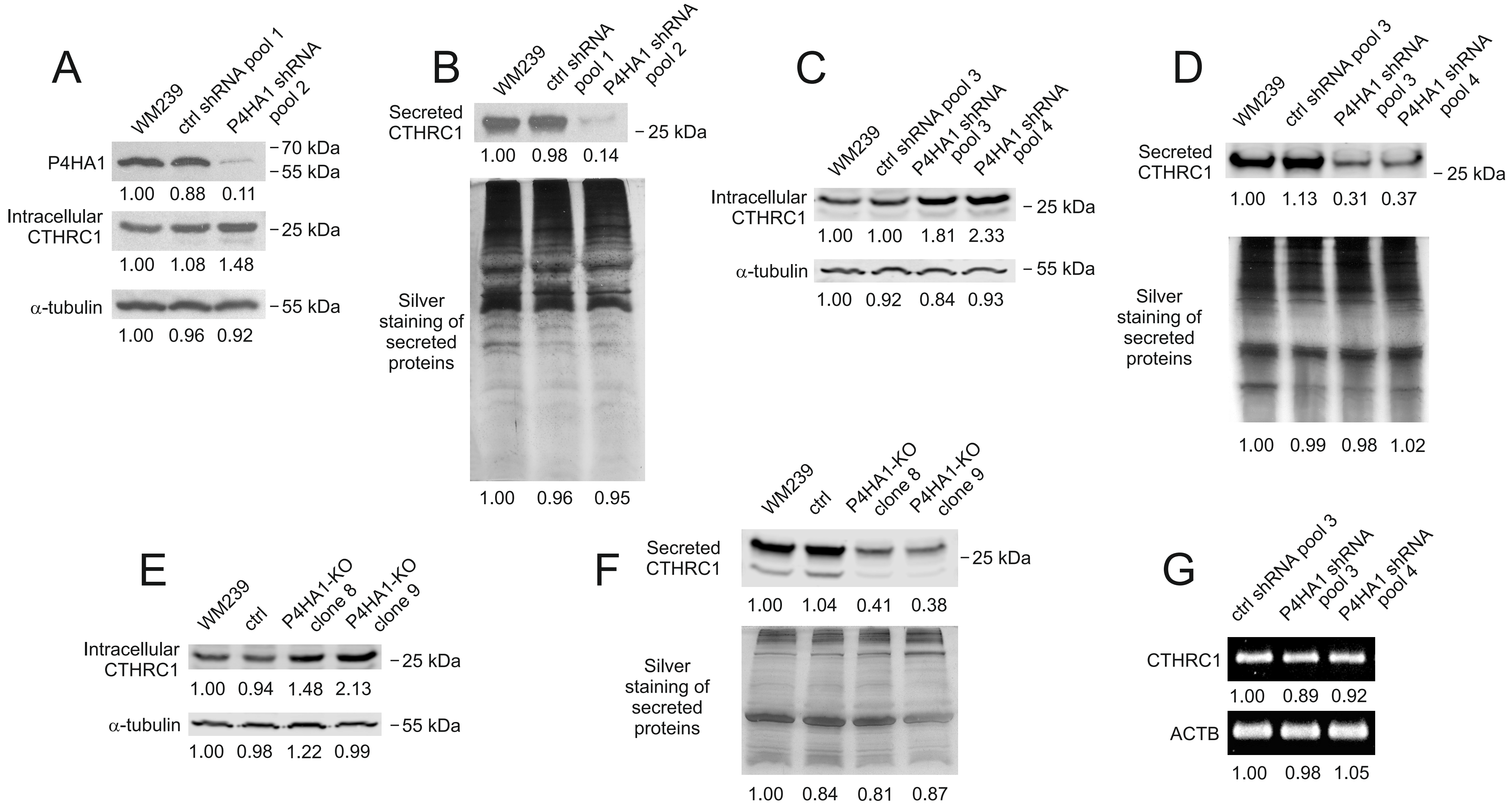

Fig. S6. Effect of P4HA1 protein downregulation on CTHRC1 secretion in WM239 cells. (A and B) Western blot analysis of P4HA1 and CTHRC1 protein expression in cell extracts (A) and CTHRC1 protein expression in the conditioned medium (B) of parental WM239, control (ctrl shRNA), and P4HA1-KD (P4HA1 shRNA) cells transduced with lentiviral shRNAs from Sigma-Aldrich/Merck. (C and D) Western blot analysis of CTHRC1 protein expression in cell extracts (C) and in the conditioned medium (D) of parental, control, and P4HA1-KD cells transduced with lentiviral shRNAs from Santa Cruz Biotechnology. (E and F) Western blot analysis of CTHRC1 protein expression in cell extracts (E) and in the conditioned medium (F) of parental, control, and P4HA1-knockout (P4HA1-KO) cells. Alpha-tubulin was used as a loading control for the cell extracts (A, C, and E) and silver staining to analyze the total secreted proteins in the conditioned medium (B, D, and F). (G) Semiquantitative RT-PCR analysis of CTHRC1 mRNA expression in control and P4HA1-KD cells. Beta-actin (ACTB) was used as a control.
