# Supplementary material for: Prolyl 4‐hydroxylase subunit alpha 1 (P4HA1) is a biomarker of poor prognosis in primary melanomas, and its depletion inhibits melanoma cell invasion and disrupts tumor blood vessel walls
Source: Mol Oncol. 2020 Feb 28;14(4):742–62. doi: 10.1002/1878-0261.12649 (PMC7138405; doi:10.1002/1878-0261.12649)
Supplement: Supplementary file 7 — Fig. S7. Effect of P4HA1 protein downregulation or prolyl 4‐hydroxylase inhibition on CTHRC1 secretion. [file MOL2-14-742-s007.pdf]

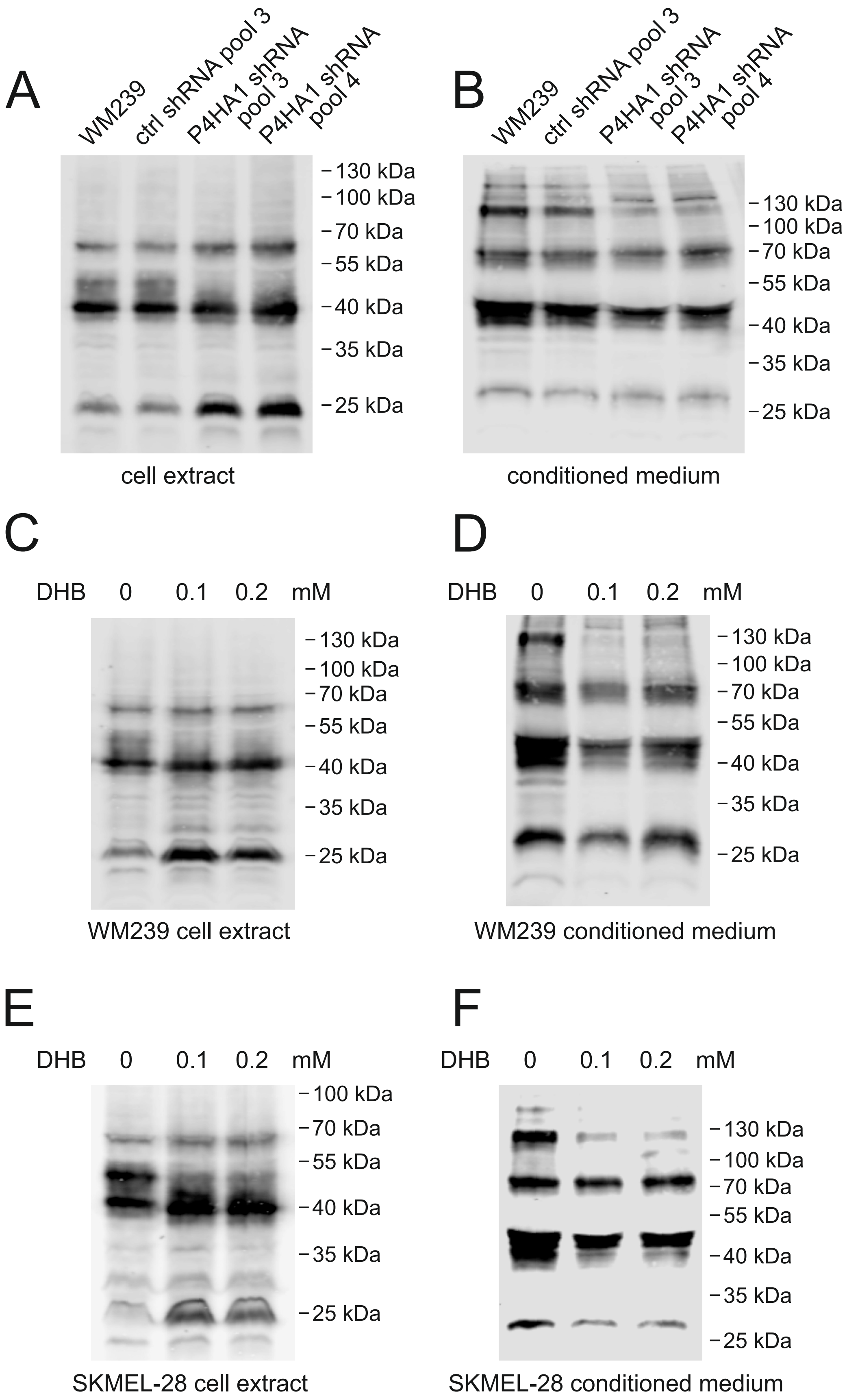

Fig. S7. Effect of P4HA1 protein downregulation or prolyl 4-hydroxylase inhibtion on CTHRC1 secretion. (A and B) Western blot analysis of CTHRC1 protein expression under non-reducing conditions in cell extracts (A) and in the conditioned medium (B) of parental WM239, control (ctrl shRNA), and P4HA1-KD (P4HA1 shRNA) cells. (C-F) Western blot analysis of CTHRC1 protein expression under non-reducing conditions in cell extracts (C and E) and in the conditioned medium (D and F) of WM239 (C and D) and SKMEL-28 (E and F) cells incubated without or with a prolyl 4-hydroxylase inhibitor 3,4-dihydroxybenzoic acid (DHB) for 18 hours.
