# Supplementary material for: Prolyl 4‐hydroxylase subunit alpha 1 (P4HA1) is a biomarker of poor prognosis in primary melanomas, and its depletion inhibits melanoma cell invasion and disrupts tumor blood vessel walls
Source: Mol Oncol. 2020 Feb 28;14(4):742–62. doi: 10.1002/1878-0261.12649 (PMC7138405; doi:10.1002/1878-0261.12649)
Supplement: Supplementary file 8 — Fig. S8. Effect of prolyl 4‐hydroxylase inhibition on cell adhesion and apoptosis/viability of SKMEL‐28 cells plated on fibronectin‐coated surfaces. [file MOL2-14-742-s008.pdf]

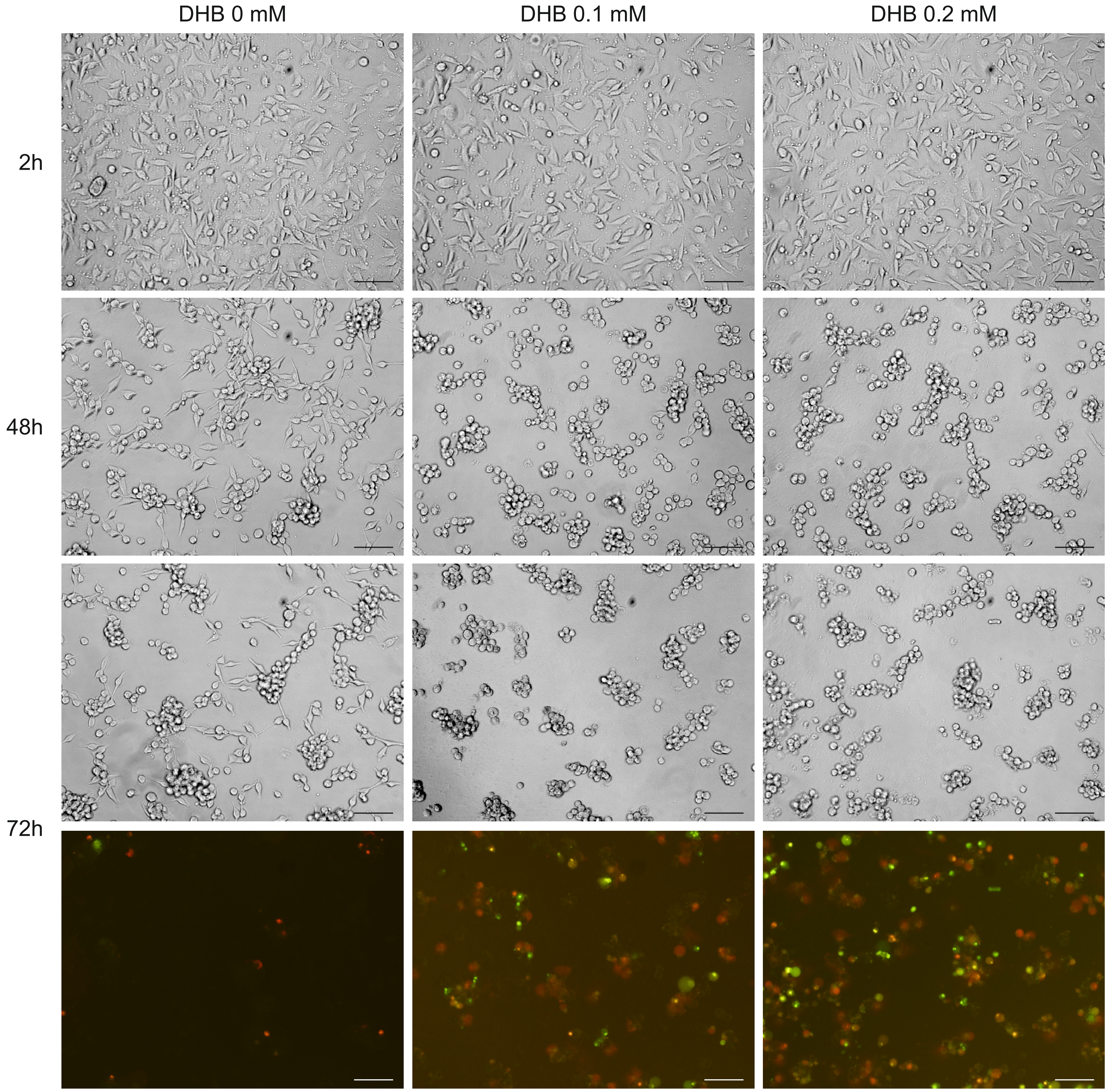

Fig. S8. Effect of prolyl 4-hydroxylase inhibition on cell adhesion and apoptosis/viability of SKMEL-28 cells plated on fibronectin-coated surfaces. SKMEL-28 cells were plated in serum-free media without or with a prolyl 4-hydroxylase inhibitor 3,4-dihydroxybenzoic acid (DHB) and photographed after 2h, 48h, and 72h incubation. Both phase-contrast and fluorescence images are shown after 72h incubation. Apoptotic cells (with activated caspase-3/7) are seen in green and dead cells (stained with propidium iodide) in red. Apoptotic, dead cells are seen in yellow. Scale bars = 100 μm.
