# Supplementary material for: Prolyl 4‐hydroxylase subunit alpha 1 (P4HA1) is a biomarker of poor prognosis in primary melanomas, and its depletion inhibits melanoma cell invasion and disrupts tumor blood vessel walls
Source: Mol Oncol. 2020 Feb 28;14(4):742–62. doi: 10.1002/1878-0261.12649 (PMC7138405; doi:10.1002/1878-0261.12649)
Supplement: Supplementary file 9 — Fig. S9. Effect of P4HA1 protein downregulation on CTHRC1 secretion in SKMEL‐28 cells. [file MOL2-14-742-s009.pdf]

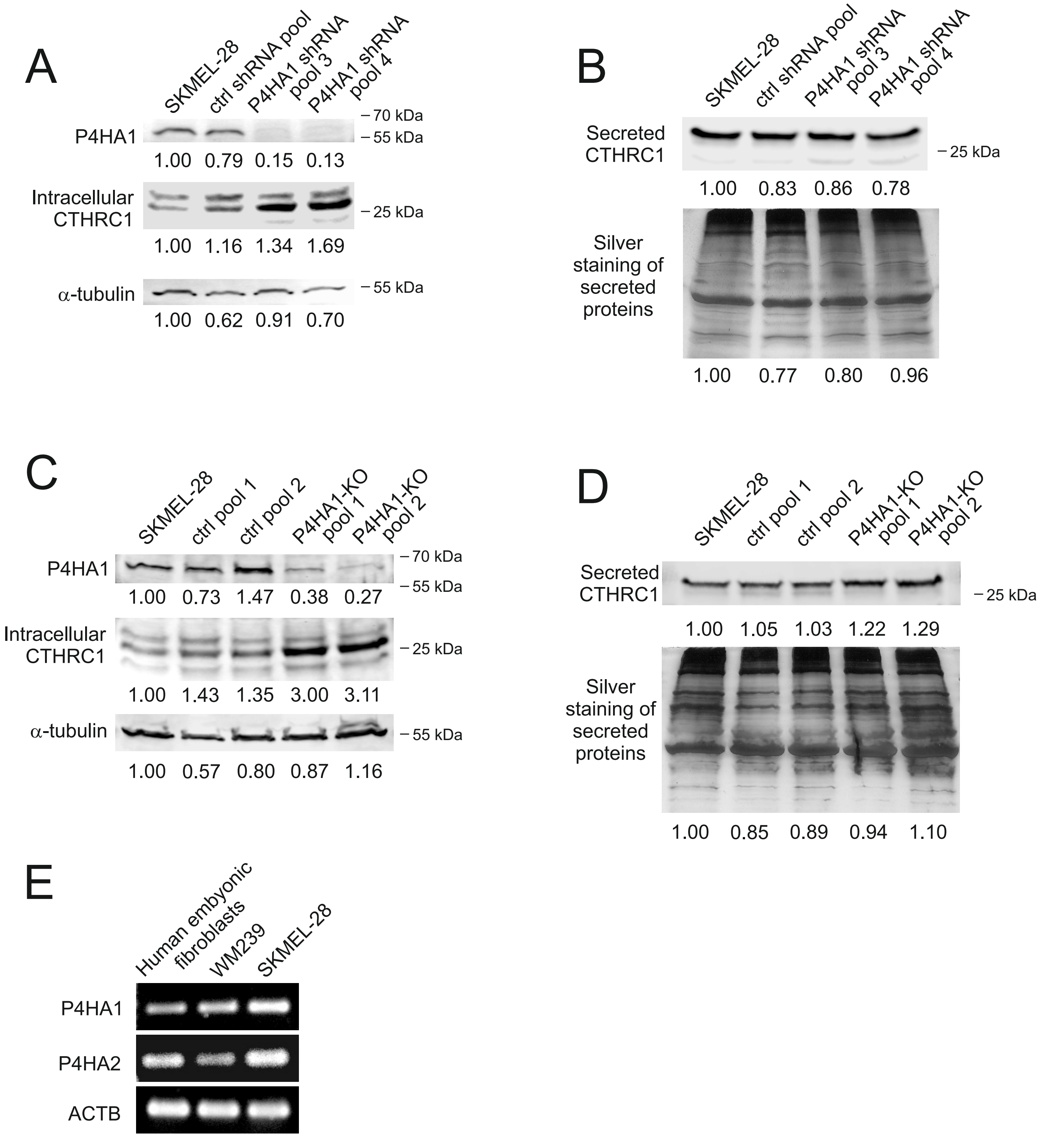

Fig. S9. Effect of P4HA1 protein downregulation on CTHRC1 secretion in SKMEL-28 cells. (A and B) Western blot analysis of P4HA1 and CTHRC1 protein expression in cell extracts (A) and CTHRC1 protein expression in the conditioned medium (B) of parental SKMEL-28, control (ctrl shRNA), and P4HA1-KD (P4HA1 shRNA) cells transduced with lentiviral shRNAs from Santa Cruz Biotechnology. (C and D) Western blot analysis of P4HA1 and CTHRC1 protein expression in cell extracts (C) and CTHRC1 protein expression in the conditioned medium (D) of parental, control, and P4HA1-knockout (P4HA1-KO) cells. Alpha-tubulin was used as a loading control for cell extracts (A and C) and silver staining to analyze the total secreted proteins in the conditioned medium (B and D). (E) Expression of P4HA1 and P4HA2 mRNAs in human embryonic fibroblasts and in WM239 and SKMEL-28 melanoma cell lines analyzed by semiquantitative PCR. Beta-actin (ACTB) was used as a control.
