# Supplementary material for: Prolyl 4‐hydroxylase subunit alpha 1 (P4HA1) is a biomarker of poor prognosis in primary melanomas, and its depletion inhibits melanoma cell invasion and disrupts tumor blood vessel walls
Source: Mol Oncol. 2020 Feb 28;14(4):742–62. doi: 10.1002/1878-0261.12649 (PMC7138405; doi:10.1002/1878-0261.12649)
Supplement: Supplementary file 10 — Fig. S10. Histochemical analysis of xenograft tumors derived from WM239 control and P4HA1‐knockdown cells. [file MOL2-14-742-s010.pdf]

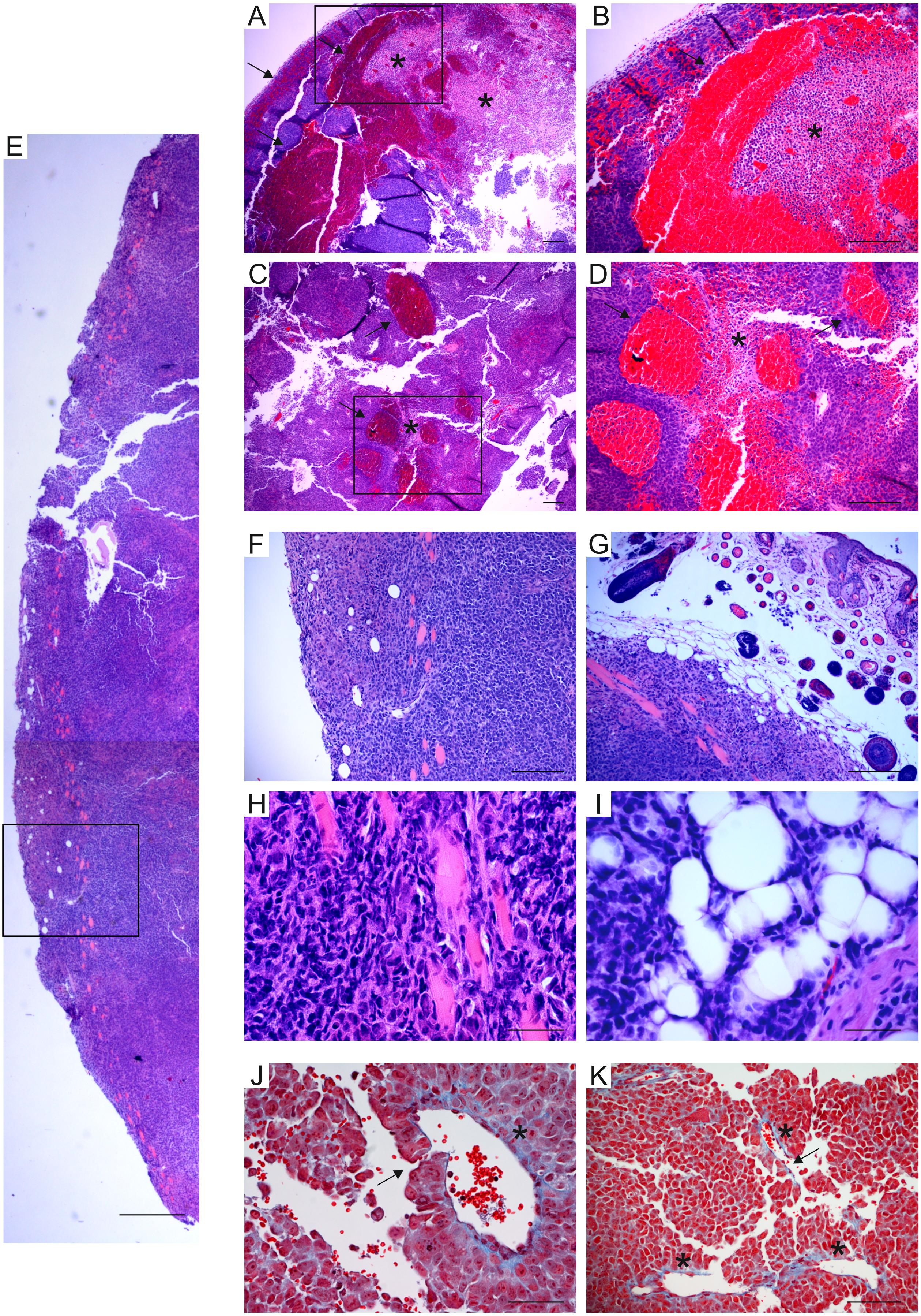

Fig. S10. Histochemical analysis of xenograft tumors derived from WM239 control and P4HA1-knockdown cells. (A-I) H&E staining in control (ctrl shRNA) and P4HA1-KD (P4HA1 shRNA) tumors. (A-D) Examples of hemorrhage (marked with asteriks) and necrosis (arrows) in P4HA1-KD tumors. (B) and (D) are higher manifications of the boxed areas in (A) and (C), respectively. (E-H) Examples of muscle tissue invasion in control tumors. (F) is a higher magnification of the boxed area in (E). (H) Higher magnification of striated muscle invasion shown previously in the main Fig. 7C. (I) Fat tissue invasion in control tumors (a higher magnification of the main Fig. 7E.) (J-K) Masson's trichrome staining in P4HA1-KD tumors. (J) is a higher magnification of the main Fig. 7J. Blood vessels are marked with asteriks. Note the red blood cells leaking from ruptured vessels (arrows). Scale bars = 200 µm (A-D), 500 µm (E), 200 µm (F,G), 50 µm (H-J), 100 µm (K).
