# Supplementary material for: Prolyl 4‐hydroxylase subunit alpha 1 (P4HA1) is a biomarker of poor prognosis in primary melanomas, and its depletion inhibits melanoma cell invasion and disrupts tumor blood vessel walls
Source: Mol Oncol. 2020 Feb 28;14(4):742–62. doi: 10.1002/1878-0261.12649 (PMC7138405; doi:10.1002/1878-0261.12649)
Supplement: Supplementary file 11 — Fig. S11. Immunohistochemical staining of COL‐I and CTHRC1 in frozen sections of xenograft tumors derived from WM239 control and P4HA1‐knockdown cells. [file MOL2-14-742-s011.pdf]

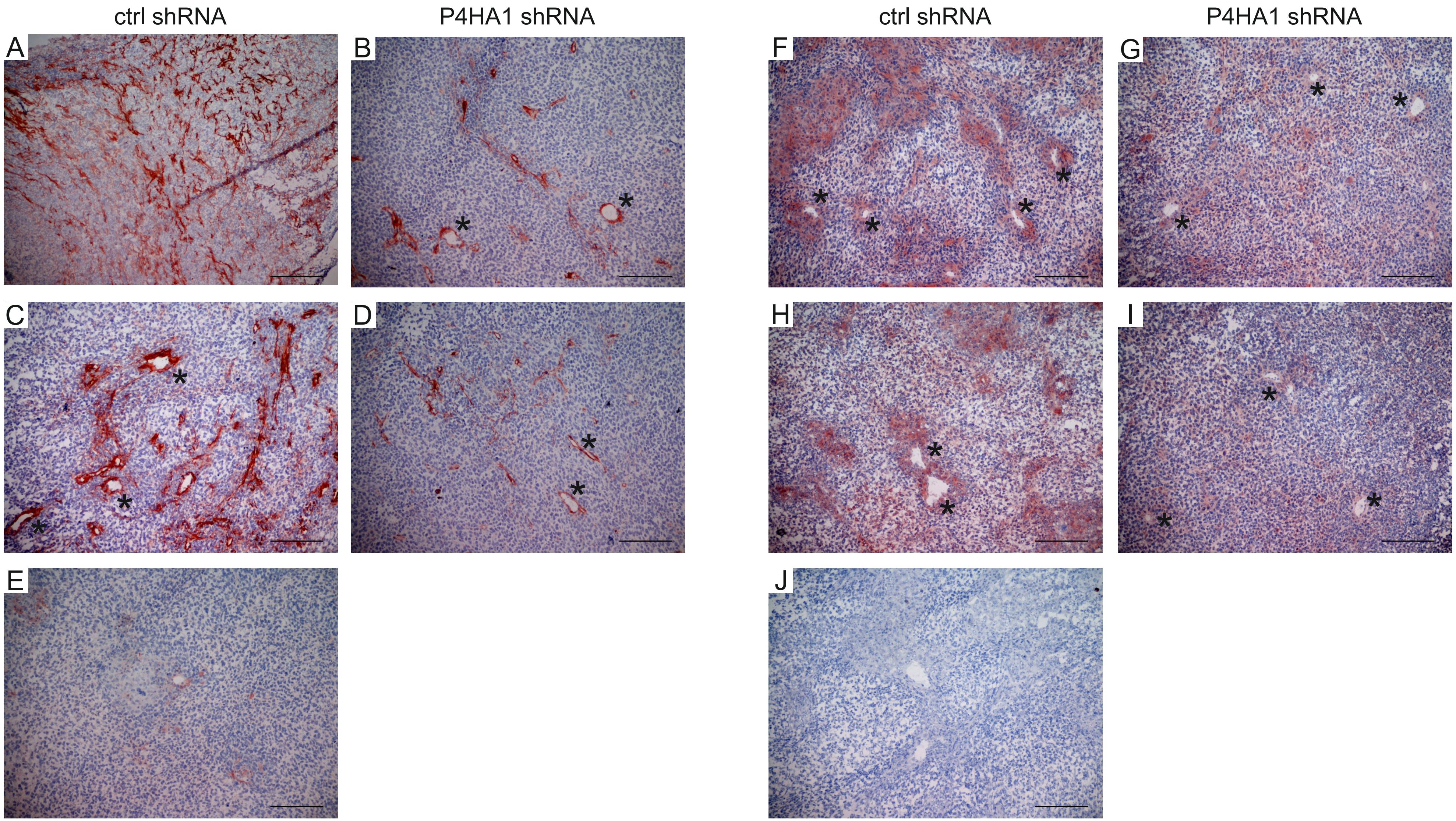

Fig. S11. Immunohistochemical staining of COL-I and CTHRC1 in frozen sections of xenograft tumors derived from WM239 control and P4HA1-knockdown cells. (A-D) Representative images of the COL-I immunostaining in WM239 control (ctrl shRNA) (A and C) and P4HA1-KD (P4HA1 shRNA) (B and D) tumors. (E) Control tumor stained with the normal mouse IgG1. (F-I) Representative images of the CTHRC1 immunostaining in control (F and H) and P4HA1-KD (G and I) tumors. (J) Control tumor stained with the normal rabbit IgG. Positive immunostaining is shown in red. Examples of blood vessels are marked with asterisks. Scale bars = 200 µm.
