# Supplementary material for: Prolyl 4‐hydroxylase subunit alpha 1 (P4HA1) is a biomarker of poor prognosis in primary melanomas, and its depletion inhibits melanoma cell invasion and disrupts tumor blood vessel walls
Source: Mol Oncol. 2020 Feb 28;14(4):742–62. doi: 10.1002/1878-0261.12649 (PMC7138405; doi:10.1002/1878-0261.12649)
Supplement: Supplementary file 12 — Fig. S12. Immunohistochemical staining of the endothelial cell marker CD31 in xenograft tumors derived from WM239 control and P4HA1‐knockdown cells. [file MOL2-14-742-s012.pdf]

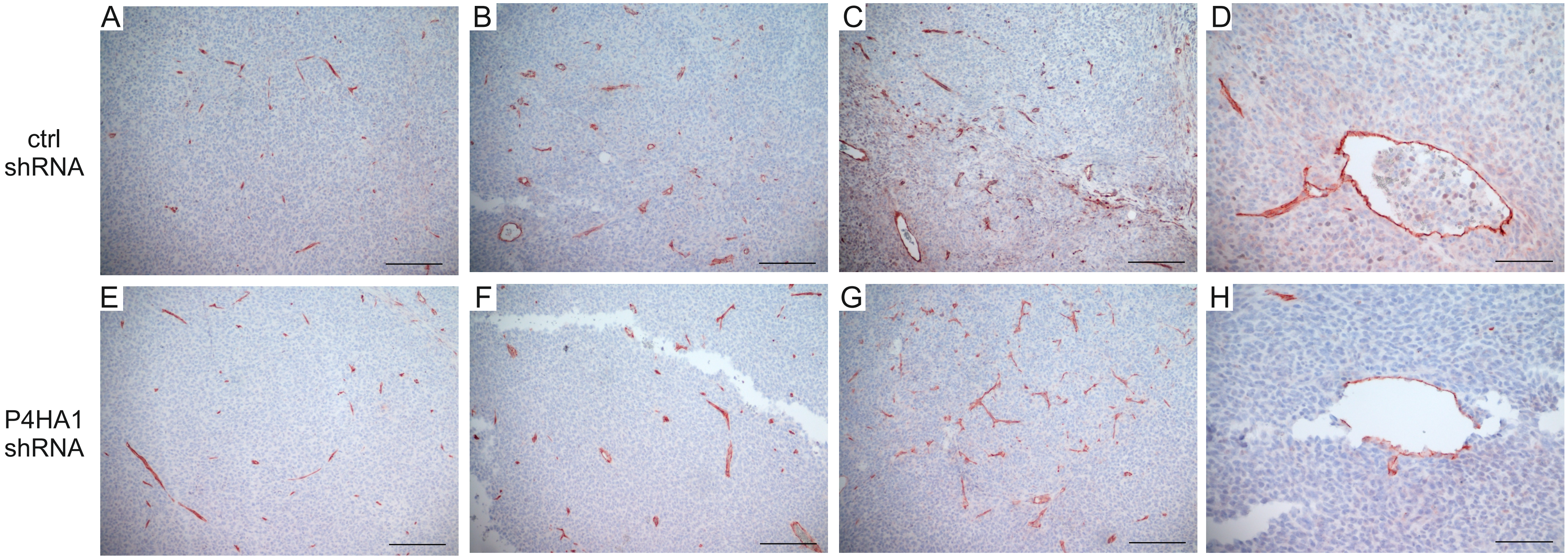

Fig. S12. Immunohistochemical staining of the endothelial cell marker CD31 in xenograft tumors derived from WM239 control and P4HA1-knockdown cells. Representative images of the CD31 staining in control (ctrl shRNA) (A-D) and P4HA1-KD (P4HA1 shRNA) (E-H) tumors. Positive immunostaining is seen in red. Note that the vessel in (D) contains tumor cells. Scale bars = 200 µm (A-C,E-G)), 100 µm (D,H).
