# Supplementary material for: Prolyl 4‐hydroxylase subunit alpha 1 (P4HA1) is a biomarker of poor prognosis in primary melanomas, and its depletion inhibits melanoma cell invasion and disrupts tumor blood vessel walls
Source: Mol Oncol. 2020 Feb 28;14(4):742–62. doi: 10.1002/1878-0261.12649 (PMC7138405; doi:10.1002/1878-0261.12649)
Supplement: Supplementary file 13 — Fig. S13. Immunohistochemical staining of the cell proliferation marker Ki‐67 in xenograft tumors derived from WM239 control and P4HA1‐knockdown cells. [file MOL2-14-742-s013.pdf]

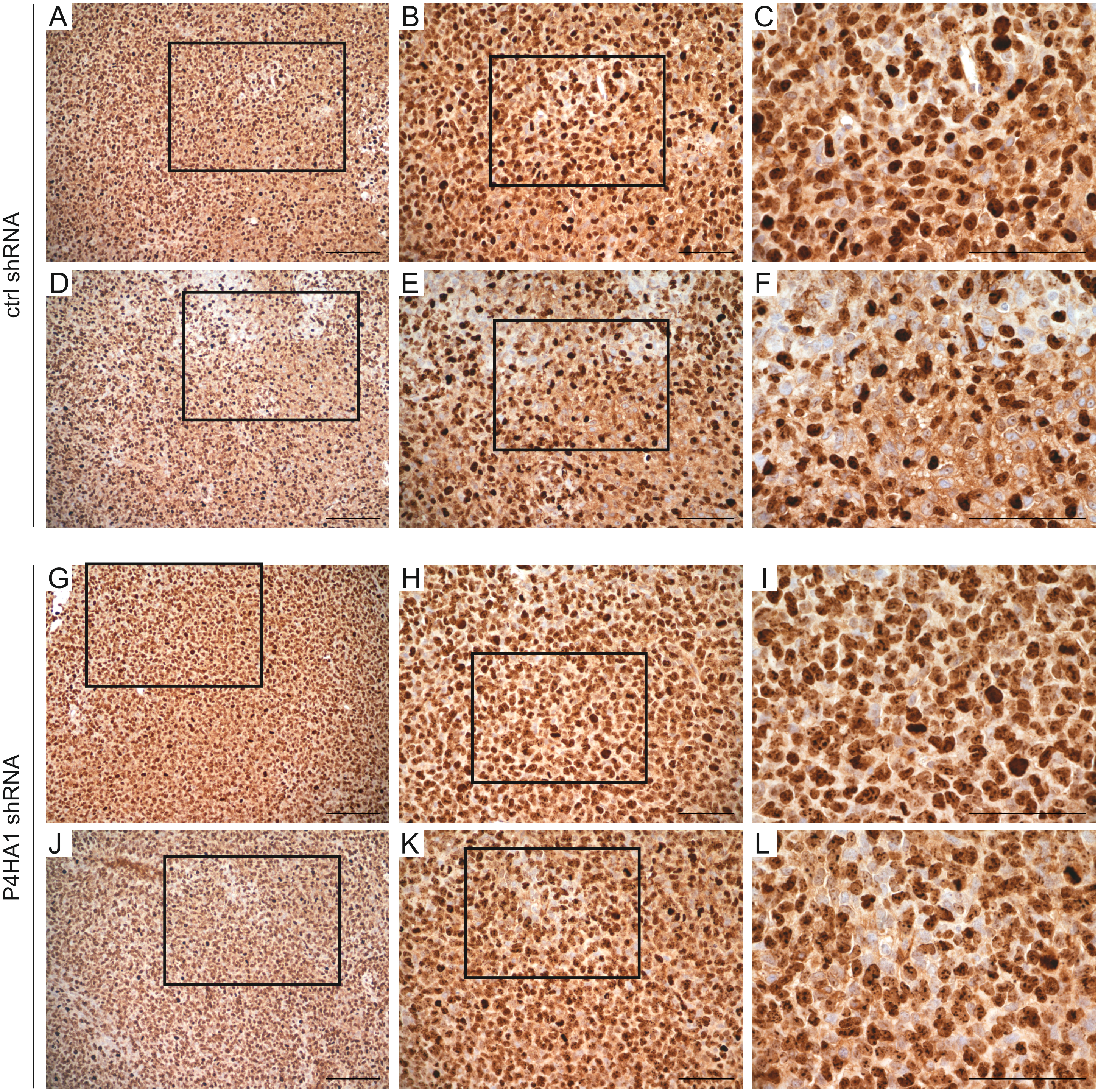

Fig. S13. Immunohistochemical staining of the cell proliferation marker Ki-67 in xenograft tumors derived from WM239 control and P4HA1-knockdown cells. Images of the Ki-67 staining in two control (ctrl shRNA) (A-F) and two P4HA1-KD (P4HA1 shRNA) (G-L) tumors (in non-necrotic areas). (B, E, H, and K) are higher magnifications of the boxed area in (A, D, G, and J), and (C, F, I, and L) are higher magnifications of the boxed area in (B, E, H, and K), respectively. Positive immunostaining is seen in brown. Scale bars = 200 μm (A, D, G, J), 100 μm (B-C, E-F, H-I, K-L).
