# Supplementary material for: Prolyl 4‐hydroxylase subunit alpha 1 (P4HA1) is a biomarker of poor prognosis in primary melanomas, and its depletion inhibits melanoma cell invasion and disrupts tumor blood vessel walls
Source: Mol Oncol. 2020 Feb 28;14(4):742–62. doi: 10.1002/1878-0261.12649 (PMC7138405; doi:10.1002/1878-0261.12649)
Supplement: Supplementary file 14 — Fig. S14. Immunohistochemical staining of the apoptosis marker cleaved caspase 3 in xenograft tumors derived from WM239 control and P4HA1‐knockdown cells. [file MOL2-14-742-s014.pdf]

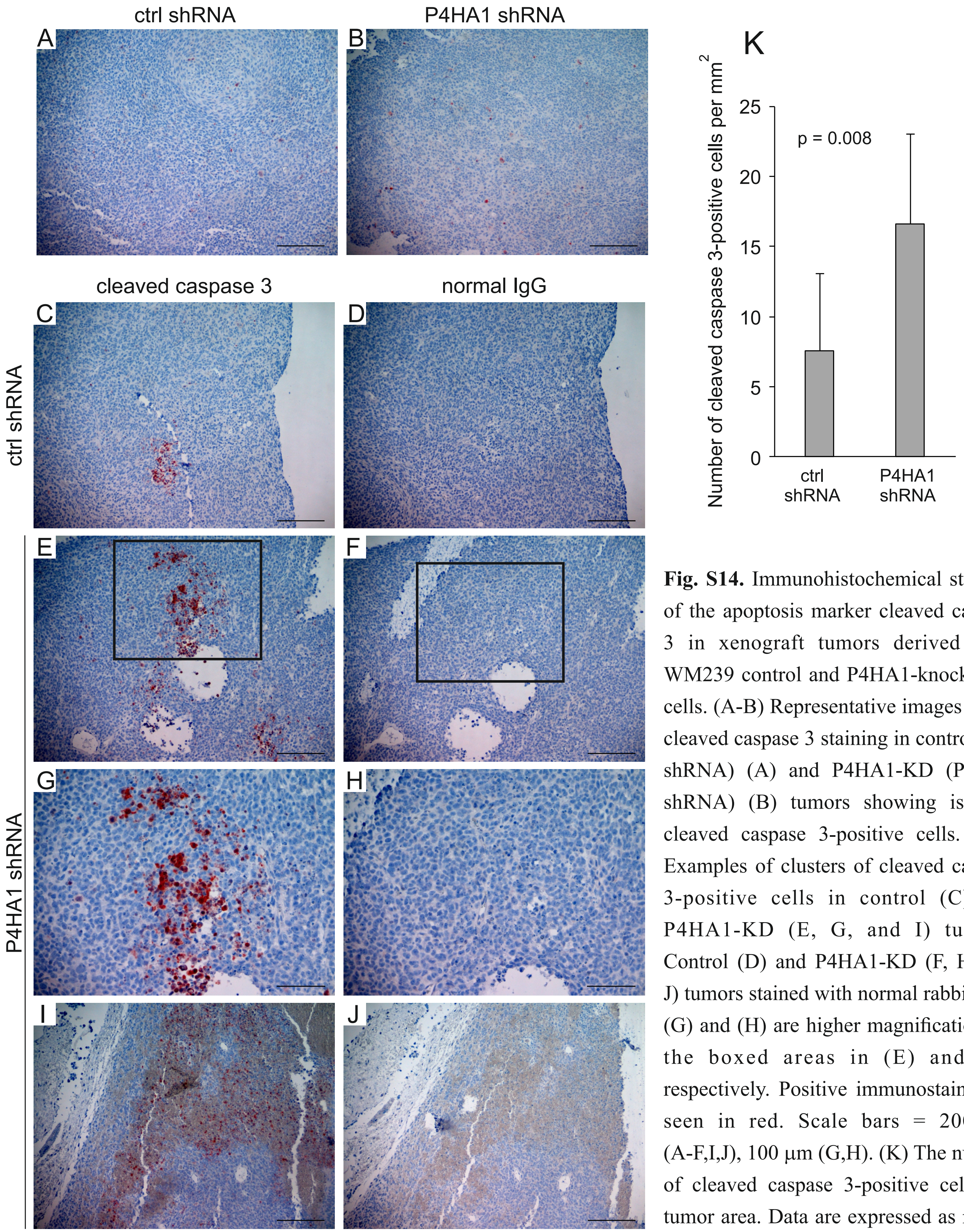

Fig. S14. Immunohistochemical staining of the apoptosis marker cleaved caspase 3 in xenograft tumors derived from WM239 control and P4HA1-knockdown cells. (A-B) Representative images of the cleaved caspase 3 staining in control (ctrl shRNA) (A) and P4HA1-KD (P4HA1 shRNA) (B) tumors showing isolated cleaved caspase 3-positive cells. (C-J) Examples of clusters of cleaved caspase 3-positive cells in control (C) and P4HA1-KD (E, G, and I) tumors. Control (D) and P4HA1-KD (F, H, and J) tumors stained with normal rabbit IgG. (G) and (H) are higher magnifications of the boxed areas in (E) and (F), respectively. Positive immunostaining is seen in red. Scale bars = 200 μm (A-F,I,J), 100 μm (G,H). (K) The number of cleaved caspase 3-positive cells per tumor area. Data are expressed as means ± SD.
