# Supplementary material for: Prolyl 4‐hydroxylase subunit alpha 1 (P4HA1) is a biomarker of poor prognosis in primary melanomas, and its depletion inhibits melanoma cell invasion and disrupts tumor blood vessel walls
Source: Mol Oncol. 2020 Feb 28;14(4):742–62. doi: 10.1002/1878-0261.12649 (PMC7138405; doi:10.1002/1878-0261.12649)
Supplement: Supplementary file 16 — Table S1. PCR variables. [file MOL2-14-742-s016.pdf]

Table S1. PCR variables.

Gene		1)	Sequence 5' to 3'	2)	3)	Source
ACTB		F	GCTCGTCGTCGACAACGGCTC	55	20	Invitrogen/Life Technologies
		R	CAAACATGATCTGGGTCATCTTCTC			
CTHRC1		F	AGCGCCTCTGAGATCCCCAA	59	22	Park *et al.*, 2013
		R	TGAACAAGTGCCAACCCAGA			
P4HA1	all variants	F	CCCCAGTCTTTGGCTCATCC	56	22-26	PrimerQuest
		R	CGTAACAGAGCTTTGGCTGC			
	variant 1	F	CCGAGCTACAGTACATGACCC	56	25	PrimerQuest
		R	TGGCTCATCTTTCCGTGCAA			
	variants 2 and 3	F	GGCTGAGGACTGCTTTGAGT	56	25	PrimerQuest
		R	ACCGTCTCCAAGTCTCCTGT			
	variant 4	F	GGCTGAGGACTGCTTTGAGT	56	34	PrimerQuest
		R	TGGCTCATCTTTCTGTAATTCCTCT			
P4HA2	variants 1 and 2	F	GCCTGCGCTGGAGGACCTTG	56	24	Gilkes *et al*., 2013
		R	TGTGCCTGGGTCCAGCCTGT			

1) Primer orientation, F=forward, R=reverse; 2) Annealing temperature (°C); 3) Number of PCR cycles

Gilkes, DM, Chaturvedi, P, Bajpai, S, Wong, CC, Wei, H, Pitcairn, S, Hubbi, ME, Wirtz, D and Semenza, GL (2013) Collagen prolyl hydroxylases are essential for breast cancer metastasis. *Cancer Res* **73,** 3285-3296.

Park, EH, Kim, S, Jo, JY, Kim, SJ, Hwang, Y, Kim, JM, Song, SY, Lee, DK and Koh, SS (2013) Collagen triple helix repeat containing-1 promotes pancreatic cancer progression by regulating migration and adhesion of tumor cells. *Carcinogenesis* **34**, 694-702.
