# Supplementary material for: Prolyl 4‐hydroxylase subunit alpha 1 (P4HA1) is a biomarker of poor prognosis in primary melanomas, and its depletion inhibits melanoma cell invasion and disrupts tumor blood vessel walls
Source: Mol Oncol. 2020 Feb 28;14(4):742–62. doi: 10.1002/1878-0261.12649 (PMC7138405; doi:10.1002/1878-0261.12649)
Supplement: Supplementary file 17 — Table S2. Significance Analysis of Microarrays (SAM) results of mRNA expression levels in primary melanomas associated most significantly with patient survival (higher expression in cases with short survival). [file MOL2-14-742-s017.pdf]

Table S2. Significance Analysis of Microarrays (SAM) results of mRNA expression levels in primary melanomas associated most significantly with patient survival (higher expression in cases with short survival).

Gene	Gene description	Gene ID	SAM score	False discovery rate q-value	Mean expression Alive	Mean expression Dead	Fold Dead vs alive	Fold Primary melanomas vs benign nevi	Fold Melanoma cells vs melanocytes
CNP	2',3'-cyclic nucleotide 3' phosphodiesterase	208912_s_at	4.205	0.001	832	1546	1.9	1.0	-1.3
P4HA1	prolyl 4-hydroxylase subunit alpha 1	207543_s_at	4.147	0.001	425	1021	2.4	1.4	4.3
RHOC	ras homolog family member C	200885_at	4.040	0.001	1115	2080	1.9	1.2	2.4
SERPINA3	serpin family A member 3	202376_at	3.946	0.001	396	1908	4.8	3.9	38.0
SLC27A3	solute carrier family 27 member 3	222217_s_at	3.888	0.001	333	1138	3.4	1.7	-6.1
SORT1	sortilin 1	212807_s_at	3.715	0.001	426	1064	2.5	1.8	-6.9
ATP1A1	ATPase Na+/K+ transporting subunit alpha 1	220948_s_at	3.675	0.001	2130	4235	2.0	1.1	-1.2
CD63	CD63 molecule	200663_at	3.665	0.001	6668	10900	1.6	1.1	-1.5
S100B	S100 calcium binding protein B	209686_at	3.635	0.001	940	3243	3.4	1.0	5.0
PLA1A	phospholipase A1 member A	219584_at	3.629	0.001	147	1276	8.7	1.5	-9.9
USP54	ubiquitin specific peptidase 54	227334_at	3.543	0.001	358	736	2.1	1.1	-3.0
ELOVL2	ELOVL fatty acid elongase 2	213712_at	3.539	0.001	132	609	4.6	1.7	1.5
CDK2AP1	cyclin dependent kinase 2 associated protein 1	201938_at	3.502	0.001	2961	4978	1.7	1.4	1.3
BIRC7	baculoviral IAP repeat containing 7	220451_s_at	3.471	0.001	196	680	3.5	1.2	-1.5
PACSIN2	protein kinase C and casein kinase substrate in neurons 2	201651_s_at	3.468	0.001	1564	2386	1.5	-1.1	1.2
MELTF	melanotransferrin	235911_at	3.417	0.001	402	1127	2.8	1.1	-1.6
BIN3	bridging integrator 3	222199_s_at	3.384	0.001	295	661	2.2	1.7	2.2
ARHGEF40	Rho guanine nucleotide exchange factor 40	220326_s_at	3.358	0.001	314	740	2.4	-1.1	-1.1
SHC1	SHC adaptor protein 1	214853_s_at	3.343	0.001	1906	3723	2.0	1.7	2.2
FN1	fibronectin 1	216442_x_at	3.278	0.001	3351	9698	2.9	2.5	3.5
HHATL	hedgehog acyltransferase like	223572_at	3.261	0.001	55	359	6.6	-12.7	1.1

TLNRD1	talin rod domain containing 1	223264_at	3.250	0.001	623	1626	2.6	1.2	-1.5
ARPC1B	actin related protein 2/3 complex subunit 1B	201954_at	3.229	0.001	1886	4217	2.2	2.3	1.6
PSMB4	proteasome subunit beta 4	202243_s_at	3.197	0.001	1759	2830	1.6	1.0	1.0
FKBP11	FKBP prolyl isomerase 11	219117_s_at	3.181	0.001	638	1557	2.4	3.4	-2.0
COL9A3	collagen type IX alpha 3 chain	204724_s_at	3.181	0.001	177	814	4.6	2.4	9.8
GNG5	G protein subunit gamma 5	207157_s_at	3.132	0.001	2374	3804	1.6	1.3	-1.2
SDC3	syndecan 3	202898_at	3.108	0.001	405	1175	2.9	-1.4	4.5
ETV5	ETS variant 5	230102_at	3.094	0.001	256	622	2.4	-2.0	1.4
TYR	tyrosinase	206630_at	3.084	0.001	3303	6694	2.0	-1.3	-62.1
HYOU1	hypoxia up-regulated 1	200825_s_at	3.079	0.001	920	1516	1.6	1.6	3.4
SOX10	SRY-box 10	209842_at	3.047	0.001	798	1717	2.2	-1.7	-1.4
TAF6	TATA-box binding protein associated factor 6	203572_s_at	3.038	0.001	377	668	1.8	1.1	1.3
PSRC1	proline and serine rich coiled-coil 1	201896_s_at	3.035	0.001	123	486	3.9	1.4	2.2
DUSP4	dual specificity phosphatase 4	204015_s_at	3.027	0.001	301	695	2.3	1.3	1.3
BACE2	beta-secretase 2	217867_x_at	3.013	0.001	714	1538	2.2	-1.1	-1.7
TBX2	T-box 2	40560_at	3.005	0.001	244	599	2.5	1.0	1.2
POPDC3	popeye domain containing 3	219926_at	2.980	0.001	98	339	3.5	2.2	-2.5
PLOD3	procollagen-lysine,2-oxoglutarate 5-dioxygenase 3	202185_at	2.979	0.001	1450	2767	1.9	1.8	1.8
MCAM	melanoma cell adhesion molecule	210869_s_at	2.971	0.001	426	1024	2.4	2.4	13.2
SRGAP1	SLIT-ROBO Rho GTPase activating protein 1	227484_at	2.954	0.001	268	452	1.7	-1.1	1.0
CALR	calreticulin	212953_x_at	2.942	0.001	1048	1574	1.5	-1.1	1.8
APOD	apolipoprotein D	201525_at	2.924	0.001	4028	8354	2.1	1.0	-14.9
ACSL3	acyl-CoA synthetase long chain family member 3	201662_s_at	2.922	0.001	659	1149	1.7	2.4	4.0
GLMP	glycosylated lysosomal membrane protein	225401_at	2.916	0.001	847	1932	2.3	1.2	-4.3
PLAT	plasminogen activator, tissue type	201860_s_at	2.909	0.001	418	929	2.2	2.1	11.8
CNDP1	carnosine dipeptidase 1	223699_at	2.891	0.001	71	254	3.6	1.9	1.3
LGALS3BP	galectin 3 binding protein	200923_at	2.890	0.001	1356	2181	1.6	1.4	-2.1
MYH10	myosin heavy chain 10	212372_at	2.885	0.001	971	1836	1.9	1.2	1.9

GJB1	gap junction protein beta 1	204973_at	2.880	0.001	319	651	2.0	-1.1	-1.2
HSP90B1	heat shock protein 90 beta family member 1	200598_s_at	2.880	0.001	799	1523	1.9	1.7	2.9
NES	nestin	218678_at	2.863	0.001	688	1363	2.0	1.5	5.6
UPP1	uridine phosphorylase 1	203234_at	2.861	0.001	265	618	2.3	4.0	1.4
IGFBP2	insulin like growth factor binding protein 2	202718_at	2.856	0.001	354	857	2.4	3.1	17.0
ODC1	ornithine decarboxylase 1	200790_at	2.848	0.001	677	1167	1.7	1.8	6.1
CHST11	carbohydrate sulfotransferase 11	226372_at	2.842	0.001	341	820	2.4	1.3	2.6
PYGL	glycogen phosphorylase L	202990_at	2.831	0.001	397	678	1.7	-3.4	2.3
BUD23	BUD23 rRNA methyltransferase and ribosome maturation factor	207628_s_at	2.830	0.001	670	1085	1.6	1.2	2.3
SHISA2	shisa family member 2	230493_at	2.824	0.001	156	490	3.2	1.0	2.7
ECM1	extracellular matrix protein 1	209365_s_at	2.823	0.001	880	1762	2.0	1.7	-1.9
TMEM8A	transmembrane protein 8A	221882_s_at	2.818	0.001	325	706	2.2	2.0	-1.9
PKM	pyruvate kinase M1/2	201251_at	2.781	0.001	1983	3556	1.8	1.3	4.2
MIA	MIA SH3 domain containing	206560_s_at	2.758	0.001	706	1912	2.7	1.5	9.2
ITGA10	integrin subunit alpha 10	206766_at	2.752	0.001	176	663	3.8	2.3	1.0
MAP3K11	mitogen-activated protein kinase kinase kinase 11	203652_at	2.750	0.001	389	714	1.8	1.0	-2.6
CFL2	cofilin 2	224663_s_at	2.750	0.001	270	516	1.9	1.2	1.2
ENO2	enolase 2	201313_at	2.749	0.001	182	572	3.1	2.2	-1.6
NID1	nidogen 1	202007_at	2.746	0.001	474	897	1.9	1.8	3.1
PARVB	parvin beta	204629_at	2.744	0.001	242	513	2.1	1.3	1.3
FAM167B	family with sequence similarity 167 member B	223657_at	2.740	0.001	97	234	2.4	1.2	-1.8
ETV4	ETS variant 4	1554576_a_at	2.737	0.001	189	360	1.9	1.3	ND
SH2B3	SH2B adaptor protein 3	203320_at	2.735	0.001	361	819	2.3	2.6	8.5
TSPAN3	tetraspanin 3	200972_at	2.734	0.001	1454	2322	1.6	1.0	-1.8
SCCPDH	saccharopine dehydrogenase (putative)	201825_s_at	2.701	0.001	290	585	2.0	1.3	1.5
LEF1	lymphoid enhancer binding factor 1	221558_s_at	2.701	0.001	770	1597	2.1	1.5	2.0
CSPG4	chondroitin sulfate proteoglycan 4	214297_at	2.701	0.001	131	705	5.4	-1.2	5.8
LMF2	lipase maturation factor 2	31837_at	2.698	0.001	674	1091	1.6	1.2	-1.1

IGSF8	immunoglobulin superfamily member 8	225025_at	2.694	0.001	271	526	1.9	1.2	-2.8
PLEKHB1	pleckstrin homology domain containing B1	209504_s_at	2.693	0.001	111	377	3.4	1.8	-1.1
MMP16	matrix metallopeptidase 16	223614_at	2.687	0.001	155	480	3.1	1.3	13.3
ITGA7	integrin subunit alpha 7	216331_at	2.685	0.001	229	507	2.2	2.0	1.0
SPON2	spondin 2	218638_s_at	2.684	0.001	434	997	2.3	-1.1	-2.2
PDIA6	protein disulfide isomerase family A member 6	207668_x_at	2.675	0.001	1464	2254	1.5	1.3	4.0
TIMP3	TIMP metallopeptidase inhibitor 3	201147_s_at	2.672	0.001	468	972	2.1	-1.4	27.7
UBE2C	ubiquitin conjugating enzyme E2 C	202954_at	2.670	0.001	373	718	1.9	2.4	7.0
HOXB7	homeobox B7	204779_s_at	2.641	0.001	342	663	1.9	-1.2	-1.6
TFPI2	tissue factor pathway inhibitor 2	209278_s_at	2.640	0.001	62	1154	18.6	19.1	33.0
S100A1	S100 calcium binding protein A1	205334_at	2.638	0.001	507	1129	2.2	1.8	-1.4
CRTAP	cartilage associated protein	1555889_a_at	2.623	0.001	2217	3820	1.7	-1.5	ND
MOK	MOK protein kinase	205130_at	2.619	0.001	322	540	1.7	-2.2	6.3
ANXA5	annexin A5	200782_at	2.613	0.001	2908	4610	1.6	1.0	-2.5
TYMS	thymidylate synthetase	202589_at	2.597	0.001	780	1505	1.9	2.6	10.4
SPCS1	signal peptidase complex subunit 1	217927_at	2.593	0.001	2414	3701	1.5	1.2	1.5
INPP5F	inositol polyphosphate-5-phosphatase F	230363_s_at	2.589	0.001	66	200	3.0	1.4	2.0
CNN3	calponin 3	201445_at	2.587	0.001	745	1508	2.0	1.2	2.6
GPI	glucose-6-phosphate isomerase	208308_s_at	2.586	0.001	1439	2248	1.6	1.3	1.5
NME4	NME/NM23 nucleoside diphosphate kinase 4	212739_s_at	2.585	0.001	540	977	1.8	1.2	-1.2
LAMTOR5	late endosomal/lysosomal adaptor, MAPK and MTOR activator 5	202300_at	2.581	0.001	1077	1821	1.7	1.2	-1.3
NUP93	nucleoporin 93	202188_at	2.580	0.001	431	664	1.5	1.1	-2.1
TGM2	transglutaminase 2	201042_at	2.580	0.001	154	336	2.2	2.0	6.1
FCRLA	Fc receptor like A	235372_at	2.579	0.001	376	645	1.7	2.8	-2.5
CDK16	cyclin dependent kinase 16	208824_x_at	2.577	0.001	281	431	1.5	1.2	1.2
SCRG1	stimulator of chondrogenesis 1	205475_at	2.572	0.001	94	797	8.5	-1.2	13.0
RPL38	ribosomal protein L38	221943_x_at	2.559	0.001	1058	1591	1.5	-1.8	1.0
TMEM229B	transmembrane protein 229B	227544_at	2.554	0.001	253	414	1.6	1.1	1.0

DUSP6	dual specificity phosphatase 6	208892_s_at	2.551	0.001	810	1644	2.0	1.4	22.5
PLOD2	procollagen-lysine,2-oxoglutarate 5-dioxygenase 2	202619_s_at	2.548	0.001	180	393	2.2	1.9	3.8
WDR77	WD repeat domain 77	201421_s_at	2.545	0.001	331	510	1.5	1.3	1.6
FAM210B	family with sequence similarity 210 member B	224690_at	2.545	0.001	1404	2309	1.6	1.2	-1.5
S100A6	S100 calcium binding protein A6	217728_at	2.528	0.001	4254	6431	1.5	1.4	2.6
ST3GAL6	ST3 beta-galactoside alpha-2,3-sialyltransferase 6	210942_s_at	2.526	0.001	335	700	2.1	-1.7	-2.8
NT5DC3	5'-nucleotidase domain containing 3	218786_at	2.521	0.001	141	278	2.0	1.5	1.2
METRN	meteorin, glial cell differentiation regulator	219051_x_at	2.519	0.001	175	341	1.9	1.2	-1.1
ERBB3	erb-b2 receptor tyrosine kinase 3	226213_at	2.517	0.001	2011	3310	1.6	-1.5	-1.5
GAS7	growth arrest specific 7	207704_s_at	2.516	0.001	443	698	1.6	-1.2	3.7
ZNF697	zinc finger protein 697	227080_at	2.515	0.001	516	1132	2.2	1.8	2.9
GPRC5B	G protein-coupled receptor class C group 5 member B	203632_s_at	2.503	0.001	328	598	1.8	-1.9	-2.9
KIRREL1	kirre like nephrin family adhesion molecule 1	225303_at	2.499	0.001	293	593	2.0	1.6	3.5
PXN	paxillin	201087_at	2.488	0.001	316	491	1.6	1.2	2.0
CNPY2	canopy FGF signaling regulator 2	209796_s_at	2.468	0.001	583	907	1.6	1.4	1.4
NPAS2	neuronal PAS domain protein 2	213462_at	2.462	0.001	160	321	2.0	-1.1	1.8
VEGFA	vascular endothelial growth factor A	212171_x_at	2.461	0.001	338	537	1.6	1.5	2.8
BRI3	brain protein I3	223376_s_at	2.447	0.001	1188	2125	1.8	1.0	-1.6
OLIG1	oligodendrocyte transcription factor 1	228170_at	2.442	0.001	29	165	5.7	5.8	1.3
ARFGAP1	ADP ribosylation factor GTPase activating protein 1	234001_s_at	2.430	0.001	215	399	1.9	1.2	-1.5
PLOD1	procollagen-lysine,2-oxoglutarate 5-dioxygenase 1	200827_at	2.428	0.001	371	721	1.9	1.4	4.7
SELENON	selenoprotein N	224659_at	2.427	0.001	292	544	1.9	1.1	1.5
TMEM98	transmembrane protein 98	223170_at	2.410	0.001	684	1314	1.9	-1.3	-2.6
ABCD1	ATP binding cassette subfamily D member 1	205142_x_at	2.405	0.001	244	501	2.1	1.4	-1.9

GNG11	G protein subunit gamma 11	204115_at	2.403	0.001	455	890	2.0	1.3	1.7
TOMM34	translocase of outer mitochondrial membrane 34	201870_at	2.390	0.001	310	473	1.5	1.2	1.9
TIMM50	translocase of inner mitochondrial membrane 50	224913_s_at	2.387	0.001	486	772	1.6	1.8	1.2
WIPF1	WAS/WASL interacting protein family member 1	202665_s_at	2.387	0.001	212	406	1.9	1.7	2.2
ACTN4	actinin alpha 4	200601_at	2.386	0.001	937	1474	1.6	-1.2	2.9
CCDC50	coiled-coil domain containing 50	225331_at	2.383	0.001	620	1041	1.7	1.0	-1.2
ENTPD6	ectonucleoside triphosphate diphosphohydrolase 6	201704_at	2.381	0.001	331	529	1.6	1.3	2.4
ZNF827	zinc finger protein 827	243618_s_at	2.378	0.001	206	485	2.4	1.6	2.9
MRGBP	MRG domain binding protein	218586_at	2.376	0.001	116	318	2.7	1.7	1.3
CERS2	ceramide synthase 2	222212_s_at	2.370	0.001	919	1469	1.6	1.1	-1.2
GSS	glutathione synthetase	211630_s_at	2.367	0.001	423	652	1.5	1.2	1.9
ROPN1	rhophilin associated tail protein 1	231535_x_at	2.361	0.001	232	491	2.1	-1.1	1.3
PFKFB4	6-phosphofructo-2-kinase/fructose-2,6-biphosphatase 4	228499_at	2.349	0.001	239	416	1.7	1.8	3.5
ZMAT3	zinc finger matrin-type 3	219628_at	2.348	0.001	674	1023	1.5	1.0	-2.7
PCDH9	protocadherin 9	219737_s_at	2.348	0.001	92	213	2.3	1.7	2.2
CTHRC1	collagen triple helix repeat containing 1	225681_at	2.326	0.001	1929	3364	1.7	4.4	102.7
TIMP1	TIMP metallopeptidase inhibitor 1	201666_at	2.322	0.001	3222	6096	1.9	2.4	2.3
GDF15	growth differentiation factor 15	221577_x_at	2.321	0.001	274	881	3.2	4.1	4.9
ITGB3	integrin subunit beta 3	204627_s_at	2.318	0.001	71	236	3.3	3.4	16.5
TMC6	transmembrane channel like 6	204328_at	2.318	0.001	320	515	1.6	1.2	1.2
BEST1	bestrophin 1	1554442_at	2.313	0.001	360	576	1.6	1.2	ND
TMEM106C	transmembrane protein 106C	201764_at	2.301	0.001	591	903	1.5	1.4	1.9
SPRY4	sprouty RTK signaling antagonist 4	221489_s_at	2.291	0.001	472	1078	2.3	1.0	13.2
OSBPL10	oxysterol binding protein like 10	219073_s_at	2.278	0.001	254	544	2.1	1.4	3.1
SLC16A3	solute carrier family 16 member 3	202856_s_at	2.272	0.001	103	288	2.8	3.6	10.5
HEY1	hes related family bHLH transcription factor with YRPW motif 1	44783_s_at	2.269	0.001	997	1843	1.8	3.1	1.6

ZNF362	zinc finger protein 362	226820_at	2.267	0.001	674	1095	1.6	-1.2	-1.1
COL4A1	collagen type IV alpha 1 chain	211981_at	2.267	0.001	614	1088	1.8	3.0	2.7
FUCA2	alpha-L-fucosidase 2	223120_at	2.262	0.001	446	755	1.7	1.4	-1.1
PIK3R3	phosphoinositide-3-kinase regulatory subunit 3	202743_at	2.246	0.001	236	598	2.5	4.0	-2.5
ARNT2	aryl hydrocarbon receptor nuclear translocator 2	202986_at	2.244	0.001	179	380	2.1	-1.4	-1.3
SV2A	synaptic vesicle glycoprotein 2A	203069_at	2.241	0.001	145	303	2.1	1.3	1.3
COL4A2	collagen type IV alpha 2 chain	211964_at	2.238	0.001	1324	2396	1.8	2.4	2.0
LGALS1	galectin 1	201105_at	2.235	0.001	5608	9216	1.6	1.7	1.3
LY6E	lymphocyte antigen 6 family member E	202145_at	2.229	0.001	865	1365	1.6	1.5	3.1
LOXL3	lysyl oxidase like 3	228253_at	2.227	0.001	207	528	2.6	1.9	7.8
WDR91	WD repeat domain 91	218971_s_at	2.221	0.001	232	422	1.8	1.2	-1.9
COL11A2	collagen type XI alpha 2 chain	213870_at	2.219	0.001	123	244	2.0	-1.1	1.2
PLPP4	phospholipid phosphatase 4	236044_at	2.219	0.001	93	241	2.6	1.2	1.4
MAGED2	MAGE family member D2	208682_s_at	2.218	0.001	662	1246	1.9	1.0	-1.2
ATP6V0E2	ATPase H+ transporting V0 subunit e2	213587_s_at	2.211	0.001	443	899	2.0	2.1	4.3
WARS	tryptophanyl-tRNA synthetase	200629_at	2.205	0.001	1342	2308	1.7	4.4	1.3
MLPH	melanophilin	218211_s_at	2.204	0.001	1284	2403	1.9	-1.1	-7.2
FAM222B	family with sequence similarity 222 member B	218464_s_at	2.203	0.001	503	816	1.6	1.0	1.0
MMP1	matrix metallopeptidase 1	204475_at	2.195	0.001	452	2068	4.6	48.3	161.9
EIF5A	eukaryotic translation initiation factor 5A	213753_x_at	2.195	0.001	450	681	1.5	-1.5	1.3
CD276	CD276 molecule	224859_at	2.184	0.001	556	924	1.7	1.1	1.2
MCM7	minichromosome maintenance complex component 7	208795_s_at	2.182	0.001	434	710	1.6	1.3	3.9
OAF	out at first homolog	225510_at	2.177	0.001	362	556	1.5	1.3	1.8
GBAP1	glucosylceramidase beta pseudogene 1	210589_s_at	2.175	0.001	498	837	1.7	1.5	-1.1
SOX13	SRY-box 13	209736_at	2.174	0.002	294	450	1.5	1.1	-2.1
TMEM163	transmembrane protein 163	1552626_a_at	2.174	0.002	237	955	4.0	5.3	ND

SPP1	secreted phosphoprotein 1	209875_s_at	2.167	0.002	1695	4095	2.4	52.5	7.1
COL5A1	collagen type V alpha 1 chain	212489_at	2.167	0.002	351	687	2.0	1.8	1.2
TUSC3	tumor suppressor candidate 3	213423_x_at	2.163	0.002	371	637	1.7	-1.2	4.6
YTHDF1	YTH N6-methyladenosine RNA binding protein 1	221741_s_at	2.162	0.002	814	1269	1.6	1.2	-1.1
SEMA4C	semaphorin 4C	46665_at	2.157	0.002	833	1386	1.7	-1.1	-1.6
AEBP1	AE binding protein 1	201792_at	2.150	0.002	1590	3346	2.1	2.0	-3.6
USP13	ubiquitin specific peptidase 13	205356_at	2.138	0.002	427	791	1.9	1.1	2.3
WFDC1	WAP four-disulfide core domain 1	219478_at	2.131	0.002	146	306	2.1	1.7	1.0
DAAM2	dishevelled associated activator of morphogenesis 2	212793_at	2.131	0.002	301	512	1.7	1.1	1.2
IGFBP5	insulin like growth factor binding protein 5	211959_at	2.129	0.002	1225	2252	1.8	1.0	1.6
IGSF3	immunoglobulin superfamily member 3	202421_at	2.124	0.002	1088	1830	1.7	1.0	-1.4
FKBP9	FKBP prolyl isomerase 9	212169_at	2.120	0.002	781	1260	1.6	-1.4	1.2
IQGAP3	IQ motif containing GTPase activating protein 3	229538_s_at	2.119	0.002	113	214	1.9	2.0	3.5
GNAI2	G protein subunit alpha i2	201040_at	2.117	0.002	639	1156	1.8	1.2	1.2
SHROOM2	shroom family member 2	204967_at	2.113	0.002	229	426	1.9	1.4	3.1
PHF19	PHD finger protein 19	227212_s_at	2.112	0.002	162	306	1.9	1.7	5.1
GSTO1	glutathione S-transferase omega 1	201470_at	2.110	0.002	2029	3264	1.6	1.6	-3.0
RHBDF1	rhomboid 5 homolog 1	218686_s_at	2.109	0.002	391	614	1.6	1.0	1.3
PARP1	poly(ADP-ribose) polymerase 1	208644_at	2.091	0.002	601	910	1.5	1.4	1.6
GYPC	glycophorin C (Gerbich blood group)	202947_s_at	2.087	0.002	904	1624	1.8	1.0	-1.5
VGF	VGF nerve growth factor inducible	205586_x_at	2.084	0.002	112	348	3.1	1.9	1.8
RAB17	RAB17, member RAS oncogene family	218931_at	2.082	0.002	231	479	2.1	1.6	1.4
B4GAT1	beta-1,4-glucuronyltransferase 1	203188_at	2.080	0.002	376	586	1.6	-1.3	-1.2
TAFA5	TAFA chemokine like family member 5	229655_at	2.073	0.002	235	497	2.1	-1.1	-1.7
PNKD	PNKD metallo-beta-lactamase domain containing	233177_s_at	2.071	0.002	299	512	1.7	1.8	2.5
OLFML3	olfactomedin like 3	218162_at	2.062	0.002	534	865	1.6	1.2	4.3

IL13RA2	interleukin 13 receptor subunit alpha 2	206172_at	2.062	0.002	88	308	3.5	7.2	1.2
CABLES2	Cdk5 and Abl enzyme substrate 2	226004_at	2.059	0.002	159	305	1.9	1.1	-1.1
SLC7A5	solute carrier family 7 member 5	201195_s_at	2.058	0.002	668	1050	1.6	1.7	-1.7
CDC42SE1	CDC42 small effector 1	229120_s_at	2.053	0.002	642	1074	1.7	-1.5	1.2
SEPTIN9	septin 9	208657_s_at	2.051	0.002	286	444	1.5	1.0	-1.4
SERPINE2	serpin family E member 2	212190_at	2.048	0.002	4795	9279	1.9	1.0	3.1
SIRPA	signal regulatory protein alpha	202896_s_at	2.048	0.002	287	451	1.6	1.2	-5.9
TYRO3	TYRO3 protein tyrosine kinase	211432_s_at	2.041	0.002	347	536	1.5	-1.2	1.3
PDCD6	programmed cell death 6	203415_at	2.038	0.002	721	1116	1.5	1.4	1.1
USP11	ubiquitin specific peptidase 11	208723_at	2.029	0.002	513	785	1.5	-1.1	-1.2
IGLL3P	immunoglobulin lambda like polypeptide 3, pseudogene	215946_x_at	2.020	0.002	224	571	2.5	2.7	-1.1
ADRM1	adhesion regulating molecule 1	201281_at	2.015	0.002	635	983	1.5	1.5	1.6
TPP1	tripeptidyl peptidase 1	200742_s_at	2.012	0.002	983	1496	1.5	-1.4	-5.2
PAQR8	progestin and adipoQ receptor family member 8	227626_at	2.008	0.002	210	349	1.7	-1.1	-4.2
APOE	apolipoprotein E	203382_s_at	2.006	0.002	1655	2884	1.7	1.1	-22.1
TMEM158	transmembrane protein 158 (gene/pseudogene)	213338_at	2.004	0.002	312	791	2.5	3.4	7.0
SLC26A2	solute carrier family 26 member 2	205097_at	2.004	0.002	639	959	1.5	2.2	2.6
MMP14	matrix metallopeptidase 14	160020_at	1.998	0.002	501	882	1.8	1.0	1.0
ADCY1	adenylate cyclase 1	213245_at	1.998	0.002	83	277	3.3	2.7	-1.4
QPRT	quinolinate phosphoribosyltransferase	242414_at	1.994	0.002	470	728	1.5	-1.2	-8.2
LDLRAP1	low density lipoprotein receptor adaptor protein 1	221790_s_at	1.993	0.002	193	328	1.7	1.1	-1.1
AURKA	aurora kinase A	204092_s_at	1.991	0.002	173	332	1.9	2.6	6.5
STC1	stanniocalcin 1	204597_x_at	1.987	0.002	81	244	3.0	2.5	8.7
MED15	mediator complex subunit 15	222175_s_at	1.981	0.002	407	790	1.9	1.2	-2.0
ASB9	ankyrin repeat and SOCS box containing 9	205673_s_at	1.978	0.002	89	271	3.1	-1.8	1.6
SEMA3B	semaphorin 3B	203071_at	1.976	0.002	128	268	2.1	1.3	7.8

MICAL1	microtubule associated monooxygenase, calponin and LIM domain containing 1	218376_s_at	1.975	0.002	300	508	1.7	1.8	-23.9
GPRC5A	G protein-coupled receptor class C group 5 member A	203108_at	1.974	0.002	91	194	2.1	1.0	-2.4
IGSF11	immunoglobulin superfamily member 11	228375_at	1.971	0.002	148	342	2.3	-3.0	-15.6
CDK2	cyclin dependent kinase 2	204252_at	1.970	0.002	934	1644	1.8	1.6	-3.6
SCML4	Scm polycomb group protein like 4	1569225_a_at	1.968	0.002	60	248	4.1	4.4	ND
PCBP4	poly(rC) binding protein 4	209361_s_at	1.956	0.002	220	347	1.6	1.1	1.2
CDH19	cadherin 19	206898_at	1.955	0.002	525	1187	2.3	-1.2	1.0
IGKC	immunoglobulin kappa constant	214669_x_at	1.951	0.002	1083	2813	2.6	8.4	1.1
QDPR	quinoid dihydropteridine reductase	209123_at	1.950	0.002	1100	2066	1.9	1.7	-3.9
LAMA4	laminin subunit alpha 4	202202_s_at	1.944	0.002	580	1113	1.9	1.3	9.4
GYG2	glycogenin 2	210964_s_at	1.942	0.002	219	427	1.9	-2.1	-3.6
KRT18	keratin 18	201596_x_at	1.936	0.002	214	415	1.9	1.6	10.1
CPN1	carboxypeptidase N subunit 1	206256_at	1.936	0.002	117	228	2.0	1.0	1.4
CALU	calumenin	200756_x_at	1.935	0.002	566	880	1.6	1.7	2.8
FARP1	FERM, ARH/RhoGEF and pleckstrin domain protein 1	201911_s_at	1.931	0.002	372	632	1.7	1.1	-1.8
GPX1	glutathione peroxidase 1	200736_s_at	1.926	0.002	1962	3312	1.7	1.7	3.0
RENBP	renin binding protein	206617_s_at	1.925	0.002	251	456	1.8	1.1	-2.7
SERPINA5	serpin family A member 5	209443_at	1.922	0.002	72	178	2.5	1.1	2.8
PHC2	polyhomeotic homolog 2	200919_at	1.918	0.002	703	1275	1.8	1.3	1.8
DCPS	decapping enzyme, scavenger	218774_at	1.916	0.002	241	374	1.6	1.2	2.1
TMEM201	transmembrane protein 201	227617_at	1.914	0.002	304	467	1.5	1.0	1.1
TBXA2R	thromboxane A2 receptor	336_at	1.912	0.002	102	205	2.0	1.1	1.7
NUP210	nucleoporin 210	213947_s_at	1.908	0.002	222	397	1.8	1.8	1.1
TPST1	tyrosylprotein sulfotransferase 1	204140_at	1.908	0.002	180	361	2.0	1.8	3.0
TGFB1I1	transforming growth factor beta 1 induced transcript 1	209651_at	1.905	0.002	462	751	1.6	-1.4	-4.3
GSTM4	glutathione S-transferase mu 4	204149_s_at	1.904	0.002	129	232	1.8	1.3	-2.1

ENPP1	ectonucleotide pyrophosphatase/phosphodiesterase 1	205066_s_at	1.901	0.002	118	234	2.0	1.4	1.1
CYREN	cell cycle regulator of NHEJ	220949_s_at	1.898	0.002	314	474	1.5	1.0	2.4
FNDC3B	fibronectin type III domain containing 3B	222692_s_at	1.894	0.002	155	269	1.7	1.2	2.6
TSPAN14	tetraspanin 14	223314_at	1.894	0.002	317	512	1.6	-1.1	2.4
SPRY1	sprouty RTK signaling antagonist 1	212558_at	1.891	0.002	478	768	1.6	-1.1	1.5
TNS2	tensin 2	212494_at	1.888	0.002	244	371	1.5	1.0	1.0
TNFRSF12A	TNF receptor superfamily member 12A	218368_s_at	1.875	0.002	259	444	1.7	1.4	4.2
MAP3K12	mitogen-activated protein kinase kinase kinase 12	205447_s_at	1.872	0.002	166	270	1.6	1.5	-3.8
SLC39A14	solute carrier family 39 member 14	212110_at	1.871	0.002	496	870	1.8	1.5	2.5
CTXND1	cortexin domain containing 1	215126_at	1.863	0.002	194	535	2.8	-1.9	1.2
RPP25	ribonuclease P and MRP subunit p25	219143_s_at	1.857	0.002	177	316	1.8	1.3	1.1
CDKN2C	cyclin dependent kinase inhibitor 2C	204159_at	1.855	0.002	358	639	1.8	1.5	1.4
STMN3	stathmin 3	222557_at	1.852	0.002	294	479	1.6	1.1	3.4
TSPAN4	tetraspanin 4	209264_s_at	1.841	0.002	382	621	1.6	1.0	1.3
CENPF	centromere protein F	207828_s_at	1.834	0.002	327	523	1.6	3.0	4.5
ASRGL1	asparaginase like 1	222764_at	1.832	0.002	98	260	2.7	2.2	1.4
SNHG17	small nucleolar RNA host gene 17	225857_s_at	1.831	0.002	479	723	1.5	1.3	2.1
RUBCNL	rubicon like autophagy enhancer	219471_at	1.831	0.002	231	413	1.8	1.8	1.2
SPRY4-IT1	SPRY4 intronic transcript 1	1566968_at	1.821	0.002	271	571	2.1	1.9	ND
STAG3L4	stromal antigen 3-like 4 (pseudogene)	222801_s_at	1.817	0.002	139	254	1.8	1.0	-1.2
PIR	pirin	207469_s_at	1.811	0.002	607	947	1.6	-1.7	-10.4
COMMD4	COMM domain containing 4	209132_s_at	1.810	0.002	444	675	1.5	1.2	-1.5
DCBLD2	discoidin, CUB and LCCL domain containing 2	224911_s_at	1.809	0.002	283	612	2.2	1.7	2.4
BGN	biglycan	213905_x_at	1.808	0.002	365	589	1.6	1.2	2.3
PABPC1L	poly(A) binding protein cytoplasmic 1 like	226670_s_at	1.802	0.002	325	609	1.9	3.0	1.0
RPS6KA2	ribosomal protein S6 kinase A2	212912_at	1.800	0.002	304	562	1.9	1.4	-9.8

PTPN1	protein tyrosine phosphatase non-receptor type 1	202716_at	1.800	0.002	271	493	1.8	1.2	-1.5
AMPD2	adenosine monophosphate deaminase 2	212360_at	1.789	0.002	295	469	1.6	1.1	1.4
AP1S2	adaptor related protein complex 1 subunit sigma 2	230264_s_at	1.787	0.002	1687	2600	1.5	1.7	-2.2
ADAM12	ADAM metallopeptidase domain 12	202952_s_at	1.783	0.002	108	247	2.3	2.7	4.1
GPR161	G protein-coupled receptor 161	214104_at	1.782	0.002	184	328	1.8	1.7	-1.6
CPOX	coproporphyrinogen oxidase	204172_at	1.781	0.002	220	355	1.6	1.0	1.4
SYNGR1	synaptogyrin 1	210613_s_at	1.781	0.002	505	778	1.5	-1.1	-6.5
RXRG	retinoid X receptor gamma	205954_at	1.780	0.002	82	195	2.4	-2.0	-1.2
RPS10	ribosomal protein S10	214001_x_at	1.775	0.002	243	406	1.7	-2.5	1.0
DTYMK	deoxythymidylate kinase	1553984_s_at	1.766	0.002	434	667	1.5	1.7	ND
NEDD9	neural precursor cell expressed, developmentally down-regulated 9	202149_at	1.766	0.002	330	518	1.6	1.1	1.4
COL12A1	collagen type XII alpha 1 chain	225664_at	1.765	0.002	919	1508	1.6	1.6	10.4
PXDN	peroxidasin	212013_at	1.764	0.002	102	258	2.5	1.8	13.3
TYW3	tRNA-yW synthesizing protein 3 homolog	227141_at	1.762	0.002	305	490	1.6	1.2	2.0
LINC00622	long intergenic non-protein coding RNA 622	1558404_at	1.761	0.002	96	218	2.3	2.9	ND
ITPK1	inositol-tetrakisphosphate 1-kinase	210740_s_at	1.759	0.002	348	532	1.5	-1.1	1.2
CRELD2	cysteine rich with EGF like domains 2	218358_at	1.754	0.002	816	1736	2.1	1.9	1.9
COL11A1	collagen type XI alpha 1 chain	37892_at	1.752	0.002	171	349	2.0	1.8	-1.8
CTSB	cathepsin B	200838_at	1.751	0.002	4037	6115	1.5	2.1	1.0
FOXM1	forkhead box M1	202580_x_at	1.748	0.002	103	209	2.0	2.2	6.7
HTRA1	HtrA serine peptidase 1	201185_at	1.746	0.002	1139	1797	1.6	1.2	-1.4
BMP8B	bone morphogenetic protein 8b	235275_at	1.742	0.002	138	399	2.9	2.0	6.7
NDUFAF3	NADH:ubiquinone oxidoreductase complex assembly factor 3	209177_at	1.741	0.002	385	634	1.6	1.1	-1.3
CDCA3	cell division cycle associated 3	223307_at	1.738	0.002	187	305	1.6	1.8	4.6
ST3GAL5	ST3 beta-galactoside alpha-2,3-sialyltransferase 5	203217_s_at	1.737	0.002	685	1137	1.7	1.1	-2.2
TMEM255A	transmembrane protein 255A	219895_at	1.736	0.002	103	296	2.9	1.7	1.4

GREB1	growth regulating estrogen receptor binding 1	205862_at	1.735	0.002	128	322	2.5	1.2	-5.0
COL5A3	collagen type V alpha 3 chain	52255_s_at	1.732	0.002	256	394	1.5	1.2	1.5
MLXIP	MLX interacting protein	202519_at	1.725	0.002	420	700	1.7	1.3	-2.1
PRC1	protein regulator of cytokinesis 1	218009_s_at	1.719	0.002	302	494	1.6	2.0	15.8
GNS	glucosamine (N-acetyl)-6-sulfatase	212334_at	1.719	0.002	1507	2374	1.6	1.3	-1.5
ISYNA1	inositol-3-phosphate synthase 1	222240_s_at	1.716	0.002	161	264	1.6	1.1	1.0
TIMP2	TIMP metallopeptidase inhibitor 2	203167_at	1.714	0.002	605	995	1.6	1.6	-4.8
HTRA2	HtrA serine peptidase 2	203089_s_at	1.714	0.002	417	664	1.6	1.2	1.5
ST3GAL4	ST3 beta-galactoside alpha-2,3-sialyltransferase 4	203759_at	1.712	0.002	196	349	1.8	-1.3	1.0
CENPN	centromere protein N	228559_at	1.711	0.002	170	272	1.6	1.8	1.9
CLCC1	chloride channel CLIC like 1	213628_at	1.710	0.002	212	327	1.5	1.0	1.4
LRMDA	leucine rich melanocyte differentiation associated	223703_at	1.704	0.002	194	309	1.6	-1.2	-3.9
FMN1	formin 1	238621_at	1.701	0.002	240	442	1.8	1.0	-3.9
VEGFB	vascular endothelial growth factor B	203683_s_at	1.698	0.002	223	337	1.5	-1.1	-3.3
CORO2B	coronin 2B	209789_at	1.692	0.002	158	352	2.2	-4.7	-1.1
LINC00888	long intergenic non-protein coding RNA 888	236798_at	1.691	0.002	135	276	2.0	1.6	1.0
CDC20	cell division cycle 20	202870_s_at	1.691	0.002	185	333	1.8	2.3	26.7
PDE3B	phosphodiesterase 3B	214582_at	1.689	0.002	149	323	2.2	1.6	-2.4
COL15A1	collagen type XV alpha 1 chain	203477_at	1.688	0.002	1607	2653	1.7	2.1	8.6
DAG1	dystroglycan 1	205417_s_at	1.686	0.002	894	1376	1.5	1.0	1.8
BCAR3	BCAR3 adaptor protein, NSP family member	204032_at	1.682	0.002	126	306	2.4	1.0	1.0
PLXND1	plexin D1	38671_at	1.681	0.002	638	1011	1.6	1.2	-1.2
MPV17	mitochondrial inner membrane protein MPV17	203466_at	1.680	0.002	423	653	1.5	1.1	-1.9
GDF11	growth differentiation factor 11	226234_at	1.677	0.002	89	204	2.3	1.2	-1.8
S100A13	S100 calcium binding protein A13	202598_at	1.672	0.002	1283	2013	1.6	1.3	-1.8
HPCAL1	hippocalcin like 1	212552_at	1.665	0.002	828	1430	1.7	1.4	1.3
HMGA2	high mobility group AT-hook 2	208025_s_at	1.663	0.002	50	173	3.4	2.0	11.5

IFFO1	intermediate filament family orphan 1	209721_s_at	1.663	0.002	334	515	1.5	1.1	-1.2
BST2	bone marrow stromal cell antigen 2	201641_at	1.663	0.002	439	896	2.0	2.7	2.0
MAPK12	mitogen-activated protein kinase 12	206106_at	1.660	0.002	103	290	2.8	1.7	1.6
SLC2A10	solute carrier family 2 member 10	221024_s_at	1.656	0.002	211	347	1.6	1.8	-1.7
STAB1	stabilin 1	38487_at	1.652	0.002	618	984	1.6	1.7	-1.1
PBK	PDZ binding kinase	219148_at	1.652	0.002	183	320	1.7	5.2	11.7
TMEM51	transmembrane protein 51	218815_s_at	1.650	0.002	197	324	1.6	1.1	-2.9
HEXA	hexosaminidase subunit alpha	201765_s_at	1.640	0.002	878	1390	1.6	1.3	-4.6
IGLJ3	immunoglobulin lambda joining 3	216984_x_at	1.639	0.002	171	543	3.2	2.2	1.0
NRROS	negative regulator of reactive oxygen species	235359_at	1.638	0.002	148	276	1.9	-1.1	-1.1
CMTM5	CKLF like MARVEL transmembrane domain containing 5	230942_at	1.637	0.002	107	218	2.0	-1.4	-1.4
SLC16A4	solute carrier family 16 member 4	205234_at	1.634	0.002	137	273	2.0	-1.2	1.6
CCND1	cyclin D1	208711_s_at	1.628	0.003	681	1096	1.6	1.1	5.1
TMEM223	transmembrane protein 223	220934_s_at	1.625	0.003	229	343	1.5	-1.2	1.5
SEMA5A	semaphorin 5A	229427_at	1.624	0.003	232	374	1.6	-1.1	-2.0
LOXL2	lysyl oxidase like 2	202998_s_at	1.622	0.003	332	536	1.6	2.1	39.2
GUSBP11	GUSB pseudogene 11	213502_x_at	1.621	0.003	600	1233	2.1	2.5	-1.6
MCM5	minichromosome maintenance complex component 5	216237_s_at	1.621	0.003	321	500	1.6	1.3	7.4
IGHD	immunoglobulin heavy constant delta	214973_x_at	1.619	0.003	161	528	3.3	1.9	1.2
LMF1	lipase maturation factor 1	1569872_a_at	1.608	0.003	242	421	1.7	-1.3	ND
MBP	myelin basic protein	209072_at	1.607	0.003	600	1054	1.8	-1.1	-18.9
IGF2R	insulin like growth factor 2 receptor	201392_s_at	1.607	0.003	364	563	1.5	1.2	1.0
SOX8	SRY-box 8	226913_s_at	1.606	0.003	121	247	2.0	1.3	3.3
SNCA	synuclein alpha	204466_s_at	1.601	0.003	1352	2027	1.5	-1.1	-9.5
HIP1	huntingtin interacting protein 1	226364_at	1.599	0.003	278	472	1.7	1.0	1.9
PWAR6	Prader Willi/Angelman region RNA 6	226587_at	1.596	0.003	212	341	1.6	2.5	-1.1
GNA14	G protein subunit alpha 14	220108_at	1.590	0.003	95	201	2.1	1.4	1.4
SLC20A1	solute carrier family 20 member 1	201920_at	1.586	0.003	854	1780	2.1	4.6	14.9
CFI	complement factor I	203854_at	1.586	0.003	143	249	1.7	2.7	2.4

SLC2A3	solute carrier family 2 member 3	202497_x_at	1.581	0.003	408	770	1.9	2.2	1.5
MMP3	matrix metallopeptidase 3	205828_at	1.575	0.003	156	510	3.3	5.8	1.5
MBOAT1	membrane bound O-acyltransferase domain containing 1	227379_at	1.572	0.003	136	259	1.9	1.1	1.0
TGFBI	transforming growth factor beta induced	201506_at	1.563	0.003	1601	2596	1.6	-1.5	49.5
RRM2	ribonucleotide reductase regulatory subunit M2	209773_s_at	1.563	0.003	439	687	1.6	4.1	30.9
GAPDHS	glyceraldehyde-3-phosphate dehydrogenase, spermatogenic	207116_s_at	1.562	0.003	189	559	3.0	-2.6	-1.8
PSMA7	proteasome subunit alpha 7	216088_s_at	1.561	0.003	295	469	1.6	2.2	1.3
SPRY2	sprouty RTK signaling antagonist 2	204011_at	1.557	0.003	382	805	2.1	-1.4	7.2
PRSS23	serine protease 23	202458_at	1.553	0.003	688	1054	1.5	-1.3	17.0
CCN1	cellular communication network factor 1	210764_s_at	1.553	0.003	288	501	1.7	2.5	7.6
APLNR	apelin receptor	213592_at	1.552	0.003	284	442	1.6	1.6	-1.2
CPEB1	cytoplasmic polyadenylation element binding protein 1	219578_s_at	1.544	0.003	186	294	1.6	1.0	-3.4
LZTS1	leucine zipper tumor suppressor 1	219042_at	1.543	0.003	214	345	1.6	-2.5	-4.5
FDCSP	follicular dendritic cell secreted protein	229152_at	1.540	0.003	55	208	3.8	2.9	1.5
EHBP1	EH domain binding protein 1	212650_at	1.540	0.003	261	422	1.6	1.8	1.0
RFC4	replication factor C subunit 4	204023_at	1.539	0.003	267	506	1.9	2.0	5.0
CXCL1	C-X-C motif chemokine ligand 1	204470_at	1.537	0.003	145	454	3.1	4.4	2.2
MFF	mitochondrial fission factor	222832_s_at	1.537	0.003	712	1090	1.5	-1.2	2.0
PLEKHA2	pleckstrin homology domain containing A2	238013_at	1.535	0.003	144	259	1.8	1.0	-1.5
OLFML2B	olfactomedin like 2B	213125_at	1.533	0.003	284	483	1.7	2.8	-1.2
EFEMP2	EGF containing fibulin extracellular matrix protein 2	209356_x_at	1.530	0.003	256	418	1.6	1.0	1.1
GALE	UDP-galactose-4-epimerase	202528_at	1.530	0.003	177	315	1.8	1.0	2.3
ST6GAL1	ST6 beta-galactoside alpha-2,6-sialyltransferase 1	201998_at	1.519	0.003	506	759	1.5	-1.2	-1.6

PLEKHO2	pleckstrin homology domain containing O2	204436_at	1.518	0.003	473	720	1.5	1.1	-1.6
COTL1	coactosin like F-actin binding protein 1	224583_at	1.515	0.003	742	1167	1.6	2.3	13.7
CDCA8	cell division cycle associated 8	221520_s_at	1.513	0.003	194	303	1.6	1.3	3.7
SULF1	sulfatase 1	212354_at	1.510	0.003	281	492	1.8	3.2	3.9
CCNB2	cyclin B2	202705_at	1.497	0.003	282	467	1.7	2.3	5.1
GUSB	glucuronidase beta	202605_at	1.493	0.003	956	1535	1.6	1.5	1.0
EMILIN2	elastin microfibril interfacer 2	224374_s_at	1.492	0.003	382	670	1.8	1.9	1.2
CARD16	caspase recruitment domain family member 16	1552701_a_at	1.491	0.003	331	632	1.9	3.8	ND
TNC	tenascin C	201645_at	1.488	0.003	1163	2188	1.9	3.6	37.6
BUB1B	BUB1 mitotic checkpoint serine/threonine kinase B	203755_at	1.486	0.003	180	335	1.9	2.4	4.4
BANCR	BRAF-activated non-protein coding RNA	239239_at	1.486	0.003	115	240	2.1	2.4	1.2
EGFR	epidermal growth factor receptor	1565484_x_at	1.486	0.003	318	532	1.7	1.1	ND
PKN1	protein kinase N1	202161_at	1.481	0.003	170	271	1.6	1.1	1.5
KCTD7	potassium channel tetramerization domain containing 7	213474_at	1.481	0.003	212	326	1.5	1.3	-2.0
TCN1	transcobalamin 1	205513_at	1.468	0.003	387	1250	3.2	8.7	-1.3
MAD2L2	mitotic arrest deficient 2 like 2	223234_at	1.464	0.003	359	723	2.0	1.6	1.8
KCNJ10	potassium voltage-gated channel subfamily J member 10	228581_at	1.460	0.003	76	195	2.6	2.3	1.2
AOC1	amine oxidase copper containing 1	203559_s_at	1.458	0.003	61	169	2.8	1.8	-1.1
NELL1	neural EGFL like 1	206089_at	1.453	0.003	78	215	2.8	1.5	1.0
IGLV2-14	immunoglobulin lambda variable 2-14	217148_x_at	1.448	0.003	338	1283	3.8	2.6	1.0
MMP11	matrix metallopeptidase 11	203878_s_at	1.442	0.003	242	389	1.6	1.6	-1.1
L1CAM	L1 cell adhesion molecule	204584_at	1.439	0.003	239	935	3.9	2.3	-3.3
NNMT	nicotinamide N-methyltransferase	202238_s_at	1.436	0.003	496	920	1.9	4.0	2.2
TDO2	tryptophan 2,3-dioxygenase	205943_at	1.435	0.003	99	216	2.2	5.9	1.1
CADPS	calcium dependent secretion activator	1568604_a_at	1.432	0.003	160	324	2.0	3.8	ND

C3orf70	chromosome 3 open reading frame 70	242447_at	1.425	0.003	217	357	1.6	-2.1	1.4
LINC-PINT	long intergenic non-protein coding RNA, p53 induced transcript	228702_at	1.424	0.003	138	247	1.8	1.4	-1.6
RNASE1	ribonuclease A family member 1, pancreatic	201785_at	1.413	0.003	2729	4430	1.6	2.9	1.2
NPTX2	neuronal pentraxin 2	213479_at	1.413	0.003	161	393	2.4	-1.4	4.6
PEG10	paternally expressed 10	212094_at	1.408	0.003	387	788	2.0	-1.5	4.0
ANO4	anoctamin 4	236420_s_at	1.401	0.003	54	260	4.9	4.4	3.6
PLPP5	phospholipid phosphatase 5	226150_at	1.401	0.003	172	311	1.8	2.4	1.6
SCUBE2	signal peptide, CUB domain and EGF like domain containing 2	219197_s_at	1.398	0.003	325	614	1.9	1.0	-2.7
CNIH3	cornichon family AMPA receptor auxiliary protein 3	214841_at	1.392	0.003	152	394	2.6	3.8	1.0
NT5E	5'-nucleotidase ecto	1553995_a_at	1.390	0.003	54	201	3.8	2.5	ND
FANCI	FA complementation group I	213007_at	1.390	0.003	163	305	1.9	1.9	2.4
GLB1L	galactosidase beta 1 like	206540_at	1.382	0.003	174	332	1.9	-1.1	1.1
FGF13	fibroblast growth factor 13	205110_s_at	1.378	0.003	150	253	1.7	2.9	4.3
PLAAT3	phospholipase A and acyltransferase 3	209581_at	1.372	0.003	323	487	1.5	2.9	-1.2
CHAD	chondroadherin	206869_at	1.371	0.003	133	255	1.9	1.0	1.0
FRMD5	FERM domain containing 5	230831_at	1.363	0.003	50	162	3.2	2.0	2.5
PLEKHH1	pleckstrin homology, MyTH4 and FERM domain containing H1	225726_s_at	1.361	0.003	465	702	1.5	1.4	-1.9
ARAP3	ArfGAP with RhoGAP domain, ankyrin repeat and PH domain 3	218950_at	1.361	0.003	204	344	1.7	1.1	-1.1
C4orf48	chromosome 4 open reading frame 48	229860_x_at	1.358	0.003	343	521	1.5	1.3	-2.5
SPRED2	sprouty related EVH1 domain containing 2	212458_at	1.356	0.003	304	481	1.6	-1.1	4.7
LAMA1	laminin subunit alpha 1	227048_at	1.354	0.003	148	293	2.0	5.8	1.7
NOL4L	nucleolar protein 4 like	225224_at	1.351	0.003	295	475	1.6	1.7	1.3
TRPV2	transient receptor potential cation channel subfamily V member 2	219282_s_at	1.349	0.003	525	790	1.5	1.6	-2.2

COL1A1	collagen type I alpha 1 chain	202311_s_at	1.341	0.004	1103	1692	1.5	1.7	1.1
EVA1A	eva-1 homolog A, regulator of programmed cell death	227828_s_at	1.330	0.004	174	322	1.9	-1.5	-2.8
IGKV4-1	immunoglobulin kappa variable 4-1	214777_at	1.325	0.004	173	570	3.3	3.8	1.0
FAM20C	FAM20C golgi associated secretory pathway kinase	226722_at	1.317	0.004	237	388	1.6	1.6	12.6
MICALL2	MICAL like 2	219332_at	1.316	0.004	176	286	1.6	1.8	1.6
SLCO4A1-AS1	SLCO4A1 antisense RNA 1	1554332_a_at	1.310	0.004	294	471	1.6	-2.2	ND
VEPH1	ventricular zone expressed PH domain containing 1	232122_s_at	1.310	0.004	283	463	1.6	-1.5	-27.6
NPL	N-acetylneuraminate pyruvate lyase	240440_at	1.309	0.004	148	252	1.7	3.8	-2.3
TNIK	TRAF2 and NCK interacting kinase	211828_s_at	1.308	0.004	87	231	2.7	2.0	-1.2
IGHM	immunoglobulin heavy constant mu	216491_x_at	1.307	0.004	145	499	3.4	3.5	1.0
IGK	immunoglobulin kappa locus	211650_x_at	1.303	0.004	120	227	1.9	1.7	1.1
PIEZO2	piezo type mechanosensitive ion channel component 2	219602_s_at	1.302	0.004	128	259	2.0	2.4	1.4
TTYH3	tweety family member 3	224674_at	1.299	0.004	884	1334	1.5	1.8	-1.2
LPXN	leupaxin	216250_s_at	1.296	0.004	533	909	1.7	3.2	3.5
THBS1	thrombospondin 1	201108_s_at	1.285	0.004	224	427	1.9	2.3	22.1
SAP30	Sin3A associated protein 30	204900_x_at	1.282	0.004	269	498	1.8	-1.4	-1.1
TMTC2	transmembrane and tetratricopeptide repeat containing 2	235775_at	1.282	0.004	193	295	1.5	-1.3	2.0
POMGNT1	protein O-linked mannose N-acetylglucosaminyltransferase 1 (beta 1,2-)	217944_at	1.278	0.004	319	486	1.5	-1.2	-1.1
HLA-DRB4	major histocompatibility complex, class II, DR beta 4	209728_at	1.277	0.004	590	1160	2.0	1.8	7.1
SLC23A2	solute carrier family 23 member 2	209236_at	1.269	0.004	419	630	1.5	-1.3	-1.6
TCIRG1	T cell immune regulator 1, ATPase H+ transporting V0 subunit a3	204158_s_at	1.265	0.004	236	412	1.7	2.2	1.4
SSH1	slingshot protein phosphatase 1	221752_at	1.259	0.004	323	493	1.5	-1.1	1.9
DMXL2	Dmx like 2	212820_at	1.257	0.004	254	389	1.5	2.0	-2.1
SMIM3	small integral membrane protein 3	223276_at	1.256	0.004	459	788	1.7	1.3	2.8

LARP6	La ribonucleoprotein domain family member 6	236565_s_at	1.254	0.004	197	316	1.6	1.4	1.6
KIAA1549L	KIAA1549 like	235486_at	1.253	0.004	227	380	1.7	-2.0	1.2
ARHGAP18	Rho GTPase activating protein 18	225173_at	1.252	0.004	191	292	1.5	2.3	1.9
PDGFA	platelet derived growth factor subunit A	205463_s_at	1.252	0.004	201	311	1.5	-1.2	8.9
PSPH	phosphoserine phosphatase	205048_s_at	1.251	0.004	234	385	1.6	2.5	3.8
IGLL5	immunoglobulin lambda like polypeptide 5	234764_x_at	1.248	0.004	89	452	5.1	5.4	-1.2
NHLRC3	NHL repeat containing 3	227040_at	1.243	0.004	212	338	1.6	1.5	-2.6
ASL	argininosuccinate lyase	204608_at	1.237	0.004	259	393	1.5	1.5	1.4
EYA1	EYA transcriptional coactivator and phosphatase 1	214608_s_at	1.237	0.004	110	211	1.9	2.2	1.2
LDLRAD3	low density lipoprotein receptor class A domain containing 3	234985_at	1.234	0.004	584	965	1.7	-1.6	2.7
TMED4	transmembrane p24 trafficking protein 4	224680_at	1.232	0.004	312	503	1.6	-1.2	1.4
LOXL4	lysyl oxidase like 4	227145_at	1.231	0.004	558	1187	2.1	1.0	31.0
IFI35	interferon induced protein 35	209417_s_at	1.222	0.004	304	469	1.5	1.9	1.6
SGCE	sarcoglycan epsilon	204688_at	1.221	0.004	284	444	1.6	-1.4	1.6
GLT8D1	glycosyltransferase 8 domain containing 1	218147_s_at	1.214	0.004	246	409	1.7	1.2	-1.2
PLCB4	phospholipase C beta 4	203895_at	1.209	0.004	103	275	2.7	2.8	1.5
ATP8B2	ATPase phospholipid transporting 8B2	226771_at	1.209	0.004	514	781	1.5	-1.4	1.0
TRIM2	tripartite motif containing 2	202341_s_at	1.204	0.004	202	309	1.5	-1.3	-3.7
FAM92A	family with sequence similarity 92 member A	235391_at	1.201	0.004	142	258	1.8	1.9	1.1
IGF2BP2	insulin like growth factor 2 mRNA binding protein 2	218847_at	1.200	0.004	283	550	1.9	-1.1	1.9
CCN2	cellular communication network factor 2	209101_at	1.191	0.004	562	1054	1.9	1.8	43.5
AKAP12	A-kinase anchoring protein 12	227530_at	1.186	0.004	255	388	1.5	-1.7	1.5
RGS3	regulator of G protein signaling 3	203823_at	1.184	0.004	240	395	1.6	-1.1	1.3

IER3	immediate early response 3	201631_s_at	1.174	0.005	1279	2009	1.6	2.2	12.6
TMEM35B	transmembrane protein 35B	213698_at	1.164	0.005	227	350	1.5	1.3	1.8
NTRK3	neurotrophic receptor tyrosine kinase 3	215311_at	1.161	0.005	38	285	7.5	3.6	-1.3
ACOT7	acyl-CoA thioesterase 7	208002_s_at	1.159	0.005	547	853	1.6	1.7	2.2
EFR3B	EFR3 homolog B	227283_at	1.158	0.005	192	296	1.5	-1.1	-1.1
ANLN	anillin actin binding protein	222608_s_at	1.151	0.005	156	260	1.7	3.3	26.8
SORL1	sortilin related receptor 1	212560_at	1.149	0.005	352	540	1.5	1.5	1.6
TNNC2	troponin C2, fast skeletal type	205388_at	1.147	0.005	175	378	2.2	3.2	1.0
MYOC	myocilin	210155_at	1.147	0.005	152	260	1.7	1.1	1.1
SLC25A13	solute carrier family 25 member 13	229061_s_at	1.146	0.005	274	424	1.5	2.6	1.1
CLEC11A	C-type lectin domain containing 11A	211709_s_at	1.132	0.005	246	376	1.5	1.3	-2.1
CAPN3	calpain 3	210944_s_at	1.129	0.005	980	1477	1.5	-1.1	-54.6
BAMBI	BMP and activin membrane bound inhibitor	203304_at	1.123	0.005	699	1255	1.8	-1.2	5.0
ALDH1A3	aldehyde dehydrogenase 1 family member A3	203180_at	1.122	0.005	586	990	1.7	1.3	45.7
PTPRG	protein tyrosine phosphatase receptor type G	204944_at	1.120	0.005	172	346	2.0	1.8	1.1
NGFR	nerve growth factor receptor	205858_at	1.116	0.005	91	218	2.4	1.5	3.4
PRKD1	protein kinase D1	205880_at	1.113	0.005	138	285	2.1	-1.1	-1.1
BIRC5	baculoviral IAP repeat containing 5	202095_s_at	1.110	0.005	204	327	1.6	2.3	16.8
SHC4	SHC adaptor protein 4	235238_at	1.105	0.005	168	333	2.0	2.0	-2.0
PLAUR	plasminogen activator, urokinase receptor	211924_s_at	1.104	0.005	373	571	1.5	2.4	4.5
THBS4	thrombospondin 4	204776_at	1.099	0.005	204	420	2.1	1.5	1.0
IGKV1OR2-10	immunoglobulin kappa variable 1/OR2-108 (non-functional)	217378_x_at	1.094	0.005	323	876	2.7	5.2	1.0
LRRN4CL	LRRN4 C-terminal like	1556427_s_at	1.094	0.005	476	897	1.9	-1.6	ND
CITED1	Cbp/p300 interacting transactivator with Glu/Asp rich carboxy-terminal domain 1	207144_s_at	1.094	0.005	144	260	1.8	1.6	-7.4
MCOLN3	mucolipin 3	242308_at	1.089	0.005	383	615	1.6	-1.3	-1.1

IGKV1D-13	immunoglobulin kappa variable 1D-13	216207_x_at	1.078	0.005	512	1676	3.3	4.6	1.1
ADIPOQ	adiponectin, C1Q and collagen domain containing	207175_at	1.070	0.005	93	287	3.1	4.2	1.0
HAGLR	HOXD antisense growth-associated long non-coding RNA	228601_at	1.068	0.005	125	228	1.8	1.7	1.5
PTPRM	protein tyrosine phosphatase receptor type M	1555579_s_at	1.066	0.005	735	1123	1.5	-1.3	ND
COMP	cartilage oligomeric matrix protein	205713_s_at	1.063	0.005	310	627	2.0	1.3	1.1
BAALC	BAALC binder of MAP3K1 and KLF4	218899_s_at	1.062	0.005	148	568	3.8	1.7	2.0
ISG20	interferon stimulated exonuclease gene 20	204698_at	1.048	0.005	257	430	1.7	3.4	1.2
ETV1	ETS variant 1	221911_at	1.036	0.005	310	532	1.7	-1.7	8.8
APOC1	apolipoprotein C1	204416_x_at	1.023	0.005	1234	1945	1.6	1.6	-1.1
FOXD3	forkhead box D3	241612_at	1.022	0.005	61	224	3.7	1.4	1.2
IGHV1-69	immunoglobulin heavy variable 1-69	211635_x_at	1.019	0.005	183	600	3.3	2.8	1.0
IFNAR2	interferon alpha and beta receptor subunit 2	227125_at	1.018	0.005	181	286	1.6	1.8	1.4
DOCK10	dedicator of cytokinesis 10	219279_at	1.017	0.005	260	394	1.5	1.1	-1.1
LIMD1	LIM domains containing 1	222762_x_at	1.016	0.005	287	435	1.5	1.2	1.3
IGKV1-17	immunoglobulin kappa variable 1-17	211645_x_at	1.008	0.005	211	816	3.9	8.2	1.0
RPS11	ribosomal protein S11	213350_at	1.007	0.005	639	1040	1.6	-4.7	1.3
LINC00520	long intergenic non-protein coding RNA 520	1555786_s_at	1.002	0.006	1092	1661	1.5	2.3	ND
AP3D1	adaptor related protein complex 3 subunit delta 1	208710_s_at	0.994	0.006	389	822	2.1	1.5	-2.1
GJC1	gap junction protein gamma 1	228776_at	0.971	0.006	240	365	1.5	-1.6	2.8
KAT2B	lysine acetyltransferase 2B	203845_at	0.965	0.006	474	819	1.7	-1.2	-3.1
MMP9	matrix metallopeptidase 9	203936_s_at	0.959	0.006	746	1477	2.0	5.2	1.6
KCNN4	potassium calcium-activated channel subfamily N member 4	204401_at	0.957	0.006	320	585	1.8	1.5	5.4
NEDD4L	NEDD4 like E3 ubiquitin protein ligase	241396_at	0.954	0.006	104	224	2.2	2.7	-2.2

HES1	hes family bHLH transcription factor 1	203394_s_at	0.939	0.006	360	621	1.7	-1.1	1.8
PLIN1	perilipin 1	205913_at	0.934	0.006	227	415	1.8	1.8	1.1
CTSZ	cathepsin Z	210042_s_at	0.925	0.006	364	916	2.5	2.7	-2.8
CRTAC1	cartilage acidic protein 1	221204_s_at	0.922	0.006	230	389	1.7	-5.6	-1.2
C5AR1	complement C5a receptor 1	220088_at	0.922	0.006	265	462	1.7	5.9	1.0
GREM1	gremlin 1, DAN family BMP antagonist	218468_s_at	0.908	0.006	134	277	2.1	1.9	1.0
RARRES2	retinoic acid receptor responder 2	209496_at	0.901	0.007	292	447	1.5	1.7	-1.1
FAH	fumarylacetoacetate hydrolase	202862_at	0.887	0.007	246	372	1.5	2.1	1.2
TENT5C	terminal nucleotidyltransferase 5C	226811_at	0.884	0.007	163	323	2.0	1.3	2.8
DDX3Y	DEAD-box helicase 3 Y-linked	205001_s_at	0.882	0.007	114	227	2.0	1.5	-1.1
POU2AF1	POU class 2 homeobox associating factor 1	205267_at	0.873	0.007	59	169	2.9	2.8	1.0
NTM	neurotrimin	227566_at	0.869	0.007	179	365	2.0	-1.1	16.1
ASAP1	ArfGAP with SH3 domain, ankyrin repeat and PH domain 1	236533_at	0.850	0.007	157	264	1.7	1.3	-1.1
IGLV3-10	immunoglobulin lambda variable 3-10	216560_x_at	0.846	0.007	153	347	2.3	2.4	1.3
FNDC1	fibronectin type III domain containing 1	226930_at	0.845	0.007	110	229	2.1	1.6	1.1
CEMIP	cell migration inducing hyaluronidase 1	212942_s_at	0.844	0.007	363	627	1.7	4.1	1.5
TMEM119	transmembrane protein 119	227300_at	0.825	0.007	207	314	1.5	1.5	-1.2
RGS1	regulator of G protein signaling 1	216834_at	0.824	0.007	869	1458	1.7	24.3	1.2
SRSF6	serine and arginine rich splicing factor 6	206108_s_at	0.791	0.008	169	273	1.6	-1.1	-1.5
LAMB1	laminin subunit beta 1	201505_at	0.789	0.008	486	740	1.5	2.1	3.0
IGHG1	immunoglobulin heavy constant gamma 1 (G1m marker)	211633_x_at	0.788	0.008	176	374	2.1	2.2	-1.1
LRRC8C	leucine rich repeat containing 8 VRAC subunit C	223533_at	0.773	0.008	210	320	1.5	1.0	1.2
CADM1	cell adhesion molecule 1	209032_s_at	0.771	0.008	152	325	2.1	-1.8	1.5

HSPA12A	heat shock protein family A (Hsp70) member 12A	214434_at	0.755	0.008	205	369	1.8	-2.6	-1.4
IFI6	interferon alpha inducible protein 6	204415_at	0.729	0.008	628	1310	2.1	2.4	-16.9
G0S2	G0/G1 switch 2	213524_s_at	0.723	0.008	453	709	1.6	7.1	1.1
CAPG	capping actin protein, gelsolin like	201850_at	0.706	0.008	1031	1561	1.5	2.5	-19.2
JCHAIN	joining chain of multimeric IgA and IgM	212592_at	0.676	0.009	153	333	2.2	7.6	1.0
JAK1	Janus kinase 1	1552611_a_at	0.608	0.009	220	332	1.5	-1.4	ND
ADAMDEC1	ADAM like decysin 1	206134_at	0.573	0.010	383	769	2.0	11.8	1.1

Only probesets with unique annotations are shown.

ND, not determined
