# Supplementary material for: Prolyl 4‐hydroxylase subunit alpha 1 (P4HA1) is a biomarker of poor prognosis in primary melanomas, and its depletion inhibits melanoma cell invasion and disrupts tumor blood vessel walls
Source: Mol Oncol. 2020 Feb 28;14(4):742–62. doi: 10.1002/1878-0261.12649 (PMC7138405; doi:10.1002/1878-0261.12649)
Supplement: Supplementary file 18 — Table S3. Significance Analysis of Microarrays (SAM) results of mRNA expression levels in primary melanomas associated most significantly with patient survival (lower expression in cases with short survival). [file MOL2-14-742-s018.pdf]

Table S3. Significance Analysis of Microarrays (SAM) results of mRNA expression levels in primary melanomas associated most significantly with patient survival (lower expression in cases with short survival).

					Mean expression		Fold	Fold	Fold
Gene	Gene description	Gene ID	SAM score	False discovery rate q-value	Alive	Dead	Dead vs alive	Primary melanomas vs benign nevi	Melanoma cells vs melanocytes
KRT10	keratin 10	210633_x_at	-3.327	0.001	10758	6829	-1.6	-1.2	1.2
NCOA4	nuclear receptor coactivator 4	210774_s_at	-3.090	0.001	3899	2582	-1.5	-1.0	-2.8
SCP2	sterol carrier protein 2	211733_x_at	-3.085	0.001	2263	1370	-1.7	1.2	-3.4
FOXN3	forkhead box N3	222494_at	-3.061	0.001	870	495	-1.8	-1.8	-2.3
MTSS1	MTSS I-BAR domain containing 1	203037_s_at	-3.058	0.001	1394	793	-1.8	-1.1	-4.6
ANXA4	annexin A4	201302_at	-2.884	0.001	1261	741	-1.7	-1.3	-5.9
REEP5	receptor accessory protein 5	208873_s_at	-2.870	0.001	971	499	-1.9	-1.1	-1.9
FBXL3	F-box and leucine rich repeat protein 3	225132_at	-2.812	0.001	1402	828	-1.7	-1.2	-1.0
CAST	calpastatin	212586_at	-2.804	0.001	2175	1276	-1.7	-1.3	-3.2
CCDC6	coiled-coil domain containing 6	225010_at	-2.794	0.001	1042	574	-1.8	-1.2	1.0
RAB18	RAB18, member RAS oncogene family	224787_s_at	-2.779	0.001	1174	628	-1.9	1.2	-2.0
DMKN	dermokine	226926_at	-2.778	0.001	4644	1525	-3.0	-1.9	-1.2
SLC38A2	solute carrier family 38 member 2	222982_x_at	-2.758	0.001	3170	1877	-1.7	-1.3	-1.2
SHOC2	SHOC2 leucine rich repeat scaffold protein	202777_at	-2.754	0.001	581	340	-1.7	1.1	-1.7
PNRC2	proline rich nuclear receptor coactivator 2	217779_s_at	-2.736	0.001	1307	853	-1.5	1.1	1.0
HIPK3	homeodomain interacting protein kinase 3	226297_at	-2.736	0.001	1930	1038	-1.9	1.0	-1.0
EGFR	epidermal growth factor receptor	201983_s_at	-2.710	0.001	1503	475	-3.2	-2.0	1.4
RAP2A	RAP2A, member of RAS oncogene family	225585_at	-2.701	0.001	845	434	-1.9	1.4	-3.4
FAM126A	family with sequence similarity 126 member A	227239_at	-2.690	0.001	1102	481	-2.3	1.7	1.2
TUFT1	tuftelin 1	205807_s_at	-2.680	0.001	953	376	-2.5	-1.5	-5.5
DSC3	desmocollin 3	206033_s_at	-2.665	0.001	2461	543	-4.5	-1.8	1.0
PERP	p53 apoptosis effector related to PMP22	222392_x_at	-2.661	0.001	8938	5018	-1.8	-1.3	2.4

SNHG5	small nucleolar RNA host gene 5	225155_at	-2.660	0.001	3572	1767	-2.0	-1.4	2.4
HOPX	HOP homeobox	211597_s_at	-2.649	0.001	5113	1635	-3.1	-1.8	-1.1
GSTA4	glutathione S-transferase alpha 4	202967_at	-2.649	0.001	1543	699	-2.2	-1.5	-5.3
TM9SF3	transmembrane 9 superfamily member 3	224755_at	-2.647	0.001	753	393	-1.9	-1.4	1.1
SLC30A1	solute carrier family 30 member 1	212907_at	-2.643	0.001	1675	703	-2.4	1.6	2.0
CLDN1	claudin 1	222549_at	-2.643	0.001	1713	337	-5.1	-2.1	1.5
PTBP3	polypyrimidine tract binding protein 3	224617_at	-2.640	0.001	1759	996	-1.8	1.0	1.5
CMPK1	cytidine/uridine monophosphate kinase 1	217870_s_at	-2.637	0.001	1488	769	-1.9	1.1	1.1
NR3C1	nuclear receptor subfamily 3 group C member 1	216321_s_at	-2.632	0.001	844	508	-1.7	-1.5	-1.7
RIOK3	RIO kinase 3	202130_at	-2.628	0.001	1205	674	-1.8	1.3	1.7
C11orf58	chromosome 11 open reading frame 58	225811_at	-2.619	0.001	569	339	-1.7	-1.6	-1.2
CXADR	CXADR Ig-like cell adhesion molecule	203917_at	-2.606	0.001	1950	548	-3.6	-1.2	-1.1
SPIN1	spindlin 1	222431_at	-2.606	0.001	999	645	-1.5	-1.2	1.0
KRTDAP	keratinocyte differentiation associated protein	230835_at	-2.605	0.001	11301	5671	-2.0	-1.3	-1.0
ASPRV1	aspartic peptidase retroviral like 1	235514_at	-2.586	0.001	5419	2056	-2.6	-1.1	-1.1
SLK	STE20 like kinase	206874_s_at	-2.577	0.001	545	276	-2.0	-1.2	-2.6
KRT5	keratin 5	201820_at	-2.574	0.001	9290	4739	-2.0	-1.6	1.3
TACSTD2	tumor associated calcium signal transducer 2	202286_s_at	-2.572	0.001	5283	1898	-2.8	-1.5	-1.1
CERS6	ceramide synthase 6	212442_s_at	-2.571	0.001	786	461	-1.7	-1.5	1.9
DDX3X	DEAD-box helicase 3 X-linked	212515_s_at	-2.559	0.001	1064	633	-1.7	1.0	1.1
TOB1	transducer of ERBB2, 1	202704_at	-2.556	0.001	1330	659	-2.0	-1.0	-2.6
TYRP1	tyrosinase related protein 1	205694_at	-2.552	0.001	8111	3204	-2.5	-1.4	-267.4
RNF141	ring finger protein 141	226106_at	-2.545	0.001	984	418	-2.4	-1.3	-1.9
TMEM106B	transmembrane protein 106B	226529_at	-2.537	0.001	1159	546	-2.1	1.1	-1.3
TP63	tumor protein p63	209863_s_at	-2.528	0.001	1485	413	-3.6	-2.0	1.3
PDCD4	programmed cell death 4	212593_s_at	-2.524	0.001	1865	1085	-1.7	-1.4	-1.5
KRT1	keratin 1	205900_at	-2.521	0.001	13309	7534	-1.8	-1.4	1.2
CCNG2	cyclin G2	202769_at	-2.521	0.001	627	345	-1.8	-1.1	-3.0
AK3	adenylate kinase 3	224655_at	-2.506	0.001	1842	1039	-1.8	1.1	-2.8

TMEM45A	transmembrane protein 45A	219410_at	-2.502	0.001	7233	3294	-2.2	-1.1	5.5
CD44	CD44 molecule (Indian blood group)	217523_at	-2.498	0.001	999	364	-2.7	1.5	1.2
FCHO2	FCH domain only 2	228220_at	-2.494	0.001	660	313	-2.1	1.4	-1.5
KMT5B	lysine methyltransferase 5B	218242_s_at	-2.488	0.001	763	458	-1.7	1.0	-2.1
LCE1B	late cornified envelope 1B	1560531_at	-2.477	0.001	3484	1080	-3.2	-1.5	ND
OTUD1	OTU deubiquitinase 1	226140_s_at	-2.476	0.001	558	354	-1.6	-1.7	-4.3
GNAI1	G protein subunit alpha i1	227692_at	-2.476	0.001	745	234	-3.2	-1.4	1.0
LOR	loricrin	207720_at	-2.470	0.001	9162	3873	-2.4	-1.7	1.0
ALDH2	aldehyde dehydrogenase 2 family member	201425_at	-2.464	0.001	2165	890	-2.4	-1.4	-1.2
YBX3	Y-box binding protein 3	201161_s_at	-2.463	0.001	2666	1525	-1.7	-1.5	2.1
FLG2	filaggrin family member 2	1569410_at	-2.463	0.001	5599	1736	-3.2	-1.6	ND
FLG	filaggrin	215704_at	-2.461	0.001	5199	2438	-2.1	-1.2	1.0
TXNIP	thioredoxin interacting protein	201010_s_at	-2.461	0.001	4016	2639	-1.5	-1.6	-4.8
SEMA3C	semaphorin 3C	203789_s_at	-2.460	0.001	1491	713	-2.1	-1.3	-3.2
GJA1	gap junction protein alpha 1	201667_at	-2.456	0.001	6420	3075	-2.1	-1.1	-1.0
CMTM6	CKLF like MARVEL transmembrane domain containing 6	217947_at	-2.454	0.001	3109	2063	-1.5	1.2	2.3
DSP	desmoplakin	200606_at	-2.450	0.001	7193	4008	-1.8	-1.5	1.1
GCLC	glutamate-cysteine ligase catalytic subunit	202923_s_at	-2.449	0.001	634	340	-1.9	1.1	-1.6
KRAS	KRAS proto-oncogene, GTPase	204009_s_at	-2.446	0.001	841	544	-1.5	1.2	-1.4
RORA	RAR related orphan receptor A	226682_at	-2.443	0.001	1174	281	-4.2	-1.7	-1.1
ABI1	abl interactor 1	209028_s_at	-2.438	0.001	692	400	-1.7	-1.2	-2.2
TRIM29	tripartite motif containing 29	202504_at	-2.424	0.001	4915	1817	-2.7	-1.8	1.1
FOSL2	FOS like 2, AP-1 transcription factor subunit	228188_at	-2.423	0.001	582	297	-2.0	-1.2	-1.2
HECA	hdc homolog, cell cycle regulator	218603_at	-2.419	0.001	919	530	-1.7	-1.2	-2.3
CHIC2	cysteine rich hydrophobic domain 2	219492_at	-2.418	0.001	692	378	-1.8	1.1	-1.3
KLF10	Kruppel like factor 10	202393_s_at	-2.414	0.001	1304	806	-1.6	-1.3	2.3
PGRMC2	progesterone receptor membrane component 2	213227_at	-2.412	0.001	828	408	-2.0	-1.7	-3.2
DIPK2A	divergent protein kinase domain 2A	226464_at	-2.409	0.001	693	307	-2.3	-1.1	1.2

FAM8A1	family with sequence similarity 8 member A1	203420_at	-2.404	0.001	798	466	-1.7	-1.0	-1.5
OSBPL8	oxysterol binding protein like 8	212585_at	-2.400	0.001	992	469	-2.1	1.3	-1.2
EIF1AX	eukaryotic translation initiation factor 1A X-linked	201018_at	-2.397	0.001	583	304	-1.9	-1.2	-1.2
ABLIM1	actin binding LIM protein 1	200965_s_at	-2.390	0.001	2014	742	-2.7	-1.6	1.3
FBXO9	F-box protein 9	212987_at	-2.376	0.001	1285	827	-1.6	1.0	-3.0
AHNAK2	AHNAK nucleoprotein 2	212992_at	-2.376	0.001	1369	493	-2.8	-2.9	-1.5
VPS4B	vacuolar protein sorting 4 homolog B	218171_at	-2.372	0.001	879	512	-1.7	-1.0	-1.1
RNF11	ring finger protein 11	208924_at	-2.372	0.001	1248	730	-1.7	1.1	-1.2
BORCS7	BLOC-1 related complex subunit 7	225334_at	-2.370	0.001	760	407	-1.9	1.1	-7.3
SLC25A46	solute carrier family 25 member 46	226831_at	-2.368	0.001	470	216	-2.2	1.4	-2.3
OPTN	optineurin	202073_at	-2.361	0.001	508	309	-1.6	-1.2	-11.5
UBE2K	ubiquitin conjugating enzyme E2 K	225179_at	-2.359	0.001	839	466	-1.8	-1.1	1.4
ALDH3A2	aldehyde dehydrogenase 3 family member A2	202054_s_at	-2.357	0.001	1043	454	-2.3	-2.3	-1.1
IMPA2	inositol monophosphatase 2	203126_at	-2.357	0.001	1390	492	-2.8	-1.3	-2.4
SETD7	SET domain containing 7, histone lysine methyltransferase	224928_at	-2.349	0.001	836	517	-1.6	-1.1	-1.1
KLF3	Kruppel like factor 3	225133_at	-2.347	0.001	1100	535	-2.1	-1.3	-1.8
CXCL14	C-X-C motif chemokine ligand 14	218002_s_at	-2.338	0.001	3655	1715	-2.1	-1.9	3.0
RNF144B	ring finger protein 144B	228153_at	-2.334	0.001	862	386	-2.2	1.1	-2.3
SPINK5	serine peptidase inhibitor, Kazal type 5	205185_at	-2.328	0.001	4916	1419	-3.5	1.0	-1.3
KLK5	kallikrein related peptidase 5	222242_s_at	-2.327	0.001	1826	607	-3.0	-1.3	-1.0
ZBTB33	zinc finger and BTB domain containing 33	226255_at	-2.325	0.001	801	519	-1.5	-1.1	-1.8
TXNDC9	thioredoxin domain containing 9	211758_x_at	-2.319	0.001	823	523	-1.6	1.6	1.5
CAMK1D	calcium/calmodulin dependent protein kinase ID	235626_at	-2.311	0.001	580	248	-2.3	-1.3	-1.2
PPL	periplakin	203407_at	-2.309	0.001	1443	550	-2.6	-1.5	-1.2
DENND4C	DENN domain containing 4C	226867_at	-2.304	0.001	779	417	-1.9	-1.3	-1.7
RAD23B	RAD23 homolog B, nucleotide excision repair protein	223598_at	-2.304	0.001	657	383	-1.7	-1.2	-1.0
ERBB2	erb-b2 receptor tyrosine kinase 2	216836_s_at	-2.302	0.001	815	444	-1.8	-1.5	1.6

LCE2B	late cornified envelope 2B	207710_at	-2.300	0.001	1970	606	-3.2	-1.9	-1.0
RBPJ	recombination signal binding protein for immunoglobulin kappa J region	207785_s_at	-2.294	0.001	794	517	-1.5	-1.1	-1.4
UBE2E2	ubiquitin conjugating enzyme E2 E2	225651_at	-2.294	0.001	613	353	-1.7	-1.0	2.3
JMY	junction mediating and regulatory protein, p53 cofactor	226352_at	-2.294	0.001	599	238	-2.5	1.2	-1.7
NFIB	nuclear factor I B	213029_at	-2.282	0.001	704	319	-2.2	-1.9	-1.4
CLCA2	chloride channel accessory 2	206165_s_at	-2.281	0.001	2739	1044	-2.6	1.1	-1.0
TMEM123	transmembrane protein 123	211967_at	-2.270	0.001	1922	1034	-1.9	1.2	1.6
KIF5B	kinesin family member 5B	224662_at	-2.268	0.001	1497	839	-1.8	1.5	1.8
DST	dystonin	204455_at	-2.266	0.001	1217	311	-3.9	-1.6	1.1
NIPSNAP3A	nipsnap homolog 3A	224436_s_at	-2.264	0.001	519	238	-2.2	1.2	-2.8
OGFRL1	opioid growth factor receptor like 1	226810_at	-2.260	0.001	765	339	-2.3	1.2	1.1
GATA3	GATA binding protein 3	209604_s_at	-2.256	0.001	1610	395	-4.1	-3.2	1.1
PANK3	pantothenate kinase 3	221751_at	-2.253	0.001	939	470	-2.0	1.1	1.2
NDFIP2	Nedd4 family interacting protein 2	224802_at	-2.253	0.001	851	367	-2.3	-1.5	3.4
SBSN	suprabasin	235272_at	-2.252	0.001	4194	1647	-2.5	-1.5	1.1
ARL5A	ADP ribosylation factor like GTPase 5A	218150_at	-2.252	0.001	1824	946	-1.9	1.4	-1.3
UBQLN1	ubiquilin 1	222990_at	-2.249	0.001	1620	1079	-1.5	1.1	-1.3
STK38L	serine/threonine kinase 38 like	212572_at	-2.249	0.001	509	276	-1.8	1.5	-1.4
MOB4	MOB family member 4, phocein	202919_at	-2.247	0.001	682	434	-1.6	1.3	-1.1
VPS36	vacuolar protein sorting 36 homolog	222478_at	-2.247	0.001	700	369	-1.9	-1.2	-1.8
KLF4	Kruppel like factor 4	221841_s_at	-2.246	0.001	3075	1547	-2.0	-1.4	1.4
GJB3	gap junction protein beta 3	205490_x_at	-2.244	0.001	728	302	-2.4	-1.5	-1.1
SH3YL1	SH3 and SYLF domain containing 1	204019_s_at	-2.244	0.001	879	417	-2.1	-1.4	-2.4
CPEB4	cytoplasmic polyadenylation element binding protein 4	224828_at	-2.242	0.001	489	157	-3.1	1.4	-1.4
IRX2	iroquois homeobox 2	228462_at	-2.242	0.001	499	210	-2.4	-1.2	-2.8
GSKIP	GSK3B interacting protein	223239_at	-2.239	0.001	711	417	-1.7	1.7	-1.2
SCAF11	SR-related CTD associated factor 11	206989_s_at	-2.236	0.001	942	600	-1.6	1.3	-1.8
BNIP3L	BCL2 interacting protein 3 like	221478_at	-2.230	0.001	1568	1001	-1.6	1.2	-2.9
NOTCH2NLA	notch 2 N-terminal like A	214722_at	-2.229	0.001	1967	1230	-1.6	1.5	-1.2
IDE	insulin degrading enzyme	203327_at	-2.225	0.001	1304	472	-2.8	-1.1	1.1

DEGS1	delta 4-desaturase, sphingolipid 1	207431_s_at	-2.224	0.001	2263	1086	-2.1	-2.0	1.7
NET1	neuroepithelial cell transforming 1	201830_s_at	-2.220	0.001	906	381	-2.4	-1.4	3.0
LY6D	lymphocyte antigen 6 family member D	206276_at	-2.219	0.001	1575	544	-2.9	-1.6	-1.1
ARRDC4	arrestin domain containing 4	225283_at	-2.215	0.001	697	381	-1.8	-1.2	-7.7
CA12	carbonic anhydrase 12	203963_at	-2.212	0.001	1465	593	-2.5	-1.6	1.9
BCL11A	BAF chromatin remodeling complex subunit BCL11A	222891_s_at	-2.210	0.001	635	152	-4.2	-1.5	-1.3
KLK11	kallikrein related peptidase 11	205470_s_at	-2.204	0.001	874	348	-2.5	-1.6	-1.1
HSPB8	heat shock protein family B (small) member 8	221667_s_at	-2.194	0.001	986	466	-2.1	-1.0	-3.0
KLHL9	kelch like family member 9	213233_s_at	-2.190	0.001	730	458	-1.6	-1.3	-1.9
PLEKHA1	pleckstrin homology domain containing A1	226247_at	-2.189	0.001	763	346	-2.2	-1.3	-1.3
FGFR3	fibroblast growth factor receptor 3	204379_s_at	-2.189	0.001	1189	311	-3.8	-2.7	-1.0
BPIFC	BPI fold containing family C	1555773_at	-2.185	0.001	889	305	-2.9	-1.1	ND
INAVA	innate immunity activator	219010_at	-2.184	0.001	833	302	-2.8	-1.5	1.8
F11R	F11 receptor	223000_s_at	-2.182	0.001	1335	770	-1.7	-1.1	-1.2
KRT80	keratin 80	231849_at	-2.181	0.001	968	294	-3.3	-1.3	1.0
PLAGL1	PLAG1 like zinc finger 1	209318_x_at	-2.179	0.001	826	405	-2.0	-1.3	1.4
IL6ST	interleukin 6 signal transducer	212195_at	-2.176	0.001	1766	1113	-1.6	1.3	2.7
EEA1	early endosome antigen 1	225885_at	-2.176	0.001	557	257	-2.2	1.3	1.2
PLXDC2	plexin domain containing 2	226865_at	-2.175	0.001	642	332	-1.9	-1.1	-1.4
EMP2	epithelial membrane protein 2	225078_at	-2.173	0.001	1280	545	-2.3	-1.7	3.6
BCL6	BCL6 transcription repressor	203140_at	-2.172	0.001	1621	921	-1.8	-1.1	1.6
RMND5A	required for meiotic nuclear division 5 homolog A	212482_at	-2.172	0.001	477	289	-1.6	-1.2	-2.0
PDLIM1	PDZ and LIM domain 1	208690_s_at	-2.172	0.001	2031	1285	-1.6	-1.2	5.9
ESRP1	epithelial splicing regulatory protein 1	225846_at	-2.171	0.001	1254	428	-2.9	-1.1	-6.1
ITSN2	intersectin 2	209898_x_at	-2.171	0.001	875	490	-1.8	1.5	-1.1
MED13	mediator complex subunit 13	201987_at	-2.168	0.001	1055	631	-1.7	1.0	-1.3
GLTP	glycolipid transfer protein	226177_at	-2.167	0.001	4904	2298	-2.1	-1.3	1.5
SPINT2	serine peptidase inhibitor, Kunitz type 2	210715_s_at	-2.165	0.001	935	427	-2.2	-1.5	-1.3
MRTFB	myocardin related transcription factor B	218259_at	-2.165	0.001	693	440	-1.6	1.0	-1.1

SAR1B	secretion associated Ras related GTPase 1B	226742_at	-2.164	0.001	510	294	-1.7	1.0	1.2
KRT23	keratin 23	218963_s_at	-2.158	0.001	1164	296	-3.9	-1.2	1.1
CAV2	caveolin 2	203323_at	-2.157	0.001	1164	668	-1.7	-1.5	-1.8
ZYG11B	zyg-11 family member B, cell cycle regulator	225338_at	-2.157	0.001	447	271	-1.7	1.1	1.1
ETFRF1	electron transfer flavoprotein regulatory factor 1	225469_at	-2.155	0.001	964	575	-1.7	1.1	-1.1
PPP1CB	protein phosphatase 1 catalytic subunit beta	201409_s_at	-2.153	0.001	1578	766	-2.1	1.5	-1.1
MYOF	myoferlin	201798_s_at	-2.153	0.001	1383	876	-1.6	-1.1	17.4
GPR157	G protein-coupled receptor 157	227970_at	-2.153	0.001	574	288	-2.0	-1.4	-1.3
ITPRID2	ITPR interacting domain containing 2	202506_at	-2.152	0.001	1500	943	-1.6	1.0	1.8
CCSER2	coiled-coil serine rich protein 2	209379_s_at	-2.152	0.001	514	284	-1.8	-1.2	-1.2
EGLN3	egl-9 family hypoxia inducible factor 3	219232_s_at	-2.152	0.001	352	138	-2.6	-1.7	-1.5
RAB11FIP1	RAB11 family interacting protein 1	219681_s_at	-2.151	0.001	693	345	-2.0	-1.3	-1.1
SERPINA12	serpin family A member 12	1552544_at	-2.151	0.001	791	283	-2.8	-1.6	ND
RAB27B	RAB27B, member RAS oncogene family	228708_at	-2.149	0.001	1079	279	-3.9	-1.1	1.6
C2orf69	chromosome 2 open reading frame 69	238974_at	-2.148	0.001	832	478	-1.7	1.2	-1.0
LYPLA1	lysophospholipase 1	212449_s_at	-2.145	0.001	1956	952	-2.1	1.4	1.9
SMAD1	SMAD family member 1	210993_s_at	-2.144	0.001	499	270	-1.8	-1.0	-3.2
ZMPSTE24	zinc metallopeptidase STE24	202939_at	-2.143	0.001	1180	780	-1.5	1.2	1.5
CSNK1A1	casein kinase 1 alpha 1	208865_at	-2.143	0.001	2372	1353	-1.8	-1.1	-1.7
SDC1	syndecan 1	201287_s_at	-2.142	0.001	2492	1090	-2.3	-1.5	1.9
EXPH5	exophilin 5	214734_at	-2.142	0.001	831	279	-3.0	-2.8	1.4
SYTL1	synaptotagmin like 1	227134_at	-2.141	0.001	793	223	-3.6	-1.4	1.1
CREG1	cellular repressor of E1A stimulated genes 1	201200_at	-2.140	0.001	3706	2024	-1.8	1.2	-2.0
PTPRF	protein tyrosine phosphatase receptor type F	200636_s_at	-2.139	0.001	1486	691	-2.1	-1.9	13.1
CEBPG	CCAAT enhancer binding protein gamma	225527_at	-2.138	0.001	350	200	-1.8	1.2	1.1
SPOPL	speckle type BTB/POZ protein like	225658_at	-2.136	0.001	424	210	-2.0	1.2	1.1
MBNL1	muscleblind like splicing regulator 1	201153_s_at	-2.136	0.001	1716	1088	-1.6	1.1	-1.1

SMAD5	SMAD family member 5	225223_at	-2.132	0.001	490	258	-1.9	-1.1	-1.0
IFNGR1	interferon gamma receptor 1	211676_s_at	-2.132	0.001	910	589	-1.5	1.6	-1.3
SREK1	splicing regulatory glutamic acid and lysine rich protein 1	212721_at	-2.126	0.001	762	476	-1.6	1.4	-1.5
ASS1	argininosuccinate synthase 1	207076_s_at	-2.125	0.001	502	254	-2.0	-1.9	1.4
CSTA	cystatin A	204971_at	-2.120	0.001	8691	4742	-1.8	1.2	1.2
PIK3R1	phosphoinositide-3-kinase regulatory subunit 1	212240_s_at	-2.119	0.001	1197	649	-1.8	1.3	-1.0
TMTC3	transmembrane and tetratricopeptide repeat containing 3	226604_at	-2.117	0.001	414	136	-3.0	1.0	1.7
KLK7	kallikrein related peptidase 7	205778_at	-2.114	0.001	1925	769	-2.5	1.1	-1.0
PKP1	plakophilin 1	221854_at	-2.114	0.001	2006	828	-2.4	-2.1	1.0
FRMD4B	FERM domain containing 4B	213056_at	-2.111	0.001	522	302	-1.7	-1.5	-1.5
COX7B	cytochrome c oxidase subunit 7B	202110_at	-2.110	0.001	3528	2309	-1.5	1.4	1.2
NXT2	nuclear transport factor 2 like export factor 2	209628_at	-2.109	0.001	317	152	-2.1	1.2	1.0
SMARCA2	SWI/SNF related, matrix associated, actin dependent regulator of chromatin, subfamily a, member 2	206542_s_at	-2.107	0.001	535	329	-1.6	-1.5	-2.4
LY6G6C	lymphocyte antigen 6 family member G6C	207114_at	-2.105	0.001	1824	580	-3.1	-1.5	1.1
ANKRD22	ankyrin repeat domain 22	238439_at	-2.100	0.001	556	167	-3.3	1.1	-1.3
JUP	junction plakoglobin	201015_s_at	-2.098	0.001	2595	1188	-2.2	-1.6	-1.3
HSDL2	hydroxysteroid dehydrogenase like 2	209513_s_at	-2.096	0.001	591	288	-2.1	-1.2	-1.2
YES1	YES proto-oncogene 1, Src family tyrosine kinase	202932_at	-2.095	0.001	507	288	-1.8	1.5	2.0
WDR26	WD repeat domain 26	224897_at	-2.094	0.001	822	471	-1.7	1.4	-1.2
EPPK1	epiplakin 1	232164_s_at	-2.088	0.001	1517	503	-3.0	-2.1	-1.0
FBXO3	F-box protein 3	218432_at	-2.085	0.001	467	205	-2.3	1.1	-1.7
CNOT6	CCR4-NOT transcription complex subunit 6	222476_at	-2.083	0.001	400	244	-1.6	-1.1	-1.3
GPBP1L1	GC-rich promoter binding protein 1 like 1	217877_s_at	-2.083	0.001	597	395	-1.5	-1.2	1.1
CRCT1	cysteine rich C-terminal 1	220620_at	-2.081	0.001	803	293	-2.7	-1.2	1.1
FGFR1OP2	FGFR1 oncogene partner 2	233898_s_at	-2.080	0.001	397	216	-1.8	1.2	-1.4

CDC42SE2	CDC42 small effector 2	224709_s_at	-2.079	0.001	801	484	-1.7	1.0	-2.4
TSPAN5	tetraspanin 5	225387_at	-2.078	0.001	348	178	-2.0	-1.2	1.2
IL20RB	interleukin 20 receptor subunit beta	228575_at	-2.078	0.001	503	148	-3.4	-1.8	1.1
DSG1	desmoglein 1	206642_at	-2.077	0.001	2871	963	-3.0	-1.4	1.2
NEBL	nebulette	203962_s_at	-2.077	0.001	571	153	-3.7	-1.5	-1.1
C12orf29	chromosome 12 open reading frame 29	213701_at	-2.076	0.001	440	168	-2.6	-1.1	1.6
EFR3A	EFR3 homolog A	212149_at	-2.074	0.001	1465	964	-1.5	1.1	1.7
ZBTB44	zinc finger and BTB domain containing 44	225845_at	-2.073	0.001	530	212	-2.5	-1.0	-1.1
SCEL	sciellin	206884_s_at	-2.073	0.001	1054	221	-4.8	-1.3	-1.0
CD47	CD47 molecule	226016_at	-2.073	0.001	962	609	-1.6	-1.2	-2.2
GRHL1	grainyhead like transcription factor 1	222830_at	-2.072	0.001	1529	441	-3.5	1.2	1.2
ATP7A	ATPase copper transporting alpha	205197_s_at	-2.071	0.001	553	267	-2.1	1.1	-2.7
SCAMP1	secretory carrier membrane protein 1	212416_at	-2.071	0.001	753	476	-1.6	-1.1	1.3
GOLPH3L	golgi phosphoprotein 3 like	218361_at	-2.071	0.001	413	261	-1.6	-1.0	-1.1
GGCT	gamma-glutamylcyclotransferase	215380_s_at	-2.070	0.001	2675	1479	-1.8	2.0	2.0
TCF4	transcription factor 4	212386_at	-2.068	0.001	1437	848	-1.7	-1.1	3.2
S100A14	S100 calcium binding protein A14	218677_at	-2.067	0.001	1432	707	-2.0	-1.4	1.1
TUT7	terminal uridylyl transferase 7	220933_s_at	-2.064	0.001	729	382	-1.9	1.3	-1.0
CST6	cystatin E/M	206595_at	-2.060	0.001	6510	1369	-4.8	-1.4	-1.3
STRN	striatin	227422_at	-2.059	0.001	300	151	-2.0	-1.0	1.0
LGALSL	galectin like	226188_at	-2.059	0.001	1827	895	-2.0	-1.4	1.2
SEPTIN10	septin 10	212698_s_at	-2.055	0.001	1207	792	-1.5	-1.1	-1.3
FAM126B	family with sequence similarity 126 member B	229460_at	-2.054	0.001	330	134	-2.5	1.9	1.1
HEBP2	heme binding protein 2	203430_at	-2.051	0.001	1576	905	-1.7	-1.2	-1.2
MAST4	microtubule associated serine/threonine kinase family member 4	225611_at	-2.049	0.001	612	259	-2.4	-1.1	-1.5
OXR1	oxidation resistance 1	222553_x_at	-2.049	0.001	519	236	-2.2	1.2	-1.3
PER3	period circadian regulator 3	221045_s_at	-2.048	0.001	380	222	-1.7	1.3	-1.6
CLTB	clathrin light chain B	211043_s_at	-2.045	0.001	1578	834	-1.9	-1.1	-1.2
SMIM14	small integral membrane protein 14	227052_at	-2.045	0.001	707	347	-2.0	-1.1	-3.0
MAF	MAF bZIP transcription factor	209348_s_at	-2.044	0.001	1584	854	-1.9	-1.2	-6.9
KDM7A	lysine demethylase 7A	221778_at	-2.041	0.001	399	240	-1.7	1.2	-1.0

ZNF770	zinc finger protein 770	225517_at	-2.039	0.001	776	352	-2.2	1.2	-1.1
WDR11	WD repeat domain 11	229694_at	-2.039	0.001	483	206	-2.3	2.3	-1.6
MPZL3	myelin protein zero like 3	227747_at	-2.038	0.001	522	144	-3.6	1.0	1.2
PSMD12	proteasome 26S subunit, non-ATPase 12	202353_s_at	-2.036	0.001	590	309	-1.9	1.0	1.7
CAT	catalase	201432_at	-2.035	0.001	2116	1363	-1.6	-1.2	-3.6
RAB25	RAB25, member RAS oncogene family	218186_at	-2.033	0.001	1396	499	-2.8	-1.1	1.0
AQP3	aquaporin 3 (Gill blood group)	39248_at	-2.031	0.001	4125	1810	-2.3	-1.1	1.3
BMPR2	bone morphogenetic protein receptor type 2	225144_at	-2.030	0.001	1044	589	-1.8	1.3	-1.7
CCDC3	coiled-coil domain containing 3	223316_at	-2.028	0.001	1062	489	-2.2	-3.1	1.4
NIPAL2	NIPA like domain containing 2	227001_at	-2.025	0.001	796	297	-2.7	-1.1	-1.0
CDS1	CDP-diacylglycerol synthase 1	205709_s_at	-2.025	0.001	389	104	-3.8	1.1	-1.1
REL	REL proto-oncogene, NF-kB subunit	235242_at	-2.024	0.001	758	400	-1.9	1.2	-1.0
CRYBG1	crystallin beta-gamma domain containing 1	212543_at	-2.023	0.001	1071	477	-2.2	-1.2	1.8
PTPN21	protein tyrosine phosphatase non-receptor type 21	226380_at	-2.021	0.001	345	183	-1.9	-1.6	-2.4
FAM135A	family with sequence similarity 135 member A	223497_at	-2.020	0.001	410	145	-2.8	1.0	-1.2
F2RL1	F2R like trypsin receptor 1	213506_at	-2.020	0.001	494	177	-2.8	-1.6	1.1
LYPD3	LY6/PLAUR domain containing 3	204952_at	-2.019	0.001	3004	1461	-2.1	-1.3	-1.2
PPP3CA	protein phosphatase 3 catalytic subunit alpha	202457_s_at	-2.018	0.001	939	581	-1.6	-1.2	-1.1
ITGB4	integrin subunit beta 4	204990_s_at	-2.017	0.001	494	261	-1.9	-1.8	-1.2
SCNN1A	sodium channel epithelial 1 alpha subunit	203453_at	-2.016	0.001	895	363	-2.5	-1.3	-1.0
PRRG4	proline rich and Gla domain 4	238513_at	-2.015	0.001	682	210	-3.3	-1.2	1.2
MKNK2	MAPK interacting serine/threonine kinase 2	218205_s_at	-2.015	0.001	1977	1303	-1.5	-1.1	-1.9
LIN7C	lin-7 homolog C, crumbs cell polarity complex component	223350_x_at	-2.011	0.001	584	341	-1.7	-1.1	1.3
PNPLA8	patatin like phospholipase domain containing 8	223310_x_at	-2.009	0.001	920	572	-1.6	1.9	-1.2
XG	Xg glycoprotein (Xg blood group)	1554062_at	-2.008	0.001	383	108	-3.6	-1.2	ND

CPEB2	cytoplasmic polyadenylation element binding protein 2	226939_at	-2.005	0.001	1397	582	-2.4	-1.1	-11.9
TAOK1	TAO kinase 1	224769_at	-2.005	0.001	1201	755	-1.6	1.1	1.1
MAL2	mal, T cell differentiation protein 2 (gene/pseudogene)	224650_at	-2.003	0.001	1208	395	-3.1	-1.1	-1.1
SLAIN2	SLAIN motif family member 2	224844_at	-1.999	0.001	482	260	-1.9	-1.1	-1.0
OCLN	occludin	227492_at	-1.999	0.001	333	153	-2.2	-1.2	2.2
IRF6	interferon regulatory factor 6	202597_at	-1.998	0.001	965	444	-2.2	-2.0	-1.1
ANKRD12	ankyrin repeat domain 12	212286_at	-1.996	0.001	305	182	-1.7	1.3	-2.0
FAM177A1	family with sequence similarity 177 member A1	227029_at	-1.995	0.001	654	371	-1.8	-1.0	-1.8
RAPGEFL1	Rap guanine nucleotide exchange factor like 1	218657_at	-1.993	0.001	462	182	-2.5	-1.7	1.0
VAV3	vav guanine nucleotide exchange factor 3	218807_at	-1.992	0.001	491	215	-2.3	-1.5	1.3
CLK4	CDC like kinase 4	210346_s_at	-1.992	0.001	467	237	-2.0	1.6	-1.7
VPS13A	vacuolar protein sorting 13 homolog A	227988_s_at	-1.992	0.001	405	211	-1.9	1.4	1.0
MAPK6	mitogen-activated protein kinase 6	207121_s_at	-1.992	0.001	1354	741	-1.8	1.3	1.2
PARD6G	par-6 family cell polarity regulator gamma	227204_at	-1.991	0.001	647	388	-1.7	-1.7	-2.2
AZGP1	alpha-2-glycoprotein 1, zinc-binding	209309_at	-1.990	0.001	1273	448	-2.8	-1.2	2.0
CCDC186	coiled-coil domain containing 186	227701_at	-1.990	0.001	252	90	-2.8	1.2	-1.1
YTHDC2	YTH domain containing 2	213077_at	-1.988	0.001	364	195	-1.9	1.3	-1.6
TACC2	transforming acidic coiled-coil containing protein 2	202289_s_at	-1.988	0.001	618	231	-2.7	-1.6	1.8
FAM174A	family with sequence similarity 174 member A	226752_at	-1.986	0.001	574	345	-1.7	-1.3	-1.5
CDH1	cadherin 1	201131_s_at	-1.985	0.001	2607	1594	-1.6	-1.0	-94.8
TANK	TRAF family member associated NFKB activator	209451_at	-1.984	0.001	556	367	-1.5	1.8	-1.2
GNA15	G protein subunit alpha 15	205349_at	-1.984	0.001	426	258	-1.7	1.3	-1.2
BLMH	bleomycin hydrolase	202179_at	-1.983	0.001	912	411	-2.2	-1.5	-1.5
SUB1	SUB1 regulator of transcription	221727_at	-1.981	0.001	461	251	-1.8	1.7	-1.0
PROM2	prominin 2	1552797_s_at	-1.978	0.001	737	269	-2.7	-1.3	ND
KLK8	kallikrein related peptidase 8	206125_s_at	-1.977	0.001	1254	594	-2.1	1.1	-1.2

WAPL	WAPL cohesin release factor	212267_at	-1.972	0.001	491	300	-1.6	1.0	-1.3
PPP2R3A	protein phosphatase 2 regulatory subunit B''alpha	209633_at	-1.972	0.001	600	355	-1.7	-1.8	-1.3
CDSN	corneodesmosin	206192_at	-1.969	0.001	1705	752	-2.3	-1.3	1.1
ATL3	atlastin GTPase 3	224893_at	-1.969	0.001	1222	795	-1.5	-1.3	-1.1
COL4A5	collagen type IV alpha 5 chain	213110_s_at	-1.968	0.001	393	113	-3.5	-1.5	1.1
DNAJC21	DnaJ heat shock protein family (Hsp40) member C21	235032_at	-1.964	0.001	429	241	-1.8	1.3	1.0
ETS2	ETS proto-oncogene 2, transcription factor	201328_at	-1.964	0.001	577	304	-1.9	-1.3	1.4
TET3	tet methylcytosine dioxygenase 3	235542_at	-1.963	0.001	393	221	-1.8	-1.3	1.8
QSER1	glutamine and serine rich 1	226265_at	-1.962	0.001	758	442	-1.7	-1.2	2.6
ELOVL7	ELOVL fatty acid elongase 7	227180_at	-1.961	0.001	919	246	-3.7	1.2	1.1
RB1CC1	RB1 inducible coiled-coil 1	202033_s_at	-1.961	0.001	841	425	-2.0	1.7	-1.1
PAWR	pro-apoptotic WT1 regulator	226223_at	-1.960	0.001	665	191	-3.5	1.0	3.2
ZNF782	zinc finger protein 782	228156_at	-1.960	0.001	286	140	-2.0	-1.1	-1.5
CYBRD1	cytochrome b reductase 1	222453_at	-1.959	0.001	1447	876	-1.7	-1.5	1.1
PCMTD1	protein-L-isoaspartate (D-aspartate) O-methyltransferase domain containing 1	226119_at	-1.959	0.001	763	398	-1.9	1.4	-3.2
RNF217	ring finger protein 217	226885_at	-1.959	0.001	245	81	-3.0	1.1	1.2
USP38	ubiquitin specific peptidase 38	223288_at	-1.959	0.001	470	244	-1.9	1.3	-1.3
C5orf24	chromosome 5 open reading frame 24	224876_at	-1.958	0.001	1069	613	-1.7	-1.0	-1.5
RBM27	RNA binding motif protein 27	225326_at	-1.956	0.001	507	301	-1.7	1.1	1.3
MCUR1	mitochondrial calcium uniporter regulator 1	227451_s_at	-1.956	0.001	460	268	-1.7	-1.1	1.1
GPX3	glutathione peroxidase 3	201348_at	-1.956	0.001	2226	1233	-1.8	-2.1	-1.1
CLIP1	CAP-Gly domain containing linker protein 1	210716_s_at	-1.954	0.001	552	292	-1.9	-1.0	-1.9
ACTR3	actin related protein 3	228603_at	-1.953	0.001	381	203	-1.9	1.4	-1.0
MYCBP2	MYC binding protein 2	201960_s_at	-1.952	0.001	1808	989	-1.8	-1.0	-1.8
LRATD1	LRAT domain containing 1	225667_s_at	-1.951	0.001	1142	447	-2.6	1.0	1.2
KCNK1	potassium two pore domain channel subfamily K member 1	204679_at	-1.950	0.001	440	185	-2.4	-1.2	-1.0

RBBP8	RB binding protein 8, endonuclease	203344_s_at	-1.950	0.001	636	414	-1.5	1.7	1.7
CDHR1	cadherin related family member 1	213369_at	-1.950	0.001	312	117	-2.7	-2.7	1.1
TJP2	tight junction protein 2	202085_at	-1.950	0.001	470	237	-2.0	1.2	-1.2
CLEC2B	C-type lectin domain family 2 member B	209732_at	-1.950	0.001	1379	831	-1.7	1.4	2.5
BAZ1A	bromodomain adjacent to zinc finger domain 1A	217986_s_at	-1.949	0.001	504	295	-1.7	1.8	1.7
CSNK2A2	casein kinase 2 alpha 2	203575_at	-1.948	0.001	670	365	-1.8	-1.3	-3.7
CUL5	cullin 5	203531_at	-1.947	0.001	621	342	-1.8	1.2	1.1
PTPRK	protein tyrosine phosphatase receptor type K	203038_at	-1.947	0.001	640	366	-1.8	1.0	1.5
USO1	USO1 vesicle transport factor	201832_s_at	-1.945	0.001	934	595	-1.6	1.2	1.1
HAL	histidine ammonia-lyase	217521_at	-1.944	0.001	540	208	-2.6	-1.2	1.1
CASP14	caspase 14	231722_at	-1.943	0.001	594	206	-2.9	-1.3	-1.0
GATM	glycine amidinotransferase	216733_s_at	-1.942	0.001	664	295	-2.2	-2.1	-1.9
SULT2B1	sulfotransferase family 2B member 1	205759_s_at	-1.942	0.001	482	183	-2.6	-1.2	1.0
OGA	O-GlcNAcase	223494_at	-1.941	0.001	931	563	-1.7	1.7	-1.2
FGFR2	fibroblast growth factor receptor 2	203638_s_at	-1.939	0.001	422	159	-2.7	-2.7	-1.0
LNPEP	leucyl and cystinyl aminopeptidase	225176_at	-1.939	0.001	764	408	-1.9	-1.0	-1.2
COL17A1	collagen type XVII alpha 1 chain	204636_at	-1.938	0.001	413	163	-2.5	-3.1	-1.0
SDR16C5	short chain dehydrogenase/reductase family 16C member 5	238017_at	-1.937	0.001	1111	349	-3.2	1.0	1.0
PPM1B	protein phosphatase, Mg2+/Mn2+ dependent 1B	209296_at	-1.936	0.001	616	352	-1.8	1.3	-2.0
DMXL1	Dmx like 1	203791_at	-1.935	0.001	441	197	-2.2	1.0	-11.2
PDZD2	PDZ domain containing 2	209493_at	-1.934	0.001	514	161	-3.2	-1.6	-1.3
RAB3D	RAB3D, member RAS oncogene family	225001_at	-1.934	0.001	749	450	-1.7	-1.6	-1.5
EHF	ETS homologous factor	225645_at	-1.934	0.001	1276	409	-3.1	1.2	3.5
TMEM40	transmembrane protein 40	222892_s_at	-1.934	0.001	770	331	-2.3	1.2	-1.1
POF1B	POF1B actin binding protein	219756_s_at	-1.932	0.001	474	134	-3.5	-1.5	1.0
RPS6KB1	ribosomal protein S6 kinase B1	226660_at	-1.932	0.001	485	277	-1.8	1.3	1.1
CLINT1	clathrin interactor 1	201769_at	-1.931	0.001	1118	667	-1.7	1.2	-1.0
CLK1	CDC like kinase 1	214683_s_at	-1.929	0.001	663	363	-1.8	1.9	-1.2
SH3RF2	SH3 domain containing ring finger 2	243582_at	-1.928	0.001	363	123	-2.9	-1.5	1.3

SGMS1	sphingomyelin synthase 1	212989_at	-1.925	0.001	331	158	-2.1	-1.1	1.4
BCL11B	BAF chromatin remodeling complex subunit BCL11B	222895_s_at	-1.925	0.001	914	378	-2.4	-1.3	-1.0
CRIPAK	cysteine rich PAK1 inhibitor	228318_s_at	-1.923	0.001	520	335	-1.6	1.2	-1.4
GAN	gigaxonin	228567_at	-1.922	0.001	826	194	-4.3	-1.8	1.1
CAPNS2	calpain small subunit 2	223832_s_at	-1.921	0.001	656	221	-3.0	-1.3	1.3
SNX13	sorting nexin 13	227031_at	-1.920	0.001	336	161	-2.1	1.1	-1.3
CREB1	cAMP responsive element binding protein 1	204314_s_at	-1.919	0.001	365	227	-1.6	1.1	-1.2
EXOC1	exocyst complex component 1	222127_s_at	-1.919	0.001	815	489	-1.7	1.3	-2.4
CD2AP	CD2 associated protein	203593_at	-1.918	0.001	363	175	-2.1	1.3	-1.0
TMEM19	transmembrane protein 19	229126_at	-1.915	0.001	469	292	-1.6	-1.2	1.1
AHR	aryl hydrocarbon receptor	202820_at	-1.913	0.001	1341	743	-1.8	1.1	2.0
APCDD1	APC down-regulated 1	225016_at	-1.912	0.001	2294	1201	-1.9	-2.1	-2.4
PRRC1	proline rich coiled-coil 1	224643_at	-1.911	0.001	885	585	-1.5	-1.1	1.0
JAG1	jagged canonical Notch ligand 1	216268_s_at	-1.909	0.001	1901	1091	-1.7	-1.1	-2.0
GID4	GID complex subunit 4 homolog	228452_at	-1.908	0.001	275	163	-1.7	-1.5	-1.5
ARL5B	ADP ribosylation factor like GTPase 5B	226345_at	-1.906	0.001	503	261	-1.9	1.2	2.7
MAN1A1	mannosidase alpha class 1A member 1	221760_at	-1.906	0.001	644	329	-2.0	-1.0	1.2
KLF5	Kruppel like factor 5	209212_s_at	-1.905	0.001	686	275	-2.5	-2.2	1.3
WFDC5	WAP four-disulfide core domain 5	242204_at	-1.903	0.001	873	371	-2.4	-1.9	1.0
STON2	stonin 2	227461_at	-1.903	0.001	277	123	-2.3	1.1	1.3
EBLN3P	endogenous Bornavirus like nucleoprotein 3, pseudogene	226635_at	-1.902	0.001	513	325	-1.6	1.1	1.0
SOWAHC	sosondowah ankyrin repeat domain family member C	227034_at	-1.899	0.001	964	465	-2.1	-1.1	1.8
EPS8L2	EPS8 like 2	218180_s_at	-1.899	0.001	400	193	-2.1	-1.1	-2.3
SERPINB5	serpin family B member 5	204855_at	-1.897	0.001	3069	1324	-2.3	-1.2	1.1
MED13L	mediator complex subunit 13L	212209_at	-1.896	0.001	535	354	-1.5	-1.3	-1.5
ELK4	ETS transcription factor ELK4	225159_s_at	-1.896	0.001	502	321	-1.6	-1.0	1.0
SYNCRIP	synaptotagmin binding cytoplasmic RNA interacting protein	217833_at	-1.894	0.001	707	451	-1.6	-1.6	1.2
PBX1	PBX homeobox 1	212151_at	-1.893	0.001	563	366	-1.5	-1.3	-1.4

CAPZA2	capping actin protein of muscle Z-line subunit alpha 2	201237_at	-1.893	0.001	488	289	-1.7	1.8	-1.2
MFAP3L	microfibril associated protein 3 like	205442_at	-1.892	0.001	609	272	-2.2	-2.2	-1.5
MXD1	MAX dimerization protein 1	226275_at	-1.891	0.001	321	166	-1.9	1.3	-1.3
CRLF3	cytokine receptor like factor 3	205474_at	-1.890	0.001	588	385	-1.5	1.4	1.5
ELL2	elongation factor for RNA polymerase II 2	226099_at	-1.888	0.001	628	303	-2.1	1.8	1.8
SLC31A2	solute carrier family 31 member 2	204204_at	-1.888	0.001	710	378	-1.9	-1.0	-1.0
KIAA1109	KIAA1109	212779_at	-1.888	0.001	464	299	-1.6	1.0	-2.4
KIAA1671	KIAA1671	225525_at	-1.887	0.001	592	322	-1.8	-1.7	1.5
PTGS1	prostaglandin-endoperoxide synthase 1	215813_s_at	-1.884	0.001	564	288	-2.0	-1.9	1.2
SS18	SS18 subunit of BAF chromatin remodeling complex	202817_s_at	-1.884	0.001	341	187	-1.8	1.5	-1.2
MUC15	mucin 15, cell surface associated	227238_at	-1.882	0.001	535	127	-4.2	1.1	1.2
BEX2	brain expressed X-linked 2	224367_at	-1.881	0.001	399	209	-1.9	-1.2	1.1
DSC1	desmocollin 1	207324_s_at	-1.879	0.001	3878	1129	-3.4	-1.3	1.1
TNFSF10	TNF superfamily member 10	202688_at	-1.878	0.001	1473	774	-1.9	1.7	-1.0
LRRC40	leucine rich repeat containing 40	218577_at	-1.878	0.001	421	225	-1.9	2.0	1.5
DSG3	desmoglein 3	235075_at	-1.877	0.001	945	403	-2.3	1.2	-1.2
ZNF750	zinc finger protein 750	219995_s_at	-1.876	0.001	805	276	-2.9	-1.3	-1.0
PIK3C2A	phosphatidylinositol-4-phosphate 3-kinase catalytic subunit type 2 alpha	213070_at	-1.876	0.001	599	371	-1.6	1.1	-1.5
TMEM79	transmembrane protein 79	223544_at	-1.875	0.001	665	289	-2.3	-1.1	1.1
APPL2	adaptor protein, phosphotyrosine interacting with PH domain and leucine zipper 2	218218_at	-1.874	0.001	439	229	-1.9	-1.1	1.2
WDR36	WD repeat domain 36	226180_at	-1.872	0.001	320	150	-2.1	-1.1	2.1
ZDHHC9	zinc finger DHHC-type containing 9	222451_s_at	-1.872	0.001	491	305	-1.6	-1.2	-1.2
CEBPA	CCAAT enhancer binding protein alpha	204039_at	-1.870	0.001	914	495	-1.8	-1.5	-1.1
ID2	inhibitor of DNA binding 2	201565_s_at	-1.869	0.001	1119	745	-1.5	1.2	-2.7
PPIP5K2	diphosphoinositol pentakisphosphate kinase 2	203253_s_at	-1.869	0.001	412	214	-1.9	1.9	-1.6
ZNF431	zinc finger protein 431	232338_at	-1.869	0.001	302	122	-2.5	2.2	1.4
OVOL1	ovo like transcriptional repressor 1	229396_at	-1.868	0.001	701	346	-2.0	-1.5	-1.1

FERMT1	fermitin family member 1	60474_at	-1.868	0.001	335	166	-2.0	-1.4	-1.0
DHX29	DExH-box helicase 29	212648_at	-1.867	0.001	515	338	-1.5	1.5	-1.2
ALDH3B2	aldehyde dehydrogenase 3 family member B2	204942_s_at	-1.867	0.001	1352	391	-3.5	-1.0	1.1
DAAM1	dishevelled associated activator of morphogenesis 1	216060_s_at	-1.866	0.001	570	278	-2.1	-1.3	-1.2
PPP2CA	protein phosphatase 2 catalytic subunit alpha	238719_at	-1.865	0.001	263	142	-1.8	-1.2	-1.1
TINCR	TINCR ubiquitin domain containing	229385_s_at	-1.865	0.001	464	155	-3.0	-2.2	-1.1
ARL4A	ADP ribosylation factor like GTPase 4A	205020_s_at	-1.864	0.001	800	505	-1.6	-1.0	2.0
MCC	MCC regulator of WNT signaling pathway	226225_at	-1.862	0.001	476	225	-2.1	-1.7	-2.8
MIER1	MIER1 transcriptional regulator	225475_at	-1.862	0.001	344	205	-1.7	1.8	-1.1
EPHB6	EPH receptor B6	204718_at	-1.860	0.001	511	178	-2.9	-2.1	-1.2
BICD2	BICD cargo adaptor 2	213154_s_at	-1.857	0.001	1183	668	-1.8	-1.1	-1.2
CLIC3	chloride intracellular channel 3	219529_at	-1.856	0.001	632	218	-2.9	-1.0	1.1
VSNL1	visinin like 1	203797_at	-1.854	0.001	570	241	-2.4	1.1	1.1
KDSR	3-ketodihydrosphingosine reductase	229850_at	-1.854	0.001	344	195	-1.8	-1.5	-2.2
SERBP1	SERPINE1 mRNA binding protein 1	227369_at	-1.851	0.001	477	252	-1.9	-1.2	2.4
CCND2	cyclin D2	200953_s_at	-1.851	0.001	950	486	-2.0	-1.5	-1.1
SPTSSA	serine palmitoyltransferase small subunit A	213508_at	-1.850	0.001	759	485	-1.6	-1.3	-1.8
ACAP2	ArfGAP with coiled-coil, ankyrin repeat and PH domains 2	212476_at	-1.850	0.001	550	357	-1.5	1.1	-1.2
FGL2	fibrinogen like 2	227265_at	-1.850	0.001	1393	655	-2.1	1.7	1.1
KITLG	KIT ligand	226534_at	-1.849	0.001	557	286	-1.9	1.2	5.0
REEP3	receptor accessory protein 3	225785_at	-1.849	0.001	472	300	-1.6	1.2	1.9
SOX7	SRY-box 7	228698_at	-1.849	0.001	760	328	-2.3	1.1	1.2
C1orf116	chromosome 1 open reading frame 116	219476_at	-1.848	0.001	523	196	-2.7	-1.5	1.1
BNIPL	BCL2 interacting protein like	236534_at	-1.848	0.001	479	163	-2.9	-1.3	1.0
SPTBN2	spectrin beta, non-erythrocytic 2	231311_at	-1.846	0.001	409	147	-2.8	-2.0	1.0
UBLCP1	ubiquitin like domain containing CTD phosphatase 1	227413_at	-1.845	0.001	558	315	-1.8	1.9	1.1

HLA-DQB2	major histocompatibility complex, class II, DQ beta 2	215536_at	-1.844	0.001	614	249	-2.5	-1.6	-1.6
CBR4	carbonyl reductase 4	213626_at	-1.844	0.001	277	146	-1.9	-1.4	-2.9
SOX9	SRY-box 9	202935_s_at	-1.842	0.001	978	318	-3.1	-1.9	-1.1
DOCK9	dedicator of cytokinesis 9	212538_at	-1.842	0.001	847	435	-1.9	1.3	-1.1
GPR87	G protein-coupled receptor 87	219936_s_at	-1.841	0.001	499	150	-3.3	1.2	-1.1
LMBRD2	LMBR1 domain containing 2	226779_at	-1.841	0.001	453	230	-2.0	1.4	1.0
PRPS2	phosphoribosyl pyrophosphate synthetase 2	230352_at	-1.841	0.001	394	222	-1.8	1.0	1.0
SORD	sorbitol dehydrogenase	201563_at	-1.839	0.001	823	371	-2.2	-1.2	-1.3
ZHX1	zinc fingers and homeoboxes 1	223214_s_at	-1.838	0.001	331	154	-2.2	1.3	-2.4
GALNT1	polypeptide N-acetylgalactosaminyltransferase 1	201724_s_at	-1.837	0.001	623	364	-1.7	1.2	-1.6
MOSPD1	motile sperm domain containing 1	1557455_s_at	-1.837	0.001	331	198	-1.7	1.2	ND
LYSMD3	LysM domain containing 3	226321_at	-1.833	0.001	255	117	-2.2	1.3	1.0
PDZK1IP1	PDZK1 interacting protein 1	219630_at	-1.832	0.001	685	323	-2.1	1.2	1.1
ASF1A	anti-silencing function 1A histone chaperone	203427_at	-1.831	0.001	333	164	-2.0	-1.1	-1.2
CALML5	calmodulin like 5	220414_at	-1.830	0.001	4377	1908	-2.3	1.4	-1.1
KRT2	keratin 2	207908_at	-1.829	0.001	5680	2068	-2.7	-2.1	-1.1
ACER1	alkaline ceramidase 1	1553929_at	-1.829	0.001	381	142	-2.7	-1.3	ND
PIAS1	protein inhibitor of activated STAT 1	217862_at	-1.828	0.001	516	340	-1.5	1.1	-1.9
FBXO45	F-box protein 45	225099_at	-1.826	0.001	708	372	-1.9	1.9	1.5
IL1RN	interleukin 1 receptor antagonist	212657_s_at	-1.825	0.001	1035	620	-1.7	-1.1	1.3
MAP7	microtubule associated protein 7	202890_at	-1.824	0.001	454	179	-2.5	1.1	1.0
ABCA12	ATP binding cassette subfamily A member 12	215465_at	-1.823	0.001	528	153	-3.5	1.3	-1.0
USP31	ubiquitin specific peptidase 31	226035_at	-1.821	0.001	621	396	-1.6	-1.1	1.4
TRPS1	transcriptional repressor GATA binding 1	222651_s_at	-1.816	0.001	642	389	-1.7	1.2	1.3
ATL2	atlastin GTPase 2	222700_at	-1.815	0.001	930	516	-1.8	1.6	2.5
CERS3	ceramide synthase 3	1554252_a_at	-1.814	0.001	407	140	-2.9	-1.4	ND
MIB1	mindbomb E3 ubiquitin protein ligase 1	224720_at	-1.811	0.001	444	227	-2.0	1.1	-1.1
JMJD1C	jumonji domain containing 1C	221763_at	-1.810	0.001	487	307	-1.6	1.2	1.5

ZNF644	zinc finger protein 644	222580_at	-1.810	0.001	436	288	-1.5	1.3	-1.1
BTBD11	BTB domain containing 11	228570_at	-1.809	0.001	248	110	-2.3	-1.4	1.2
AEBP2	AE binding protein 2	225889_at	-1.809	0.001	486	237	-2.0	1.1	1.2
AFDN	afadin, adherens junction formation factor	224685_at	-1.808	0.001	848	505	-1.7	1.1	2.0
FAM110C	family with sequence similarity 110 member C	226863_at	-1.806	0.001	316	100	-3.2	1.4	-1.3
CNIH1	cornichon family AMPA receptor auxiliary protein 1	225553_at	-1.804	0.001	556	370	-1.5	-1.5	1.1
CHP2	calcineurin like EF-hand protein 2	206149_at	-1.802	0.001	320	112	-2.9	-4.5	-1.1
STAM2	signal transducing adaptor molecule 2	209649_at	-1.802	0.001	345	190	-1.8	1.4	1.2
SGPL1	sphingosine-1-phosphate lyase 1	212321_at	-1.801	0.001	468	298	-1.6	-1.4	-1.6
PPP4R3A	protein phosphatase 4 regulatory subunit 3A	220368_s_at	-1.800	0.001	399	255	-1.6	1.1	1.0
CDKN2B	cyclin dependent kinase inhibitor 2B	236313_at	-1.800	0.001	442	134	-3.3	1.6	1.9
CD24	CD24 molecule	209771_x_at	-1.799	0.001	2616	1295	-2.0	2.0	-1.0
IFFO2	intermediate filament family orphan 2	225615_at	-1.798	0.001	895	489	-1.8	-1.5	1.9
CDK7	cyclin dependent kinase 7	211297_s_at	-1.797	0.001	375	244	-1.5	1.4	1.5
NDFIP1	Nedd4 family interacting protein 1	222423_at	-1.797	0.001	634	421	-1.5	-1.1	-2.8
FAM83B	family with sequence similarity 83 member B	232202_at	-1.797	0.001	599	205	-2.9	-1.5	1.1
EPHX3	epoxide hydrolase 3	220013_at	-1.795	0.001	611	261	-2.3	-1.1	-1.2
LYRM7	LYR motif containing 7	228841_at	-1.794	0.001	412	234	-1.8	-1.1	-1.3
NFAT5	nuclear factor of activated T cells 5	224984_at	-1.792	0.001	1001	618	-1.6	-1.2	1.1
ZRANB1	zinc finger RANBP2-type containing 1	225138_at	-1.792	0.001	312	190	-1.6	-1.1	-2.3
LAMP2	lysosomal associated membrane protein 2	226671_at	-1.792	0.001	396	208	-1.9	-1.8	-2.5
DDI2	DNA damage inducible 1 homolog 2	225780_at	-1.791	0.001	445	272	-1.6	1.2	-1.4
YAP1	Yes associated protein 1	224894_at	-1.788	0.001	1058	686	-1.5	-1.1	1.6
DENND2C	DENN domain containing 2C	230769_at	-1.788	0.001	279	110	-2.5	-1.1	-1.0
AKAP11	A-kinase anchoring protein 11	203156_at	-1.787	0.001	693	434	-1.6	1.1	-1.2
UEVLD	UEV and lactate/malate dehyrogenase domains	220775_s_at	-1.787	0.001	249	145	-1.7	-1.0	-1.5

MOB3B	MOB kinase activator 3B	226844_at	-1.786	0.001	279	168	-1.7	-2.2	-4.4
ELOVL4	ELOVL fatty acid elongase 4	219532_at	-1.785	0.001	788	153	-5.2	1.4	-1.0
UCHL3	ubiquitin C-terminal hydrolase L3	204616_at	-1.785	0.001	1328	783	-1.7	1.5	-1.1
SLC18B1	solute carrier family 18 member B1	226301_at	-1.784	0.001	802	291	-2.8	1.5	-9.1
CTNNBIP1	catenin beta interacting protein 1	203081_at	-1.784	0.001	747	478	-1.6	-1.8	1.1
SPAG9	sperm associated antigen 9	225339_at	-1.782	0.001	597	387	-1.5	-1.3	-1.8
DICER1	dicer 1, ribonuclease III	212888_at	-1.782	0.001	646	401	-1.6	1.1	-1.3
PPP1R13L	protein phosphatase 1 regulatory subunit 13 like	218849_s_at	-1.782	0.001	370	180	-2.1	-1.8	1.2
PPIC	peptidylprolyl isomerase C	204517_at	-1.780	0.001	895	565	-1.6	1.4	2.8
EMB	embigin	226789_at	-1.779	0.001	812	440	-1.8	-1.2	2.8
B3GNT5	UDP-GlcNAc:betaGal beta-1,3-N-acetylglucosaminyltransferase 5	225612_s_at	-1.778	0.001	454	198	-2.3	1.7	3.0
BAZ2B	bromodomain adjacent to zinc finger domain 2B	203080_s_at	-1.778	0.001	549	344	-1.6	1.5	-2.4
ZNF121	zinc finger protein 121	1553979_at	-1.777	0.001	623	410	-1.5	1.2	ND
DYNLT3	dynein light chain Tctex-type 3	203303_at	-1.774	0.001	1096	706	-1.6	1.5	1.6
LAMTOR3	late endosomal/lysosomal adaptor, MAPK and MTOR activator 3	217971_at	-1.773	0.001	692	451	-1.5	1.4	-1.9
PDZD8	PDZ domain containing 8	213549_at	-1.772	0.001	275	150	-1.8	-1.3	-1.0
GULP1	GULP PTB domain containing engulfment adaptor 1	204237_at	-1.768	0.001	492	234	-2.1	-1.0	3.1
KIF1B	kinesin family member 1B	226968_at	-1.767	0.001	486	294	-1.7	1.2	2.2
PPP4R3B	protein phosphatase 4 regulatory subunit 3B	233759_s_at	-1.766	0.001	561	372	-1.5	1.2	1.0
CCDC126	coiled-coil domain containing 126	228087_at	-1.766	0.001	220	115	-1.9	1.1	1.6
MYO6	myosin VI	203216_s_at	-1.765	0.001	511	221	-2.3	1.4	-1.8
CCNC	cyclin C	201955_at	-1.765	0.001	1156	726	-1.6	1.4	1.9
TGFBR3	transforming growth factor beta receptor 3	226625_at	-1.765	0.001	674	406	-1.7	-1.6	-1.7
TTC39C	tetratricopeptide repeat domain 39C	238480_at	-1.764	0.001	270	149	-1.8	-1.1	1.9
SFN	stratifin	209260_at	-1.763	0.001	1788	1033	-1.7	-1.4	1.1
EFNA3	ephrin A3	210132_at	-1.762	0.001	472	306	-1.5	-1.3	1.1

DNAJB14	DnaJ heat shock protein family (Hsp40) member B14	226399_at	-1.757	0.001	360	220	-1.6	1.4	-1.7
CYP2R1	cytochrome P450 family 2 subfamily R member 1	227109_at	-1.757	0.001	326	175	-1.9	1.2	1.0
MBTD1	mbt domain containing 1	226797_at	-1.756	0.001	366	183	-2.0	1.0	-1.0
SLC35F5	solute carrier family 35 member F5	225872_at	-1.756	0.001	560	316	-1.8	1.6	-1.3
RNF6	ring finger protein 6	203403_s_at	-1.754	0.001	768	464	-1.7	1.6	-2.1
RAB11A	RAB11A, member RAS oncogene family	200864_s_at	-1.753	0.001	654	413	-1.6	-1.1	-1.6
C5orf46	chromosome 5 open reading frame 46	1554195_a_at	-1.752	0.001	1007	264	-3.8	-1.8	ND
SRPK1	SRSF protein kinase 1	202200_s_at	-1.752	0.001	855	475	-1.8	1.3	1.6
CRABP2	cellular retinoic acid binding protein 2	202575_at	-1.752	0.001	1562	779	-2.0	1.3	-1.1
PRSS8	serine protease 8	202525_at	-1.751	0.001	440	156	-2.8	-1.2	1.2
KRT15	keratin 15	204734_at	-1.750	0.001	2526	410	-6.2	-3.4	2.1
ATP6V1C2	ATPase H+ transporting V1 subunit C2	1552532_a_at	-1.750	0.001	400	84	-4.7	2.2	ND
GOLGA4	golgin A4	201567_s_at	-1.749	0.001	1059	662	-1.6	1.7	1.1
ANK3	ankyrin 3	206385_s_at	-1.748	0.001	543	185	-2.9	-1.4	5.5
CCL27	C-C motif chemokine ligand 27	207955_at	-1.747	0.001	327	68	-4.8	-3.8	-1.1
PTPN11	protein tyrosine phosphatase non-receptor type 11	209896_s_at	-1.746	0.001	482	293	-1.6	-1.0	-1.3
CRYBG3	crystallin beta-gamma domain containing 3	214030_at	-1.745	0.001	441	259	-1.7	-1.1	-1.1
SERPINB7	serpin family B member 7	206421_s_at	-1.744	0.001	1270	443	-2.9	-1.0	1.1
PPA2	pyrophosphatase (inorganic) 2	1559496_at	-1.743	0.001	353	147	-2.4	1.4	ND
SNAI2	snail family transcriptional repressor 2	213139_at	-1.742	0.001	1427	855	-1.7	-1.8	-5.7
EEF1A1	eukaryotic translation elongation factor 1 alpha 1	227708_at	-1.739	0.001	630	390	-1.6	-1.9	-1.2
SLC16A7	solute carrier family 16 member 7	207057_at	-1.738	0.001	558	282	-2.0	1.0	-2.2
ZNF185	zinc finger protein 185 with LIM domain	203585_at	-1.737	0.001	493	262	-1.9	-1.4	-1.2
COX11	cytochrome c oxidase copper chaperone COX11	211727_s_at	-1.737	0.001	884	581	-1.5	1.0	1.7
HACD2	3-hydroxyacyl-CoA dehydratase 2	212640_at	-1.737	0.001	780	403	-1.9	-1.1	1.1
ARHGEF37	Rho guanine nucleotide exchange factor 37	227717_at	-1.737	0.001	483	189	-2.6	-2.7	-3.9

SMPDL3A	sphingomyelin phosphodiesterase acid like 3A	213624_at	-1.736	0.001	950	481	-2.0	-1.3	-3.6
C11orf54	chromosome 11 open reading frame 54	229851_s_at	-1.734	0.001	341	198	-1.7	1.3	1.2
TSTD1	thiosulfate sulfurtransferase like domain containing 1	226482_s_at	-1.733	0.001	634	287	-2.2	1.0	-2.6
CITED2	Cbp/p300 interacting transactivator with Glu/Asp rich carboxy-terminal domain 2	209357_at	-1.733	0.001	638	334	-1.9	-1.4	1.1
STK26	serine/threonine kinase 26	218499_at	-1.733	0.001	289	108	-2.7	-1.8	2.3
HSPB1	heat shock protein family B (small) member 1	201841_s_at	-1.733	0.001	6475	4217	-1.5	-1.1	-1.8
PHAX	phosphorylated adaptor for RNA export	235767_x_at	-1.732	0.001	316	195	-1.6	1.1	-1.5
FAM214A	family with sequence similarity 214 member A	225327_at	-1.730	0.001	472	277	-1.7	1.2	-2.8
C19orf33	chromosome 19 open reading frame 33	223631_s_at	-1.729	0.001	1005	288	-3.5	-1.2	-1.0
PPM1A	protein phosphatase, Mg2+/Mn2+ dependent 1A	227728_at	-1.729	0.001	352	196	-1.8	-1.2	-1.4
SLC40A1	solute carrier family 40 member 1	223044_at	-1.728	0.001	1546	806	-1.9	1.9	-1.4
PSORS1C2	psoriasis susceptibility 1 candidate 2	220635_at	-1.727	0.001	661	299	-2.2	-1.7	1.1
ZC3H12C	zinc finger CCCH-type containing 12C	231899_at	-1.727	0.001	359	210	-1.7	-1.0	1.2
GRHL3	grainyhead like transcription factor 3	232116_at	-1.726	0.001	379	158	-2.4	-1.1	1.2
TPPP3	tubulin polymerization promoting protein family member 3	218876_at	-1.725	0.001	312	145	-2.1	-3.1	-1.2
ATF1	activating transcription factor 1	222103_at	-1.725	0.001	402	258	-1.6	1.1	-1.2
PALLD	palladin, cytoskeletal associated protein	200907_s_at	-1.724	0.001	1412	823	-1.7	1.7	3.1
RELCH	RAB11 binding and LisH domain, coiled-coil and HEAT repeat containing	225508_at	-1.724	0.001	396	223	-1.8	1.4	-1.5
PELI1	pellino E3 ubiquitin protein ligase 1	218319_at	-1.723	0.001	651	404	-1.6	1.2	1.0
NTRK2	neurotrophic receptor tyrosine kinase 2	221796_at	-1.723	0.001	306	126	-2.4	-1.3	-1.1
PKP3	plakophilin 3	209873_s_at	-1.720	0.001	1004	500	-2.0	-1.5	1.1
ANKRD13C	ankyrin repeat domain 13C	223418_x_at	-1.719	0.001	262	138	-1.9	1.4	1.1
GGT6	gamma-glutamyltransferase 6	236225_at	-1.718	0.001	263	87	-3.0	-1.5	-1.3
MYLK	myosin light chain kinase	224823_at	-1.717	0.001	1485	907	-1.6	1.2	1.3
CALML3	calmodulin like 3	210020_x_at	-1.717	0.001	2746	954	-2.9	1.7	1.2

TMEM154	transmembrane protein 154	238063_at	-1.716	0.001	478	198	-2.4	-1.2	1.8
ARAP2	ArfGAP with RhoGAP domain, ankyrin repeat and PH domain 2	213618_at	-1.716	0.001	458	181	-2.5	1.5	-1.2
CTSV	cathepsin V	210074_at	-1.715	0.001	1867	580	-3.2	1.0	1.3
TUBA4A	tubulin alpha 4a	212242_at	-1.714	0.001	2062	1043	-2.0	-1.1	1.5
SREK1IP1	SREK1 interacting protein 1	235390_at	-1.714	0.001	319	173	-1.8	1.1	1.1
ZBTB21	zinc finger and BTB domain containing 21	225539_at	-1.714	0.001	301	151	-2.0	-1.0	-1.1
PSMC6	proteasome 26S subunit, ATPase 6	201699_at	-1.714	0.001	704	464	-1.5	1.8	1.2
KLC3	kinesin light chain 3	239853_at	-1.714	0.001	439	184	-2.4	-2.0	-1.2
ASAP3	ArfGAP with SH3 domain, ankyrin repeat and PH domain 3	222236_s_at	-1.711	0.001	391	249	-1.6	-1.3	-1.2
EPS8L1	EPS8 like 1	218779_x_at	-1.711	0.001	337	173	-1.9	-1.2	-1.5
ACVR2A	activin A receptor type 2A	228416_at	-1.710	0.001	280	123	-2.3	-1.6	1.4
RDH13	retinol dehydrogenase 13	225467_s_at	-1.710	0.001	459	269	-1.7	1.0	-1.2
MIR205HG	MIR205 host gene	226755_at	-1.710	0.001	277	120	-2.3	-1.1	-1.1
SH3D19	SH3 domain containing 19	225162_at	-1.709	0.001	498	257	-1.9	-1.5	-1.2
SGMS2	sphingomyelin synthase 2	227038_at	-1.709	0.001	185	81	-2.3	1.3	2.2
ARG1	arginase 1	206177_s_at	-1.709	0.001	1807	746	-2.4	2.0	-1.1
SNHG19	small nucleolar RNA host gene 19	228049_x_at	-1.709	0.001	655	397	-1.6	-1.8	1.3
HPGD	15-hydroxyprostaglandin dehydrogenase	203914_x_at	-1.708	0.001	1126	481	-2.3	-1.8	-5.5
C21orf91	chromosome 21 open reading frame 91	226109_at	-1.708	0.001	1104	488	-2.3	2.0	-9.3
CMTM4	CKLF like MARVEL transmembrane domain containing 4	225009_at	-1.708	0.001	696	444	-1.6	-1.2	-1.7
SLC10A3	solute carrier family 10 member 3	204928_s_at	-1.707	0.001	324	215	-1.5	-1.1	1.4
TOPORS	TOP1 binding arginine/serine rich protein	204071_s_at	-1.706	0.001	322	205	-1.6	1.4	-1.1
BDP1	B double prime 1, subunit of RNA polymerase III transcription initiation factor IIIB	226290_at	-1.706	0.001	349	188	-1.9	1.2	1.2
SCYL2	SCY1 like pseudokinase 2	224961_at	-1.706	0.001	439	266	-1.6	1.1	-1.1
PTGER3	prostaglandin E receptor 3	213933_at	-1.704	0.001	442	171	-2.6	1.3	1.0
HLF	HLF transcription factor, PAR bZIP family member	204753_s_at	-1.703	0.001	180	68	-2.7	-2.1	-1.3
IL22RA1	interleukin 22 receptor subunit alpha 1	220056_at	-1.702	0.001	243	114	-2.1	-1.5	-1.1

BAG4	BCL2 associated athanogene 4	228189_at	-1.699	0.001	387	249	-1.6	-1.4	1.0
SLC22A23	solute carrier family 22 member 23	223194_s_at	-1.698	0.001	398	238	-1.7	-1.2	1.1
CWH43	cell wall biogenesis 43 C-terminal homolog	220723_s_at	-1.698	0.001	592	208	-2.8	1.2	1.1
KDM5B	lysine demethylase 5B	201548_s_at	-1.698	0.001	447	291	-1.5	-1.1	-1.4
HMGCR	3-hydroxy-3-methylglutaryl-CoA reductase	202540_s_at	-1.698	0.001	775	342	-2.3	1.1	1.0
CRIM1	cysteine rich transmembrane BMP regulator 1	202551_s_at	-1.697	0.001	534	304	-1.8	-1.3	5.2
AIMP1	aminoacyl tRNA synthetase complex interacting multifunctional protein 1	202541_at	-1.696	0.001	342	213	-1.6	1.3	1.2
S100A7	S100 calcium binding protein A7	205916_at	-1.695	0.001	9043	6010	-1.5	4.6	-1.0
EIF4E3	eukaryotic translation initiation factor 4E family member 3	225941_at	-1.695	0.001	240	137	-1.7	-1.3	-1.1
IRX5	iroquois homeobox 5	210239_at	-1.695	0.001	348	164	-2.1	-1.3	-1.7
CYLD	CYLD lysine 63 deubiquitinase	213295_at	-1.695	0.001	328	202	-1.6	-1.0	-1.4
RANBP6	RAN binding protein 6	213019_at	-1.695	0.001	341	209	-1.6	1.0	-1.5
ZNF430	zinc finger protein 430	206829_x_at	-1.694	0.001	543	355	-1.5	1.3	1.0
STAP2	signal transducing adaptor family member 2	221610_s_at	-1.693	0.001	450	208	-2.2	1.0	1.0
RANGAP1	Ran GTPase activating protein 1	212125_at	-1.692	0.001	375	242	-1.5	-1.1	2.1
ABHD5	abhydrolase domain containing 5	213805_at	-1.692	0.001	567	133	-4.3	1.5	-1.1
ME1	malic enzyme 1	204059_s_at	-1.692	0.001	1502	552	-2.7	1.7	3.4
SLC30A4	solute carrier family 30 member 4	227193_at	-1.691	0.001	326	196	-1.7	1.1	1.0
C15orf48	chromosome 15 open reading frame 48	223484_at	-1.690	0.001	1002	381	-2.6	1.0	1.1
TRIM13	tripartite motif containing 13	229943_at	-1.686	0.001	570	343	-1.7	1.1	-1.2
SMPD3	sphingomyelin phosphodiesterase 3	219695_at	-1.686	0.001	369	146	-2.5	1.1	-1.0
RDH12	retinol dehydrogenase 12	242998_at	-1.684	0.001	485	246	-2.0	-1.3	-1.0
RCOR3	REST corepressor 3	222605_at	-1.683	0.001	486	319	-1.5	-1.1	-3.1
S100A2	S100 calcium binding protein A2	204268_at	-1.682	0.001	4615	2728	-1.7	1.8	1.6
DUOX1	dual oxidase 1	219597_s_at	-1.682	0.001	519	201	-2.6	1.0	1.1
ZBTB7C	zinc finger and BTB domain containing 7C	227782_at	-1.681	0.001	496	249	-2.0	-1.2	1.0

ACSL1	acyl-CoA synthetase long chain family member 1	201963_at	-1.680	0.001	2000	996	-2.0	-1.0	-3.1
FEM1B	fem-1 homolog B	212367_at	-1.678	0.001	434	258	-1.7	1.2	-3.1
FBXO28	F-box protein 28	202271_at	-1.677	0.001	834	503	-1.7	1.0	1.4
RECQL	RecQ like helicase	212917_x_at	-1.676	0.001	624	407	-1.5	2.5	2.0
FAT2	FAT atypical cadherin 2	208153_s_at	-1.674	0.001	259	102	-2.5	-1.8	-1.0
NIPAL4	NIPA like domain containing 4	230188_at	-1.674	0.001	460	270	-1.7	-1.4	1.2
ITM2A	integral membrane protein 2A	202746_at	-1.672	0.001	554	276	-2.0	-1.3	1.1
IPO7	importin 7	200995_at	-1.672	0.001	306	176	-1.7	-1.1	1.6
IRX3	iroquois homeobox 3	229638_at	-1.671	0.001	1561	702	-2.2	-1.4	-1.3
AJUBA	ajuba LIM protein	225806_at	-1.671	0.001	256	124	-2.1	-2.4	3.3
DDHD1	DDHD domain containing 1	225971_at	-1.668	0.001	287	157	-1.8	1.9	-1.2
CHURC1	churchill domain containing 1	223210_at	-1.668	0.001	490	291	-1.7	1.2	-2.1
ATMIN	ATM interactor	201855_s_at	-1.668	0.001	434	268	-1.6	-1.1	-3.8
MTURN	maturin, neural progenitor differentiation regulator homolog	226018_at	-1.667	0.001	290	139	-2.1	-2.0	-10.2
PAQR3	progestin and adipoQ receptor family member 3	213372_at	-1.666	0.001	216	93	-2.3	1.4	-1.1
FAM83C	family with sequence similarity 83 member C	1556793_a_at	-1.665	0.001	630	240	-2.6	-1.3	ND
TPD52L1	TPD52 like 1	203786_s_at	-1.664	0.001	1149	610	-1.9	-1.2	1.4
IL18	interleukin 18	206295_at	-1.664	0.001	557	138	-4.0	-1.0	1.1
ITGA2	integrin subunit alpha 2	227314_at	-1.664	0.001	325	154	-2.1	-1.1	3.5
HOOK3	hook microtubule tethering protein 3	226395_at	-1.663	0.001	389	213	-1.8	-1.0	-1.1
LAD1	ladinin 1	203287_at	-1.660	0.001	749	395	-1.9	-1.3	-1.1
MAOA	monoamine oxidase A	212741_at	-1.659	0.001	472	208	-2.3	-1.6	-1.2
TMOD3	tropomodulin 3	223077_at	-1.659	0.001	466	284	-1.6	1.2	1.2
ARHGAP29	Rho GTPase activating protein 29	203910_at	-1.657	0.001	563	320	-1.8	1.6	2.9
ALG13	ALG13 UDP-N-acetylglucosaminyltransferase subunit	222808_at	-1.657	0.001	324	209	-1.5	1.3	1.5
USP15	ubiquitin specific peptidase 15	210681_s_at	-1.656	0.001	481	315	-1.5	2.1	1.5
PLXNB1	plexin B1	215807_s_at	-1.655	0.001	381	244	-1.6	-1.2	-3.1
PRKCI	protein kinase C iota	209678_s_at	-1.655	0.001	1191	757	-1.6	1.3	1.2

TWF1	twinfilin actin binding protein 1	201745_at	-1.653	0.001	784	507	-1.5	2.0	-3.8
TMEM33	transmembrane protein 33	225492_at	-1.653	0.001	673	435	-1.5	1.1	-1.1
TLE4	TLE family member 4, transcriptional corepressor	204872_at	-1.650	0.001	331	159	-2.1	-1.7	2.0
KRT77	keratin 77	237120_at	-1.649	0.001	729	166	-4.4	-4.1	1.1
PDCD6IP	programmed cell death 6 interacting protein	222394_at	-1.647	0.001	460	276	-1.7	-1.3	-1.8
ZDHHC13	zinc finger DHHC-type containing 13	219296_at	-1.647	0.001	269	135	-2.0	1.2	1.4
SGPP2	sphingosine-1-phosphate phosphatase 2	226560_at	-1.646	0.001	935	241	-3.9	-1.0	1.2
CAMSAP2	calmodulin regulated spectrin associated protein family member 2	212765_at	-1.644	0.001	678	401	-1.7	-1.0	-1.4
TTC22	tetratricopeptide repeat domain 22	235651_at	-1.641	0.001	440	187	-2.4	-1.3	1.1
PRXL2A	peroxiredoxin like 2A	228155_at	-1.641	0.001	2200	1229	-1.8	-1.4	-1.6
GNA13	G protein subunit alpha 13	224761_at	-1.641	0.001	2349	1524	-1.5	1.3	-1.4
ATP10D	ATPase phospholipid transporting 10D (putative)	213238_at	-1.638	0.001	500	267	-1.9	1.2	-1.6
MARCH3	membrane associated ring-CH-type finger 3	213256_at	-1.638	0.001	347	170	-2.0	1.1	-1.1
FBXW7	F-box and WD repeat domain containing 7	229419_at	-1.637	0.001	245	116	-2.1	-1.6	-1.1
CHMP4C	charged multivesicular body protein 4C	226803_at	-1.633	0.001	347	132	-2.6	-1.2	-1.0
EXOC8	exocyst complex component 8	227577_at	-1.632	0.001	299	178	-1.7	1.6	1.1
RCOR1	REST corepressor 1	212612_at	-1.631	0.001	371	241	-1.5	1.1	-1.4
JAG2	jagged canonical Notch ligand 2	209784_s_at	-1.630	0.001	442	280	-1.6	-1.5	-1.6
ELOVL1	ELOVL fatty acid elongase 1	57163_at	-1.622	0.001	1050	646	-1.6	1.2	1.5
TTC37	tetratricopeptide repeat domain 37	203048_s_at	-1.622	0.001	466	310	-1.5	1.4	-1.5
MCEE	methylmalonyl-CoA epimerase	226238_at	-1.621	0.001	249	148	-1.7	1.2	-1.2
TBL1XR1	transducin beta like 1 X-linked receptor 1	221428_s_at	-1.620	0.001	313	188	-1.7	-1.0	1.0
KLK10	kallikrein related peptidase 10	209792_s_at	-1.620	0.001	1287	589	-2.2	1.1	-1.1
GCH1	GTP cyclohydrolase 1	204224_s_at	-1.619	0.001	388	193	-2.0	2.1	-1.0
SLMAP	sarcolemma associated protein	225243_s_at	-1.619	0.001	440	262	-1.7	1.1	-1.3
PGGT1B	protein geranylgeranyltransferase type I subunit beta	235615_at	-1.618	0.001	218	113	-1.9	1.3	-1.0

PPP2R5E	protein phosphatase 2 regulatory subunit B'epsilon	227630_at	-1.617	0.001	296	194	-1.5	-1.5	-1.1
LAMA3	laminin subunit alpha 3	203726_s_at	-1.617	0.001	304	185	-1.6	-1.3	1.2
FAR1	fatty acyl-CoA reductase 1	224866_at	-1.616	0.001	372	234	-1.6	1.6	-1.0
SLC46A2	solute carrier family 46 member 2	223816_at	-1.616	0.001	183	66	-2.8	-1.5	1.2
COMMD8	COMM domain containing 8	218351_at	-1.616	0.001	937	622	-1.5	2.0	-1.5
OTUB2	OTU deubiquitinase, ubiquitin aldehyde binding 2	219369_s_at	-1.615	0.001	298	134	-2.2	1.1	1.1
TPRG1	tumor protein p63 regulated 1	229764_at	-1.614	0.001	457	180	-2.5	-1.2	-1.0
SRSF11	serine and arginine rich splicing factor 11	200685_at	-1.613	0.001	320	175	-1.8	1.3	-1.1
NCOA2	nuclear receptor coactivator 2	212867_at	-1.612	0.001	568	370	-1.5	-1.0	-1.3
RAP2C	RAP2C, member of RAS oncogene family	218668_s_at	-1.612	0.001	319	200	-1.6	2.0	1.2
SPPL2A	signal peptide peptidase like 2A	227682_at	-1.612	0.001	301	162	-1.9	-1.1	1.6
DEFB1	defensin beta 1	210397_at	-1.612	0.001	909	263	-3.5	1.0	1.1
PALMD	palmdelphin	218736_s_at	-1.611	0.001	682	294	-2.3	-1.1	1.2
RC3H1	ring finger and CCCH-type domains 1	225893_at	-1.611	0.001	530	335	-1.6	1.0	-1.2
MPP7	membrane palmitoylated protein 7	238778_at	-1.610	0.001	183	46	-4.0	-1.4	1.0
SPTLC3	serine palmitoyltransferase long chain base subunit 3	227752_at	-1.610	0.001	274	91	-3.0	1.1	-1.3
TGFA	transforming growth factor alpha	205016_at	-1.609	0.001	309	145	-2.1	1.1	30.6
ACPP	acid phosphatase, prostate	204393_s_at	-1.608	0.001	625	274	-2.3	1.4	1.9
BBOX1	gamma-butyrobetaine hydroxylase 1	205363_at	-1.608	0.001	482	174	-2.8	-1.3	1.2
CASZ1	castor zinc finger 1	243386_at	-1.605	0.001	343	90	-3.8	1.0	-1.1
DAPL1	death associated protein like 1	229290_at	-1.604	0.001	1763	363	-4.9	1.1	1.1
SPINT1	serine peptidase inhibitor, Kunitz type 1	202826_at	-1.601	0.001	685	386	-1.8	-1.4	-1.1
FKBP5	FKBP prolyl isomerase 5	224840_at	-1.597	0.001	1756	995	-1.8	1.7	1.6
ERBIN	erbb2 interacting protein	222473_s_at	-1.597	0.001	425	278	-1.5	1.2	-2.4
PRDM1	PR/SET domain 1	228964_at	-1.597	0.001	738	435	-1.7	1.3	1.4
PSAPL1	prosaposin like 1 (gene/pseudogene)	1564333_a_at	-1.597	0.001	1460	416	-3.5	-1.4	ND
SRD5A1	steroid 5 alpha-reductase 1	204675_at	-1.596	0.001	862	210	-4.1	-1.1	-1.2
MTF1	metal regulatory transcription factor 1	227150_at	-1.596	0.001	344	218	-1.6	1.3	-1.0
CCL18	C-C motif chemokine ligand 18	32128_at	-1.595	0.001	4098	928	-4.4	12.0	-2.0
TSPAN13	tetraspanin 13	217979_at	-1.594	0.001	707	387	-1.8	1.9	12.7

MAP4K5	mitogen-activated protein kinase kinase kinase kinase 5	203552_at	-1.593	0.001	322	194	-1.7	1.0	-1.1
NCOA7	nuclear receptor coactivator 7	225344_at	-1.592	0.001	939	594	-1.6	1.6	1.6
HIP1R	huntingtin interacting protein 1 related	38340_at	-1.591	0.001	542	357	-1.5	-1.2	-1.1
FOXQ1	forkhead box Q1	227475_at	-1.590	0.001	600	241	-2.5	-3.0	-1.6
IL11RA	interleukin 11 receptor subunit alpha	204773_at	-1.589	0.001	314	200	-1.6	-1.7	-2.4
EPN3	epsin 3	223895_s_at	-1.589	0.001	182	73	-2.5	1.2	-1.4
SEC24A	SEC24 homolog A, COPII coat complex component	212900_at	-1.589	0.001	588	358	-1.6	1.4	2.5
PCGF5	polycomb group ring finger 5	226326_at	-1.587	0.001	260	155	-1.7	-1.1	-1.0
PTK6	protein tyrosine kinase 6	206482_at	-1.587	0.001	374	157	-2.4	-1.1	-1.0
ACOX1	acyl-CoA oxidase 1	227962_at	-1.587	0.001	449	286	-1.6	-1.3	-1.5
MANSC1	MANSC domain containing 1	220945_x_at	-1.584	0.001	735	387	-1.9	1.1	1.4
DNASE1L2	deoxyribonuclease 1 like 2	207192_at	-1.584	0.001	371	134	-2.8	-1.0	-1.0
WDR47	WD repeat domain 47	203855_at	-1.584	0.001	420	266	-1.6	-1.3	1.1
CARD18	caspase recruitment domain family member 18	231733_at	-1.582	0.001	857	241	-3.6	1.6	1.3
FAM241A	family with sequence similarity 241 member A	227856_at	-1.582	0.001	357	197	-1.8	1.1	1.5
MBOAT2	membrane bound O-acyltransferase domain containing 2	213288_at	-1.581	0.001	600	349	-1.7	-1.0	1.1
MARVELD2	MARVEL domain containing 2	235141_at	-1.579	0.001	219	108	-2.0	-1.3	1.3
GLRX	glutaredoxin	206662_at	-1.578	0.001	1504	905	-1.7	1.9	1.4
IREB2	iron responsive element binding protein 2	225892_at	-1.578	0.001	361	232	-1.6	1.3	1.1
UBN2	ubinuclein 2	225444_at	-1.576	0.001	323	206	-1.6	-1.1	-1.1
EFNB2	ephrin B2	202669_s_at	-1.576	0.001	495	213	-2.3	-1.2	1.6
PHF20L1	PHD finger protein 20 like 1	226942_at	-1.575	0.001	390	237	-1.6	1.7	-1.1
MAPK13	mitogen-activated protein kinase 13	210058_at	-1.575	0.001	604	370	-1.6	-1.1	1.2
ABHD17B	abhydrolase domain containing 17B	227551_at	-1.572	0.001	395	246	-1.6	-1.2	-1.6
TIFA	TRAF interacting protein with forkhead associated domain	226117_at	-1.572	0.001	387	249	-1.6	1.3	1.8
GAB1	GRB2 associated binding protein 1	226002_at	-1.572	0.001	237	136	-1.7	-1.3	-1.4

XIST	X inactive specific transcript	224588_at	-1.571	0.001	1418	38	-37.7	-2.1	1.0
ETNK1	ethanolamine kinase 1	242059_at	-1.571	0.001	420	199	-2.1	3.2	-1.1
NCK1	NCK adaptor protein 1	211063_s_at	-1.570	0.001	626	410	-1.5	1.5	-1.4
TNS4	tensin 4	230398_at	-1.570	0.001	365	215	-1.7	-1.6	1.1
TMEM159	transmembrane protein 159	213272_s_at	-1.570	0.001	281	161	-1.7	-2.0	-1.5
KIAA0513	KIAA0513	204546_at	-1.569	0.001	254	151	-1.7	1.1	-1.5
NFIL3	nuclear factor, interleukin 3 regulated	203574_at	-1.569	0.001	378	246	-1.5	1.1	-2.0
PTN	pleiotrophin	211737_x_at	-1.568	0.001	522	284	-1.8	-6.1	4.7
DEPTOR	DEP domain containing MTOR interacting protein	218858_at	-1.568	0.001	366	222	-1.6	-1.3	-1.1
CPA4	carboxypeptidase A4	205832_at	-1.567	0.001	449	240	-1.9	-1.1	-1.1
TMPRSS13	transmembrane serine protease 13	223659_at	-1.566	0.001	275	175	-1.6	-1.3	-1.2
ACBD5	acyl-CoA binding domain containing 5	225663_at	-1.566	0.001	530	345	-1.5	1.2	-3.0
GRHL2	grainyhead like transcription factor 2	219388_at	-1.564	0.001	283	118	-2.4	-1.6	1.0
MBNL3	muscleblind like splicing regulator 3	229498_at	-1.556	0.001	443	261	-1.7	-1.1	1.4
CUL4A	cullin 4A	201424_s_at	-1.556	0.001	510	333	-1.5	1.3	-1.3
EVPL	envoplakin	204503_at	-1.555	0.001	311	163	-1.9	-1.6	1.1
ALDH5A1	aldehyde dehydrogenase 5 family member A1	203608_at	-1.554	0.001	340	167	-2.0	1.1	-1.1
KCTD1	potassium channel tetramerization domain containing 1	226245_at	-1.554	0.001	548	321	-1.7	-1.7	1.0
C16orf72	chromosome 16 open reading frame 72	225197_at	-1.554	0.001	321	213	-1.5	-1.2	1.6
VANGL2	VANGL planar cell polarity protein 2	226029_at	-1.553	0.001	318	181	-1.8	-1.0	-2.2
LRP11	LDL receptor related protein 11	225060_at	-1.550	0.001	574	344	-1.7	1.1	1.2
GRB7	growth factor receptor bound protein 7	210761_s_at	-1.550	0.001	258	154	-1.7	-1.1	-1.1
NFIA	nuclear factor I A	224970_at	-1.546	0.001	614	401	-1.5	-1.5	-1.3
BLNK	B cell linker	207655_s_at	-1.546	0.001	478	242	-2.0	2.4	-1.1
ESRP2	epithelial splicing regulatory protein 2	219395_at	-1.546	0.001	344	168	-2.1	-1.0	1.1
C11orf80	chromosome 11 open reading frame 80	238593_at	-1.545	0.001	185	71	-2.6	1.5	1.5
NR1D2	nuclear receptor subfamily 1 group D member 2	225768_at	-1.544	0.001	314	199	-1.6	2.0	-1.1
DR1	down-regulator of transcription 1	209187_at	-1.542	0.001	692	443	-1.6	1.1	-1.1
PRSS3	serine protease 3	207463_x_at	-1.541	0.001	543	254	-2.1	1.2	1.1

TRIM16	tripartite motif containing 16	204341_at	-1.540	0.001	365	227	-1.6	-1.1	2.6
TNFAIP8	TNF alpha induced protein 8	208296_x_at	-1.537	0.001	604	389	-1.6	1.8	1.5
TMEM30B	transmembrane protein 30B	213285_at	-1.537	0.001	223	109	-2.0	-1.2	1.0
CHMP2B	charged multivesicular body protein 2B	202536_at	-1.535	0.001	453	298	-1.5	1.5	-2.1
DAPP1	dual adaptor of phosphotyrosine and 3-phosphoinositides 1	222858_s_at	-1.535	0.001	380	216	-1.8	1.6	1.1
SCIN	scinderin	1552365_at	-1.534	0.001	322	29	-11.1	5.0	ND
PPP6R3	protein phosphatase 6 regulatory subunit 3	222467_s_at	-1.533	0.001	429	274	-1.6	1.2	-1.0
ZNF451	zinc finger protein 451	212557_at	-1.532	0.001	387	251	-1.5	1.4	-1.5
TFAP2C	transcription factor AP-2 gamma	205286_at	-1.531	0.001	854	537	-1.6	-1.4	4.2
PGRMC1	progesterone receptor membrane component 1	201121_s_at	-1.531	0.001	2041	1296	-1.6	1.0	1.7
WFDC21P	WAP four-disulfide core domain 21, pseudogene	229566_at	-1.531	0.001	524	272	-1.9	-1.1	1.1
IL1R2	interleukin 1 receptor type 2	205403_at	-1.531	0.001	342	215	-1.6	-1.6	1.1
YIPF5	Yip1 domain family member 5	224953_at	-1.531	0.001	454	280	-1.6	-1.3	-1.4
STARD5	StAR related lipid transfer domain containing 5	213820_s_at	-1.530	0.001	320	188	-1.7	-1.5	-1.2
FZD3	frizzled class receptor 3	239082_at	-1.526	0.001	285	154	-1.9	-1.2	-1.9
DENND2D	DENN domain containing 2D	221081_s_at	-1.526	0.001	412	228	-1.8	1.8	1.1
APOOL	apolipoprotein O like	213282_at	-1.525	0.001	311	207	-1.5	-1.2	-1.5
APPL1	adaptor protein, phosphotyrosine interacting with PH domain and leucine zipper 1	218158_s_at	-1.525	0.001	269	165	-1.6	1.2	1.1
ATP9A	ATPase phospholipid transporting 9A (putative)	212062_at	-1.522	0.001	443	281	-1.6	1.2	3.5
CYB5A	cytochrome b5 type A	215726_s_at	-1.522	0.001	1208	542	-2.2	1.1	1.3
DDX5	DEAD-box helicase 5	225886_at	-1.522	0.001	400	267	-1.5	-1.1	-1.9
C9orf16	chromosome 9 open reading frame 16	41047_at	-1.521	0.001	1236	821	-1.5	-1.2	-1.2
CACNA2D1	calcium voltage-gated channel auxiliary subunit alpha2delta 1	227623_at	-1.520	0.001	231	110	-2.1	-1.2	1.1
BSPRY	B-box and SPRY domain containing	222746_s_at	-1.519	0.001	274	128	-2.1	-1.3	-1.0

MALAT1	metastasis associated lung adenocarcinoma transcript 1	224558_s_at	-1.518	0.001	1421	876	-1.6	1.7	-1.7
LYNX1	Ly6/neurotoxin 1	1554179_s_at	-1.517	0.001	1675	784	-2.1	1.2	ND
STARD10	StAR related lipid transfer domain containing 10	223103_at	-1.517	0.001	358	223	-1.6	-1.3	-4.1
TET2	tet methylcytosine dioxygenase 2	227624_at	-1.516	0.001	256	136	-1.9	1.1	-1.0
MBNL2	muscleblind like splicing regulator 2	203640_at	-1.515	0.001	1043	650	-1.6	-1.3	1.1
SLC16A10	solute carrier family 16 member 10	222939_s_at	-1.515	0.001	269	138	-2.0	-1.7	-2.0
AADACL2	arylacetamide deacetylase like 2	240420_at	-1.513	0.001	511	124	-4.1	1.7	-1.0
PNLIPRP3	pancreatic lipase related protein 3	1558846_at	-1.513	0.001	701	130	-5.4	2.6	ND
EGR2	early growth response 2	205249_at	-1.513	0.001	613	345	-1.8	1.4	-1.1
DUOXA1	dual oxidase maturation factor 1	1554648_a_at	-1.511	0.001	340	130	-2.6	1.2	ND
PER2	period circadian regulator 2	205251_at	-1.511	0.001	587	386	-1.5	-1.2	1.2
C18orf25	chromosome 18 open reading frame 25	226406_at	-1.510	0.001	311	206	-1.5	1.0	1.0
ALDH3A1	aldehyde dehydrogenase 3 family member A1	205623_at	-1.510	0.001	212	97	-2.2	-1.7	-1.0
IRF2BP2	interferon regulatory factor 2 binding protein 2	224571_at	-1.509	0.001	528	352	-1.5	-1.2	-1.4
FZD6	frizzled class receptor 6	203987_at	-1.508	0.001	698	462	-1.5	1.3	5.7
ERMP1	endoplasmic reticulum metallopeptidase 1	222603_at	-1.508	0.001	282	144	-2.0	-1.2	-3.1
RHOT1	ras homolog family member T1	218323_at	-1.506	0.001	517	344	-1.5	1.1	-1.3
SLC39A10	solute carrier family 39 member 10	225295_at	-1.504	0.001	413	236	-1.7	1.2	3.0
UBE2D3	ubiquitin conjugating enzyme E2 D3	240383_at	-1.504	0.001	369	185	-2.0	3.9	-1.1
TMEM41B	transmembrane protein 41B	212622_at	-1.504	0.001	523	315	-1.7	-1.0	1.3
MSMO1	methylsterol monooxygenase 1	209146_at	-1.502	0.001	2250	780	-2.9	1.4	2.7
CPD	carboxypeptidase D	201941_at	-1.502	0.001	584	366	-1.6	1.1	1.3
GOLT1B	golgi transport 1B	222552_at	-1.500	0.001	750	463	-1.6	1.8	2.8
LRRC8B	leucine rich repeat containing 8 VRAC subunit B	212978_at	-1.499	0.001	300	188	-1.6	1.1	-1.1
ZNF292	zinc finger protein 292	212368_at	-1.499	0.001	824	531	-1.6	1.2	-1.1
MBLAC2	metallo-beta-lactamase domain containing 2	230298_at	-1.498	0.001	256	148	-1.7	-1.3	-1.1

ABI3BP	ABI family member 3 binding protein	223395_at	-1.497	0.001	725	441	-1.6	-1.0	1.5
IL37	interleukin 37	221470_s_at	-1.496	0.001	218	56	-3.9	-2.2	1.1
DLG1	discs large MAGUK scaffold protein 1	202514_at	-1.495	0.001	315	189	-1.7	-1.1	-3.2
LPCAT2	lysophosphatidylcholine acyltransferase 2	227889_at	-1.495	0.001	368	180	-2.0	-1.5	2.2
ANK2	ankyrin 2	202920_at	-1.495	0.001	654	433	-1.5	-1.9	-2.7
ANXA9	annexin A9	210085_s_at	-1.493	0.001	286	162	-1.8	-1.2	1.0
TST	thiosulfate sulfurtransferase	209605_at	-1.492	0.001	496	256	-1.9	1.2	-1.2
SPRR4	small proline rich protein 4	1552620_at	-1.492	0.001	2040	595	-3.4	-1.1	ND
RABGAP1L	RAB GTPase activating protein 1 like	203020_at	-1.492	0.001	353	214	-1.6	1.0	-1.2
SPRR1A	small proline rich protein 1A	214549_x_at	-1.491	0.001	3550	1998	-1.8	2.7	-1.1
CYP4F22	cytochrome P450 family 4 subfamily F member 22	244692_at	-1.489	0.001	390	116	-3.3	1.3	1.3
RSF1	remodeling and spacing factor 1	235381_at	-1.484	0.001	464	295	-1.6	1.1	1.1
ANO1	anoctamin 1	218804_at	-1.483	0.001	267	140	-1.9	-2.1	1.2
CD207	CD207 molecule	220428_at	-1.483	0.001	381	175	-2.2	-1.8	-1.0
SEH1L	SEH1 like nucleoporin	221931_s_at	-1.482	0.001	701	466	-1.5	-1.0	2.8
KIZ	kizuna centrosomal protein	219961_s_at	-1.482	0.001	317	191	-1.7	1.0	-1.8
SLC39A6	solute carrier family 39 member 6	202089_s_at	-1.480	0.001	1479	918	-1.6	-1.2	-4.8
CNFN	cornifelin	224329_s_at	-1.478	0.001	2022	921	-2.2	1.9	-1.0
LYPD6B	LY6/PLAUR domain containing 6B	228360_at	-1.477	0.001	208	97	-2.1	-1.9	1.4
BICDL1	BICD family like cargo adaptor 1	228320_x_at	-1.476	0.001	238	133	-1.8	-1.3	-1.7
MYC	MYC proto-oncogene, bHLH transcription factor	202431_s_at	-1.475	0.001	1751	1037	-1.7	-1.2	-1.4
GRAMD2B	GRAM domain containing 2B	218706_s_at	-1.469	0.001	437	282	-1.5	-1.1	-1.4
FOXN1	forkhead box N1	1558687_a_at	-1.466	0.001	292	101	-2.9	-1.6	ND
GRAMD1C	GRAM domain containing 1C	219313_at	-1.463	0.001	198	49	-4.1	1.8	1.0
PMVK	phosphomevalonate kinase	203515_s_at	-1.459	0.001	637	343	-1.9	1.0	1.2
LY75	lymphocyte antigen 75	205668_at	-1.456	0.001	279	134	-2.1	1.3	-1.1
TIAM1	T cell lymphoma invasion and metastasis 1	213135_at	-1.455	0.001	923	536	-1.7	-1.1	2.1
ZBTB43	zinc finger and BTB domain containing 43	227991_x_at	-1.455	0.001	310	193	-1.6	-1.1	1.1
KIAA1522	KIAA1522	224746_at	-1.454	0.001	626	392	-1.6	-1.5	-1.2

CYSRT1	cysteine rich tail 1	1569144_a_at	-1.453	0.001	382	197	-1.9	1.0	ND
DPY19L4	dpy-19 like 4	226721_at	-1.453	0.001	257	150	-1.7	-1.1	1.7
SLURP1	secreted LY6/PLAUR domain containing 1	214536_at	-1.452	0.001	1849	598	-3.1	1.0	1.0
COL7A1	collagen type VII alpha 1 chain	204136_at	-1.452	0.001	322	195	-1.7	-1.1	-1.1
LRRC1	leucine rich repeat containing 1	218816_at	-1.450	0.001	229	116	-2.0	-1.8	1.1
ALCAM	activated leukocyte cell adhesion molecule	201952_at	-1.449	0.001	804	459	-1.8	1.2	4.1
FAM167A	family with sequence similarity 167 member A	226614_s_at	-1.449	0.001	279	148	-1.9	-1.4	1.6
DHCR24	24-dehydrocholesterol reductase	200862_at	-1.448	0.001	2240	844	-2.7	-1.2	5.0
OCIAD2	OCIA domain containing 2	225314_at	-1.447	0.001	474	309	-1.5	1.1	1.1
POU2F3	POU class 2 homeobox 3	207109_at	-1.446	0.001	184	43	-4.3	-2.0	1.0
ZNF91	zinc finger protein 91	206059_at	-1.445	0.001	1025	469	-2.2	1.5	-1.4
PPP1R14C	protein phosphatase 1 regulatory inhibitor subunit 14C	226907_at	-1.443	0.001	592	372	-1.6	-1.8	1.1
KCTD12	potassium channel tetramerization domain containing 12	212192_at	-1.442	0.001	1567	1042	-1.5	1.3	-1.2
SLC15A1	solute carrier family 15 member 1	207254_at	-1.442	0.001	243	84	-2.9	1.5	-1.1
NIBAN1	niban apoptosis regulator 1	217967_s_at	-1.441	0.001	885	581	-1.5	1.2	10.4
DGAT2	diacylglycerol O-acyltransferase 2	226064_s_at	-1.441	0.001	1345	283	-4.8	-1.2	2.0
MTX3	metaxin 3	226528_at	-1.441	0.001	360	235	-1.5	1.2	1.6
MGST1	microsomal glutathione S-transferase 1	224918_x_at	-1.440	0.001	2607	1086	-2.4	1.4	1.1
SOSTDC1	sclerostin domain containing 1	213456_at	-1.439	0.001	466	163	-2.9	-1.7	1.1
CPSF6	cleavage and polyadenylation specific factor 6	226934_at	-1.437	0.001	229	114	-2.0	-1.0	1.5
PRDX2	peroxiredoxin 2	39729_at	-1.436	0.001	2100	1377	-1.5	1.1	-1.9
ZNF662	zinc finger protein 662	228538_at	-1.433	0.001	216	89	-2.4	1.2	-1.1
PKN2	protein kinase N2	212628_at	-1.432	0.001	290	179	-1.6	-1.0	-1.0
ZEB2	zinc finger E-box binding homeobox 2	228333_at	-1.431	0.001	385	249	-1.5	-1.4	-3.9
HOMER2	homer scaffold protein 2	217080_s_at	-1.430	0.001	335	178	-1.9	1.0	-1.1
EBP	EBP cholestenol delta-isomerase	202735_at	-1.429	0.001	458	288	-1.6	1.1	1.2
PEX3	peroxisomal biogenesis factor 3	203972_s_at	-1.426	0.001	550	270	-2.0	1.3	1.1
LPAR1	lysophosphatidic acid receptor 1	204036_at	-1.425	0.001	374	230	-1.6	-2.2	1.4

DOP1A	DOP1 leucine zipper like protein A	213271_s_at	-1.424	0.001	297	196	-1.5	-1.0	-1.9
EPB41L4B	erythrocyte membrane protein band 4.1 like 4B	220161_s_at	-1.423	0.001	310	151	-2.1	-1.5	1.2
RAB7B	RAB7B, member RAS oncogene family	230266_at	-1.422	0.001	272	163	-1.7	-1.2	2.0
PPP4R2	protein phosphatase 4 regulatory subunit 2	226317_at	-1.422	0.001	341	224	-1.5	1.1	-1.4
IFRD1	interferon related developmental regulator 1	202146_at	-1.421	0.001	426	276	-1.5	1.8	-1.1
ZADH2	zinc binding alcohol dehydrogenase domain containing 2	227049_at	-1.420	0.001	381	252	-1.5	-1.3	-1.7
RPS23	ribosomal protein S23	227722_at	-1.419	0.001	406	239	-1.7	-1.9	-1.3
NOD2	nucleotide binding oligomerization domain containing 2	220066_at	-1.419	0.001	267	158	-1.7	1.7	1.1
PREPL	prolyl endopeptidase like	212215_at	-1.418	0.001	413	275	-1.5	-1.2	-2.3
SCOC	short coiled-coil protein	223341_s_at	-1.418	0.001	296	190	-1.6	1.9	-1.1
FAM199X	family with sequence similarity 199, X-linked	225216_at	-1.418	0.001	344	227	-1.5	1.0	1.1
ELL3	elongation factor for RNA polymerase II 3	219517_at	-1.418	0.001	292	185	-1.6	-1.2	-1.1
LTN1	listerin E3 ubiquitin protein ligase 1	215596_s_at	-1.417	0.001	489	322	-1.5	1.3	1.1
RHOU	ras homolog family member U	223168_at	-1.417	0.001	331	194	-1.7	2.4	1.3
PMAIP1	phorbol-12-myristate-13-acetate-induced protein 1	204285_s_at	-1.417	0.001	728	419	-1.7	1.9	4.1
MOB1B	MOB kinase activator 1B	225997_at	-1.412	0.001	429	267	-1.6	1.2	-1.3
LUC7L3	LUC7 like 3 pre-mRNA splicing factor	229193_at	-1.412	0.001	244	111	-2.2	1.9	-1.1
GNPAT	glyceronephosphate O-acyltransferase	201956_s_at	-1.411	0.001	974	599	-1.6	1.2	-1.2
DYNC1LI1	dynein cytoplasmic 1 light intermediate chain 1	217976_s_at	-1.411	0.001	377	240	-1.6	1.2	-1.5
PCMTD2	protein-L-isoaspartate (D-aspartate) O-methyltransferase domain containing 2	212406_s_at	-1.411	0.001	1097	590	-1.9	-1.0	-5.7
ECHDC2	enoyl-CoA hydratase domain containing 2	218552_at	-1.409	0.001	782	424	-1.8	-1.1	-1.3
ARHGAP5	Rho GTPase activating protein 5	217936_at	-1.408	0.001	490	308	-1.6	1.2	-1.4
METTL7A	methyltransferase like 7A	207761_s_at	-1.408	0.001	1370	825	-1.7	1.0	-1.3

LINC00302	long intergenic non-protein coding RNA 302	216935_at	-1.408	0.001	469	182	-2.6	-1.3	1.0
HCAR3	hydroxycarboxylic acid receptor 3	205220_at	-1.405	0.001	460	235	-2.0	-1.0	-1.1
TM7SF2	transmembrane 7 superfamily member 2	210130_s_at	-1.404	0.001	541	190	-2.8	-1.0	1.2
ALOX12B	arachidonate 12-lipoxygenase, 12R type	207381_at	-1.404	0.001	549	237	-2.3	1.1	-1.0
IGFL2	IGF like family member 2	231148_at	-1.404	0.001	563	223	-2.5	1.2	1.0
RNASE7	ribonuclease A family member 7	234700_s_at	-1.403	0.001	280	110	-2.6	1.4	-1.1
VSIG10L	V-set and immunoglobulin domain containing 10 like	238654_at	-1.403	0.001	529	244	-2.2	1.1	-1.7
SERPINB3	serpin family B member 3	209719_x_at	-1.402	0.001	3479	1979	-1.8	6.3	1.1
LRIG1	leucine rich repeats and immunoglobulin like domains 1	211596_s_at	-1.399	0.001	237	126	-1.9	-1.8	1.1
HMGCS1	3-hydroxy-3-methylglutaryl-CoA synthase 1	221750_at	-1.398	0.001	461	152	-3.0	1.1	1.3
CSNK1G3	casein kinase 1 gamma 3	220768_s_at	-1.398	0.001	371	239	-1.6	1.4	-1.3
ZNF493	zinc finger protein 493	1558486_at	-1.397	0.001	297	136	-2.2	1.5	ND
VDR	vitamin D receptor	204254_s_at	-1.394	0.001	366	240	-1.5	-1.1	1.2
TMEM254	transmembrane protein 254	222545_s_at	-1.394	0.001	409	230	-1.8	-1.3	-1.1
PDLIM5	PDZ and LIM domain 5	212412_at	-1.392	0.001	1137	753	-1.5	1.3	1.5
FAM171B	family with sequence similarity 171 member B	227370_at	-1.390	0.001	261	130	-2.0	1.2	-1.2
FBXL16	F-box and leucine rich repeat protein 16	227641_at	-1.388	0.001	294	175	-1.7	-1.6	-1.7
ISOC1	isochorismatase domain containing 1	218170_at	-1.387	0.001	556	272	-2.0	-1.3	1.2
TMEM45B	transmembrane protein 45B	226226_at	-1.387	0.001	378	199	-1.9	1.4	1.2
FAM185A	family with sequence similarity 185 member A	1556373_a_at	-1.385	0.001	350	194	-1.8	1.7	ND
GPSM2	G protein signaling modulator 2	230002_at	-1.379	0.001	481	244	-2.0	-1.2	1.5
PNISR	PNN interacting serine and arginine rich protein	212179_at	-1.378	0.001	406	257	-1.6	1.1	-2.3
PPIF	peptidylprolyl isomerase F	201490_s_at	-1.378	0.001	634	412	-1.5	1.7	2.2
CLIP4	CAP-Gly domain containing linker protein family member 4	226425_at	-1.377	0.001	390	208	-1.9	1.1	-1.1
PSME4	proteasome activator subunit 4	212222_at	-1.376	0.001	285	181	-1.6	1.2	1.1

MUCL1	mucin like 1	1553602_at	-1.376	0.001	3555	1633	-2.2	1.1	ND
RBM47	RNA binding motif protein 47	218035_s_at	-1.376	0.001	587	248	-2.4	1.3	1.1
FRY	FRY microtubule binding protein	204072_s_at	-1.375	0.001	273	166	-1.6	1.2	-2.4
NECTIN4	nectin cell adhesion molecule 4	223540_at	-1.375	0.001	440	242	-1.8	-1.4	1.0
TRPM1	transient receptor potential cation channel subfamily M member 1	237070_at	-1.374	0.001	710	296	-2.4	-1.5	-10.3
CYB561A3	cytochrome b561 family member A3	224735_at	-1.374	0.001	786	499	-1.6	-1.5	-11.0
TGM1	transglutaminase 1	206008_at	-1.373	0.001	529	287	-1.8	1.3	-1.0
PXMP4	peroxisomal membrane protein 4	219428_s_at	-1.373	0.001	574	346	-1.7	-1.0	-1.0
DUSP16	dual specificity phosphatase 16	224832_at	-1.371	0.001	513	248	-2.1	1.2	-1.4
INTS6	integrator complex subunit 6	218819_at	-1.371	0.001	594	393	-1.5	1.7	-1.1
DSC2	desmocollin 2	226817_at	-1.371	0.001	1454	906	-1.6	2.9	1.0
CA2	carbonic anhydrase 2	209301_at	-1.370	0.001	800	393	-2.0	-1.0	-1.1
AHNAK	AHNAK nucleoprotein	220016_at	-1.369	0.001	294	152	-1.9	-1.8	-1.4
COBLL1	cordon-bleu WH2 repeat protein like 1	203642_s_at	-1.369	0.001	265	153	-1.7	-1.1	-1.2
PARD3	par-3 family cell polarity regulator	210094_s_at	-1.368	0.001	346	227	-1.5	-1.4	1.4
C1orf68	chromosome 1 open reading frame 68	217087_at	-1.366	0.001	486	203	-2.4	-2.8	1.2
HS3ST6	heparan sulfate-glucosamine 3-sulfotransferase 6	239547_at	-1.365	0.001	166	64	-2.6	-1.5	-1.3
CDA	cytidine deaminase	205627_at	-1.365	0.001	330	148	-2.2	2.0	1.3
SERPINB2	serpin family B member 2	204614_at	-1.365	0.001	774	496	-1.6	-1.0	17.6
DGKH	diacylglycerol kinase eta	227415_at	-1.361	0.001	261	158	-1.6	1.5	1.5
RNF39	ring finger protein 39	219916_s_at	-1.361	0.001	370	219	-1.7	-1.4	1.1
PANK1	pantothenate kinase 1	226649_at	-1.361	0.001	331	97	-3.4	1.1	1.2
AKR1C3	aldo-keto reductase family 1 member C3	209160_at	-1.360	0.001	348	214	-1.6	1.5	1.1
IL20RA	interleukin 20 receptor subunit alpha	219115_s_at	-1.357	0.001	168	37	-4.6	-1.6	1.0
CDC73	cell division cycle 73	235196_at	-1.355	0.001	344	222	-1.6	1.1	1.3
FNIP1	folliculin interacting protein 1	228250_at	-1.354	0.001	329	202	-1.6	1.4	-1.0
ENDOU	endonuclease, poly(U) specific	206605_at	-1.352	0.001	289	90	-3.2	-1.3	-1.3
ELOVL5	ELOVL fatty acid elongase 5	208788_at	-1.351	0.001	2511	1371	-1.8	1.4	1.4
TFAP2A	transcription factor AP-2 alpha	204653_at	-1.351	0.001	3089	2052	-1.5	-1.1	-12.5
NMU	neuromedin U	206023_at	-1.349	0.001	515	178	-2.9	-1.5	1.0
SC5D	sterol-C5-desaturase	211423_s_at	-1.348	0.001	771	362	-2.1	1.7	1.0

SLC25A43	solute carrier family 25 member 43	1557411_s_at	-1.348	0.001	339	220	-1.5	-1.1	ND
SLC6A14	solute carrier family 6 member 14	219795_at	-1.347	0.001	192	57	-3.4	2.6	1.0
CD1C	CD1c molecule	205987_at	-1.346	0.001	342	196	-1.7	-1.3	1.1
DDX52	DExD-box helicase 52	213637_at	-1.345	0.001	352	224	-1.6	1.7	-1.0
CLU	clusterin	208791_at	-1.343	0.001	583	359	-1.6	-1.0	-1.1
LYPD5	LY6/PLAUR domain containing 5	236039_at	-1.343	0.001	238	136	-1.7	-1.3	1.1
DMD	dystrophin	203881_s_at	-1.341	0.001	251	127	-2.0	-2.0	1.3
RALGAPA1	Ral GTPase activating protein catalytic alpha subunit 1	213049_at	-1.341	0.001	437	291	-1.5	-1.0	-1.9
SERPINB13	serpin family B member 13	217272_s_at	-1.340	0.001	1385	562	-2.5	2.9	1.3
KRT17	keratin 17	212236_x_at	-1.339	0.001	7348	4774	-1.5	1.8	1.2
ANKRD44	ankyrin repeat domain 44	226641_at	-1.337	0.001	308	197	-1.6	2.2	-3.3
ATP11B	ATPase phospholipid transporting 11B (putative)	212536_at	-1.333	0.001	439	280	-1.6	1.1	-1.1
MAP2	microtubule associated protein 2	225540_at	-1.333	0.001	556	288	-1.9	-2.1	2.3
MYO5B	myosin VB	225301_s_at	-1.332	0.001	491	283	-1.7	-1.1	1.2
CFD	complement factor D	205382_s_at	-1.328	0.001	1262	674	-1.9	-1.3	1.1
CRYBG2	crystallin beta-gamma domain containing 2	220289_s_at	-1.327	0.001	209	102	-2.0	1.0	-1.0
YOD1	YOD1 deubiquitinase	227309_at	-1.327	0.001	838	370	-2.3	1.5	1.2
AGL	amylo-alpha-1, 6-glucosidase, 4-alpha-glucanotransferase	203566_s_at	-1.326	0.001	449	276	-1.6	1.6	-1.3
FCGBP	Fc fragment of IgG binding protein	203240_at	-1.326	0.001	575	301	-1.9	-1.5	-1.0
CD36	CD36 molecule	206488_s_at	-1.325	0.001	859	516	-1.7	7.0	-2.6
SLC2A1	solute carrier family 2 member 1	201250_s_at	-1.325	0.001	520	340	-1.5	-1.4	1.8
ACADM	acyl-CoA dehydrogenase medium chain	202502_at	-1.325	0.001	1250	679	-1.8	1.2	-1.3
SLC44A1	solute carrier family 44 member 1	224596_at	-1.325	0.001	1522	997	-1.5	1.1	-2.7
NQO2	N-ribosyldihydronicotinamide:quinone reductase 2	203814_s_at	-1.325	0.001	865	550	-1.6	1.1	-1.5
KCNK7	potassium two pore domain channel subfamily K member 7	224055_x_at	-1.320	0.001	204	102	-2.0	-1.9	1.3
BOK	BCL2 family apoptosis regulator BOK	223349_s_at	-1.320	0.001	306	200	-1.5	1.1	-1.0

ADTRP	androgen dependent TFPI regulating protein	229070_at	-1.319	0.001	551	135	-4.1	1.3	-1.1
MPZL2	myelin protein zero like 2	203780_at	-1.318	0.001	622	286	-2.2	2.6	-1.0
CDC42	cell division cycle 42	208727_s_at	-1.317	0.001	1593	952	-1.7	1.4	-1.1
HOOK1	hook microtubule tethering protein 1	225792_at	-1.316	0.001	301	115	-2.6	1.4	1.0

Only probesets with unique annotations are shown.

ND, not determined
