# Supplementary material for: Prolyl 4‐hydroxylase subunit alpha 1 (P4HA1) is a biomarker of poor prognosis in primary melanomas, and its depletion inhibits melanoma cell invasion and disrupts tumor blood vessel walls
Source: Mol Oncol. 2020 Feb 28;14(4):742–62. doi: 10.1002/1878-0261.12649 (PMC7138405; doi:10.1002/1878-0261.12649)
Supplement: Supplementary file 19 — Table S4. Gene Set Enrichment Analysis results for genes associated most significantly with patient survival. [file MOL2-14-742-s019.pdf]

Table S4. Gene Set Enrichment Analysis results for genes associated most significantly with patient survival.

	Gene set collection	Gene set	Normalized enrichment score	False discovery rate q-value
Genes associated with short survival	HALLMARK	EPITHELIAL_MESENCHYMAL_TRANSITION	3.454	0.000
		COAGULATION	2.882	0.000
		COMPLEMENT	2.083	0.015
	KEGG	FOCAL_ADHESION	2.316	0.008
		ECM_RECEPTOR_INTERACTION	2.314	0.004
		LYSOSOME	2.038	0.011
		REGULATION_OF_ACTIN_CYTOSKELETON	1.920	0.016
	REACTOME	EXTRACELLULAR_MATRIX_ORGANIZATION	3.125	0.000
		COLLAGEN_FORMATION	2.668	0.001
		DIABETES_PATHWAYS	2.638	0.000
		METABOLISM_OF_CARBOHYDRATES	2.571	0.000
		PLATELET_ACTIVATION_SIGNALING_AND_ AGGREGATION	2.328	0.002
		RESPONSE_TO_ELEVATED_PLATELET_CYTOSOLIC_CA2	2.154	0.009
		HEMOSTASIS	1.896	0.038
		INTEGRIN_CELL_SURFACE_INTERACTIONS	1.837	0.050
	GO_ BIOLOGICAL PROCESS	ANGIOGENESIS	2.817	0.002
		PLATELET_DEGRANULATION	2.790	0.002
		SINGLE_ORGANISM_CATABOLIC_PROCESS	2.687	0.003
		EXTRACELLULAR_STRUCTURE_ORGANIZATION	2.589	0.004
		MULTICELLULAR_ORGANISM_METABOLIC_PROCESS	2.505	0.007
		MULTICELLULAR_ORGANISMAL_MACROMOLECULE_ METABOLIC_PROCESS	2.443	0.011
		BLOOD_VESSEL_MORPHOGENESIS	2.404	0.014
		RESPONSE_TO_TOPOLOGICALLY_INCORRECT_PROTEIN	2.341	0.021
		REGENERATION	2.311	0.024
		REGULATED_EXOCYTOSIS	2.286	0.025
		RECEPTOR_MEDIATED_ENDOCYTOSIS	2.215	0.039
		EXOCYTOSIS	2.207	0.037
Genes associated with long survival	HALLMARK	ADIPOGENESIS	-2.037	0.039
	GO BIOLOGICAL PROCESS	EPIDERMAL_CELL_DIFFERENTIATION	-2.751	0.002
		SKIN_DEVELOPMENT	-2.683	0.002
		EPIDERMIS_DEVELOPMENT	-2.681	0.001
		KERATINOCYTE_DIFFERENTIATION	-2.681	0.001
		PROTEIN_LOCALIZATION_TO_CELL_PERIPHERY	-2.320	0.021
		POSITIVE_REGULATION_OF_TRANSCRIPTION_FROM_ RNA_POLYMERASE_II_PROMOTER	-2.233	0.032
