# Supplementary material for: Prolyl 4‐hydroxylase subunit alpha 1 (P4HA1) is a biomarker of poor prognosis in primary melanomas, and its depletion inhibits melanoma cell invasion and disrupts tumor blood vessel walls
Source: Mol Oncol. 2020 Feb 28;14(4):742–62. doi: 10.1002/1878-0261.12649 (PMC7138405; doi:10.1002/1878-0261.12649)
Supplement: Supplementary file 20 — Table S5. Kaplan‐Meier survival analysis and mean survival times of patients with primary melanomas that show low and high mRNA expression of the top short survival marker genes in an independent RNA sequencing data set (GSE98394). [file MOL2-14-742-s020.pdf]

Table S5. Kaplan-Meier survival analysis and mean survival times of patients with primary melanomas that show low and high mRNA expression of the top short survival marker genes in an independent RNA sequencing data set (GSE98394).

Gene		Mean survival time (months ± SE)	Log rank test p-value
CNP	Low mRNA expression	102.2 ± 7.3	0.03
	High mRNA expression	75.4 ± 9.7	
P4HA1	Low mRNA expression	113.5 ± 4.8	<0.001
	High mRNA expression	64.0 ± 9.2	
RHOC	Low mRNA expression	114.2 ± 4.2	<0.001
	High mRNA expression	63.2 ± 9.3	
SORT1	Low mRNA expression	110.0 ± 5.5	<0.001
	High mRNA expression	67.5 ± 9.6	
CD63	Low mRNA expression	113.7 ± 4.6	<0.001
	High mRNA expression	63.8 ± 9.3	
CDK2AP1	Low mRNA expression	112.3 ± 4.5	<0.001
	High mRNA expression	65.3 ± 9.7	
BIRC7	Low mRNA expression	107.6 ± 6.2	0.004
	High mRNA expression	69.6 ± 9.6	
BIN3	Low mRNA expression	106.0 ± 6.6	0.005
	High mRNA expression	71.5 ± 9.7	
ARHGEF40	Low mRNA expression	104.1 ± 6.7	0.025
	High mRNA expression	73.4 ± 9.9	
SHC1	Low mRNA expression	105.6 ± 6.8	0.006
	High mRNA expression	72.0 ± 9.6	
FN1	Low mRNA expression	107.7 ± 6.6	0.001
	High mRNA expression	69.8 ± 9.4	
TLNRD1	Low mRNA expression	105.0 ± 6.5	0.022
	High mRNA expression	72.0 ± 9.8	
ARPC1B	Low mRNA expression	110.7 ± 6.4	<0.001[†]
	High mRNA expression	66.9 ± 8.9	
PSMB4	Low mRNA expression	113.5 ± 5.3	<0.001[†]
	High mRNA expression	63.7 ± 8.9	
FKBP11	Low mRNA expression	107.5 ± 6.7	0.001
	High mRNA expression	70.0 ± 9.3	
COL9A3	Low mRNA expression	107.5 ± 6.0	0.004
	High mRNA expression	70.0 ± 9.8	
GNG5	Low mRNA expression	103.8 ± 6.9	0.024
	High mRNA expression	73.7 ± 9.8	
ETV5	Low mRNA expression	101.3 ± 7.7	0.039
	High mRNA expression	76.2 ± 9.5	
TYR	Low mRNA expression	109.0 ± 5.9	0.001
	High mRNA expression	68.4 ± 9.5	
SOX10	Low mRNA expression	101.6 ± 7.5	0.034
	High mRNA expression	75.9 ± 9.6	
PSRC1	Low mRNA expression	104.5 ± 6.7	0.029
	High mRNA expression	73.1 ± 9.8	
DUSP4	Low mRNA expression	110.4 ± 5.4	0.001
	High mRNA expression	67.2 ± 9.6	
BACE2	Low mRNA expression	105.5 ± 6.9	0.007
	High mRNA expression	72.1 ± 9.5	

[†]curves cross
