# Supplementary material for: Prolyl 4‐hydroxylase subunit alpha 1 (P4HA1) is a biomarker of poor prognosis in primary melanomas, and its depletion inhibits melanoma cell invasion and disrupts tumor blood vessel walls
Source: Mol Oncol. 2020 Feb 28;14(4):742–62. doi: 10.1002/1878-0261.12649 (PMC7138405; doi:10.1002/1878-0261.12649)
Supplement: Supplementary file 21 — Table S6. Kaplan‐Meier survival analysis and mean survival times of patients with primary melanomas that show low and high mRNA expression of the top long survival marker genes in an independent RNA sequencing data set (GSE98394). [file MOL2-14-742-s021.pdf]

Table S6. Kaplan-Meier survival analysis and mean survival times of patients with primary melanomas that show low and high mRNA expression of the top long survival marker genes in an independent RNA sequencing data set (GSE98394).

Gene		Mean survival time (months ± SE)	Log rank test p-value
KRT10	Low mRNA expression	66.7 ± 9.4	0.001
	High mRNA expression	110.5 ± 5.7	
FOXN3	Low mRNA expression	64.4 ± 9.5	<0.001
	High mRNA expression	112.8 ± 4.4	
MTSS1	Low mRNA expression	67.4 ± 9.4	0.001†
	High mRNA expression	110.1 ± 5.9	
REEP5	Low mRNA expression	69.0 ± 9.6	0.004
	High mRNA expression	108.2 ± 6.1	
CAST	Low mRNA expression	67.1 ± 9.9	0.002
	High mRNA expression	110.0 ± 4.9	
CCDC6	Low mRNA expression	72.9 ± 9.7	0.026
	High mRNA expression	104.1 ± 6.9	
DMKN	Low mRNA expression	76.7 ± 9.5	0.039
	High mRNA expression	100.7 ± 7.7	
EGFR	Low mRNA expression	60.2 ± 9.3	<0.001
	High mRNA expression	117.0 ± 2.1	
TUFT1	Low mRNA expression	72.0 ± 9.5	0.007
	High mRNA expression	105.5 ± 6.9	
DSC3	Low mRNA expression	67.0 ± 9.6	<0.001
	High mRNA expression	110.5 ± 5.3	
PERP	Low mRNA expression	74.1 ± 9.4	0.010
	High mRNA expression	103.4 ± 7.5	
HOPX	Low mRNA expression	78.2 ± 9.3	0.047
	High mRNA expression	99.3 ± 8.2	
TM9SF3	Low mRNA expression	70.5 ± 9.5	0.005
	High mRNA expression	106.6 ± 6.5	
CLDN1	Low mRNA expression	64.2 ± 9.5	<0.001
	High mRNA expression	112.9 ± 4.3	
PTBP3	Low mRNA expression	67.0 ± 9.6	<0.001
	High mRNA expression	110.4 ± 5.4	
NR3C1	Low mRNA expression	69.1 ± 10.1	0.013
	High mRNA expression	108.0 ± 5.3	
RIOK3	Low mRNA expression	72.9 ± 9.7	0.029
	High mRNA expression	104.1 ± 6.9	
CXADR	Low mRNA expression	63.8 ± 9.6	<0.001
	High mRNA expression	113.3 ± 4.0	
SLK	Low mRNA expression	69.2 ± 9.5	0.001
	High mRNA expression	108.3 ± 6.3	
KRT5	Low mRNA expression	72.5 ± 9.7	0.025
	High mRNA expression	104.6 ± 6.7	
TACSTD2	Low mRNA expression	76.7 ± 9.5	0.039
	High mRNA expression	100.7 ± 7.7	

†curves cross
