# Supplementary material for: Prolyl 4‐hydroxylase subunit alpha 1 (P4HA1) is a biomarker of poor prognosis in primary melanomas, and its depletion inhibits melanoma cell invasion and disrupts tumor blood vessel walls
Source: Mol Oncol. 2020 Feb 28;14(4):742–62. doi: 10.1002/1878-0261.12649 (PMC7138405; doi:10.1002/1878-0261.12649)
Supplement: Supplementary file 22 — Table S7. Expression levels of genes encoding collagen domain‐containing proteins in 62 melanoma cell lines (E‐GEOD‐7127). [file MOL2-14-742-s022.pdf]

**Table S7.** Expression levels of genes encoding collagen domain-containing proteins in 62 melanoma cell lines (E-GEOD-7127).

| Gene | Gene description | Probe set ID | Mean ±StDev<br>Melanoma cell lines (*n* = 62) | Fold<br>Melanoma cells* vs melanocytes |
|---|---|---|---|---|
| *CTHRC1* | Collagen triple helix repeat containing 1 | 225681_at | 3759 ± 2908 | 85.1 |
| *COL4A2* | Collagen, type IV, alpha 2 | 211964_at | 1587 ± 1573 | 2.0 |
| *COL1A2* | Collagen, type I, alpha 2 | 202403_s_at | 1179 ± 2429 | 1.0 |
| *COL5A2* | Collagen, type V, alpha 2 | 221729_at | 1013 ± 1353 | 1.6 |
| *COL4A1* | Collagen, type IV, alpha 1 | 211980_at | 929 ± 1100 | 2.8 |
| *COL9A3* | Collagen, type IX, alpha 3 | 204724_s_at | 844 ± 999 | 11.4 |
| *COL11A1* | Collagen, type XI, alpha 1 | 37892_at | 768 ± 1532 | -1.5 |
| *WDR33* | WD repeat domain 33 | 222763_s_at | 656 ± 276 | 1.1 |
| *COL6A1* | Collagen, type VI, alpha 1 | 213428_s_at | 650 ± 953 | 1.9 |
| *COL12A1* | Collagen, type XII, alpha 1 | 225664_at | 487 ± 1519 | 12.0 |
| *COL15A1* | Collagen, type XV, alpha 1 | 203477_at | 416 ± 1098 | 8.5 |
| *COL8A1* | Collagen, type VIII, alpha 1 | 226237_at | 402 ± 956 | 16.7 |
| *COL6A2* | Collagen, type VI, alpha 2 | 209156_s_at | 303 ± 474 | 3.1 |
| *COL18A1* | Collagen, type XVIII, alpha 1 | 209081_s_at | 277 ± 266 | -3.2 |
| *EMILIN2* | Elastin microfibril interfacer 2 | 224374_s_at | 273 ± 254 | 1.1 |
| *COL3A1* | Collagen, type III, alpha 1 | 215076_s_at | 270 ± 1433 | 1.0 |
| *COL16A1* | Collagen, type XVI, alpha 1 | 204345_at | 265 ± 238 | -1.1 |
| *COL27A1* | Collagen, type XXVII, alpha 1 | 225293_at | 256 ± 335 | -2.6 |
| *COL1A1* | Collagen, type I, alpha 1 | 1556499_s_at | 240 ± 1103 | ND |
| *COL13A1* | Collagen, type XIII, alpha 1 | 211343_s_at | 221 ± 423 | 4.6 |
| *COL7A1* | Collagen, type VII, alpha 1 | 204136_at | 195 ± 258 | 1.0 |
| *C1QTNF3* | C1q and tumor necrosis factor related protein 3 | 220988_s_at | 190 ± 623 | 1.2 |
| *C1QTNF1* | C1q and tumor necrosis factor related protein 1 | 220975_s_at | 146 ± 50 | -1.1 |
| *EMILIN1* | Elastin microfibril interfacer 1 | 204163_at | 144 ± 210 | 5.8 |
| *COL5A1* | Collagen, type V, alpha 1 | 203325_s_at | 128 ± 295 | 1.3 |
| *COL6A3* | Collagen, type VI, alpha 3 | 201438_at | 120 ± 468 | 1.1 |
| *COL11A2* | Collagen, type XI, alpha 2 | 216993_s_at | 119 ± 24 | 1.0 |
| *C1QTNF5 / MFRP* | C1q and tumor necrosis factor related protein 5 / membrane frizzled-related protein | 224286_at | 112 ± 11 | 1.0 |
| *COL26A1* | Collagen, type XXVI, alpha 1 | 233894_x_at | 105 ± 13 | 1.0 |
| *EDA* | Ectodysplasin A | 206217_at | 85 ± 13 | 1.0 |

*WM793 and WM239 melanoma cell lines.
