# Supplementary material for: Prolyl 4‐hydroxylase subunit alpha 1 (P4HA1) is a biomarker of poor prognosis in primary melanomas, and its depletion inhibits melanoma cell invasion and disrupts tumor blood vessel walls
Source: Mol Oncol. 2020 Feb 28;14(4):742–62. doi: 10.1002/1878-0261.12649 (PMC7138405; doi:10.1002/1878-0261.12649)
Supplement: Supplementary file 23 — Table S8. Genes correlating with P4HA1 expression in a panel of 62 melanoma cell lines (E‐GEOD‐7127). [file MOL2-14-742-s023.pdf]

Table S8. Genes correlating with P4HA1 expression in a panel of 62 melanoma cell lines (E-GEOD-7127).

Gene	Gene description	Probe set ID	Pearson correlation
P4HA1	Prolyl 4-hydroxylase subunit alpha 1	207543_s_at	1.000
MIR210HG	MIR210 host gene	230710_at	0.607
FUT11	Fucosyltransferase 11	226348_at	0.581
AK4	Adenylate kinase 4	230630_at	0.561
P4HA2	Prolyl 4-hydroxylase subunit alpha 2	202733_at	0.558
TMEM263	Transmembrane protein 263	224759_s_at	0.553
TMEM45A	Transmembrane protein 45A	219410_at	0.551
BNIP3	BCL2 interacting protein 3	201849_at	0.536
ANGPTL4	Angiopoietin-like 4	221009_s_at	0.530
DDIT4	DNA-damage-inducible transcript 4	202887_s_at	0.522
ITGB1	Integrin subunit beta 1	216178_x_at	0.513
HTR2A	5-hydroxytryptamine receptor 2A	244130_at	0.512
ALDH18A1	Aldehyde dehydrogenase 18 family, member A1	222416_at	0.508
VEGFA	Vascular endothelial growth factor A	210512_s_at	0.504
PLOD1	Procollagen-lysine, 2-oxoglutarate 5-dioxygenase 1	200827_at	0.491
TWSG1	Twisted gastrulation BMP signaling modulator 1	225406_at	0.485
UBTD1	Ubiquitin domain containing 1	219172_at	0.479
HYPK /// MIR1282 /// SERF2	Huntingtin interacting protein K /// MicroRNA 1282 /// Small EDRK-rich factor 2	226692_at	0.476
HIF1A	Hypoxia inducible factor 1, subunit alpha	200989_at	0.473
SLC22A3	Solute carrier family 22, member 3	205421_at	0.473
RHCE /// RHD	Rh blood group, CcEe antigens /// Rh blood group, D antigen	215819_s_at	0.468
RSPH3	Radial spoke head 3 homolog	229810_at	0.468
KGFLP2	Fibroblast growth factor 7 pseudogene 3	231031_at	0.467
TAF9B	TATA box binding protein associated factor 9b	228483_s_at	0.467
HSPA13	Heat shock protein family A (Hsp70) member 13	202557_at	0.465
TNFAIP6	TNF alpha induced protein 6	206026_s_at	0.463
CNIH4	Cornichon family AMPA receptor auxiliary protein 4	228437_at	0.462
ANKRD37	Ankyrin repeat domain 37	227337_at	0.460
ZNF432	Zinc finger protein 432	219848_s_at	0.459
SLC35B3	Solute carrier family 35, member B3	222691_at	0.459
REEP3	Receptor accessory protein 3	225785_at	0.455
PDK1	Pyruvate dehydrogenase kinase 1	226452_at	0.454
HTR7P1	5-hydroxytryptamine receptor 7 pseudogene 1	236115_at	0.450
SSR1	signal sequence receptor subunit 1	200891_s_at	0.450
SGCB	sarcoglycan, beta	226112_at	0.449
SLC16A1	solute carrier family 16, member 1	202234_s_at	0.448
SLC44A2	solute carrier family 44, member 2	225175_s_at	0.448
IRAK3	interleukin-1 receptor-associated kinase 3	213817_at	0.448
PLAGL1	PLAG1 like zinc finger 1	207943_x_at	0.445
ENC1	ectodermal-neural cortex 1	201341_at	0.444
ADAMTS1	ADAM metallopeptidase with thrombospondin type 1 motif 1	222162_s_at	0.444
THAP2	THAP domain containing 2	223588_at	0.444
ALDH1L2	aldehyde dehydrogenase 1 family, member L2	231202_at	0.442
