# Supplementary material for: Prolyl 4‐hydroxylase subunit alpha 1 (P4HA1) is a biomarker of poor prognosis in primary melanomas, and its depletion inhibits melanoma cell invasion and disrupts tumor blood vessel walls
Source: Mol Oncol. 2020 Feb 28;14(4):742–62. doi: 10.1002/1878-0261.12649 (PMC7138405; doi:10.1002/1878-0261.12649)
Supplement: Supplementary file 24 — Table S9. Gene Set Enrichment Analysis results for genes that correlate with P4HA1 expression in melanoma cell lines and primary melanoma tissues. [file MOL2-14-742-s024.pdf]

Table S9. Gene Set Enrichment Analysis results for genes that correlate with P4HA1 expression in melanoma cell lines and primary melanoma tissues.

**Gene set collection**	**Gene set**	**Normalized enrichment score**	**False discovery rate q-value**
Melanoma cell lines			
HALLMARK	EPITHELIAL_MESENCHYMAL_TRANSITION	3.839	<0.0001
	HYPOXIA	3.285	<0.0001
	GLYCOLYSIS	2.607	<0.0001
	MTORC1_SIGNALING	2.014	0.008
	INFLAMMATORY_RESPONSE	1.758	0.024
Primary melanoma tissues			
HALLMARK	HYPOXIA	3.049	<0.0001
	GLYCOLYSIS	2.527	<0.0001
	MTORC1_SIGNALING	2.439	<0.0001
