# Supplementary material for: Prolyl 4‐hydroxylase subunit alpha 1 (P4HA1) is a biomarker of poor prognosis in primary melanomas, and its depletion inhibits melanoma cell invasion and disrupts tumor blood vessel walls
Source: Mol Oncol. 2020 Feb 28;14(4):742–62. doi: 10.1002/1878-0261.12649 (PMC7138405; doi:10.1002/1878-0261.12649)
Supplement: Supplementary file 25 — Table S10. Gene expression changes in WM239 cells after knockdown of P4HA1 expression. [file MOL2-14-742-s025.pdf]

**Table S10.** Gene expression changes in WM239 cells after knockdown of P4HA1 expression.

| Gene | Gene description | Wald statistic | Adjusted p-value | Normalized counts ± StDev | | Fold |
|---|---|---|---|---|---|---|
| | | | | WM239 parental and control shRNA cells (*n* = 4) | WM239 P4HA1-knockdown cells (*n* = 4) | |
| Downregulated in P4HA1-knockdown cells | | | | | | |
| *P4HA1* | Prolyl 4-hydroxylase subunit alpha 1 | -18.352 | 3.01E-71 | 2476 ± 457 | 474 ± 37 | -5.23 |
| *LAMA5* | Laminin subunit alpha 5 | -5.582 | 7.51E-05 | 1096 ± 167 | 793 ± 179 | -1.38 |
| *SPIRE1* | Spire type actin nucleation factor 1 | -5.076 | 0.00074 | 608 ± 92 | 420 ± 15 | -1.45 |
| *MT-RNR2* | Mitochondrially encoded 16S RNA | -4.460 | 0.011 | 95597 ± 10123 | 68555 ± 9427 | -1.39 |
| *LPL* | Lipoprotein lipase | -4.336 | 0.017 | 4310 ± 1214 | 2993 ± 543 | -1.44 |
| *RELN* | Reelin | -4.133 | 0.038 | 339 ± 130 | 197 ± 67 | -1.72 |
| Upregulated in P4HA1-knockdown cells | | | | | | |
| *KCNE4* | Potassium voltage-gated channel subfamily E regulatory subunit 4 | 8.844 | 4.38E-15 | 617 ± 75 | 1145 ± 240 | 1.86 |
| *DKK1* | Dickkopf WNT signaling pathway inhibitor 1 | 5.075 | 0.00074 | 1016 ± 192 | 1358 ± 277 | 1.34 |
| *HERPUD1* | Homocysteine inducible ER protein with ubiquitin like domain 1 | 4.594 | 0.0069 | 1084 ± 72 | 1485 ± 308 | 1.37 |
| *F2RL2* | Coagulation factor II thrombin receptor like 2 | 4.059 | 0.047 | 1142 ± 165 | 1515 ± 175 | 1.33 |
